# Pd-Catalyzed [4 + 1] Annulation Strategy to Functionalized 4-Methyleneproline Derivatives

**DOI:** 10.1021/acs.joc.3c02178

**Published:** 2024-01-23

**Authors:** Jiaxin Han, Wenzheng Gao, Joseph P. A. Harrity

**Affiliations:** Department of Chemistry, University of Sheffield, Sheffield S3 7HF, U.K.

## Abstract

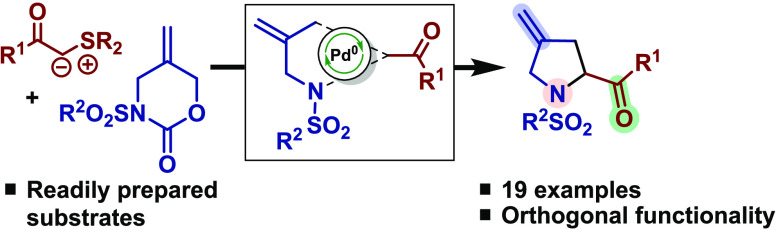

A Pd-catalyzed formal [4 + 1] cycloaddition reaction of sulfur ylides and in situ-generated Pd-stabilized zwitterions offers a convenient route to a series of functionalized proline derivatives. The utility of this method is demonstrated by a gram-scale synthesis and chemoselective functionalization of a proline-based derivative.

Amphiphilic allylation reactions and related processes offer a promising strategy for the rapid construction of heterocyclic compounds through formal cycloaddition type strategies.^1^ The design of metal-stabilized intermediates that mediate these processes offers a platform to devise transition metal-catalyzed transformations and typically endows the corresponding products with useful functionality for downstream elaboration. In this regard, Trost’s Pd-trimethylenemethane reagents serve as an exemplar for the synthesis of carbocyclic and heterocyclic scaffolds.^2^

Inspired by the synthetic potential of Pd-stabilized zwitterions, we and others have recently shown that *N*^1^-1,4-dipole equivalents can be generated in situ in the presence of Pd-catalysts and exploited in the synthesis of functionalized piperidines.^3^ As shown in Scheme 1, we speculated that the use of a carbene equivalent in place of an enolate surrogate could allow us to access pyrrolidines in place of the already established piperidine chemistry.^4^ Pyrrolidines are ranked the top 5 nitrogen heterocycles in FDA-approved pharmaceuticals^5^ and are a key residue in controlling protein folding.^6^ Furthermore, within this particular class, 4-methyleneproline has emerged as an important motif, as it is found in inhibitors of proline dehydrogenase and tomaymycin analogues.^7^

**Scheme 1:**
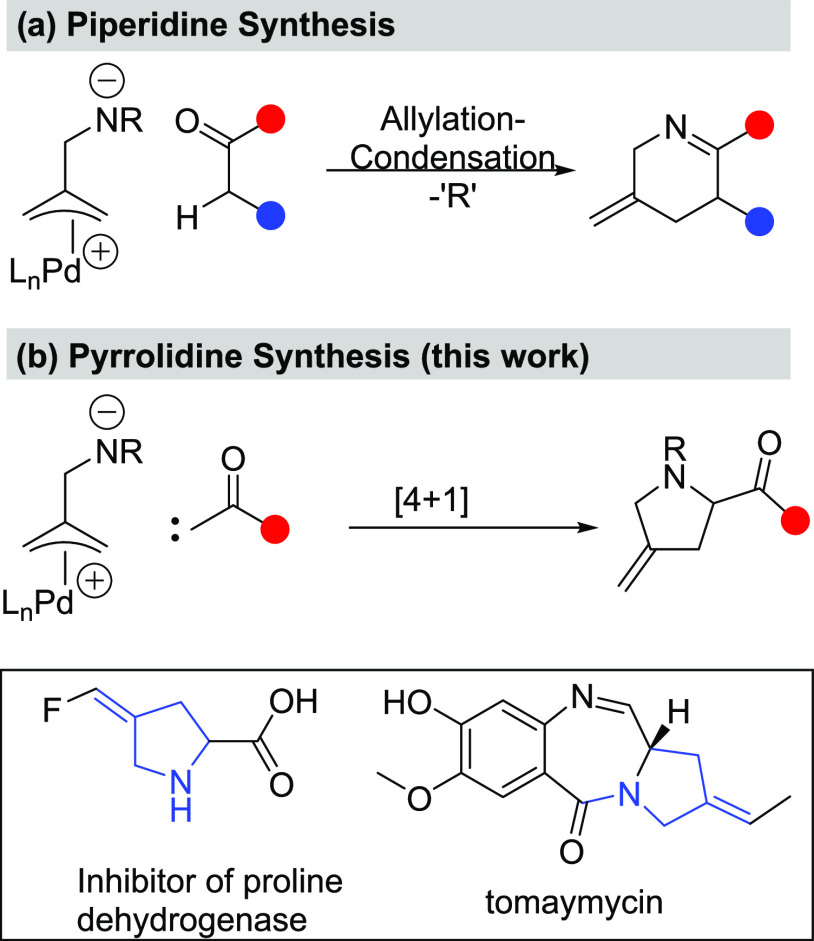
Amphiphilic Allylation Strategy to *N*-Heterocycles

We identified sulfur ylides as potential carbene surrogates because of their intrinsic nucleophilic and electrophilic properties. Moreover, their use in formal cycloaddition processes has begun to emerge that confirmed their compatibility with Pd-catalysis.^8^ We report herein the successful realization of a formal [4 + 1] cycloaddition strategy^9^ that provides functionalized pyrrolidines from readily available starting materials.

## Results and Discussion

In order to confirm the viability of the proposed transformation, we screened a range of Pd/ligand combinations in an effort to promote the reaction of carbamate **1** with sulfur ylide **2a**, and selected results are shown in Table 1. Pd(PPh_3_)_4_ failed to produce **3a** at room temperature (entry 1), whereas Pd(dba)_2_, in conjunction with **L1**, gave a low conversion to the desired pyrrolidine (entry 2). Increasing the reaction temperature resulted in an improvement in conversion, and **3a** was isolated in 48% yield (entry 3). We were concerned that free dba ligand could consume the sulfur ylide via cyclopropanation and so switched instead to [η^3^-(C_3_H_5_)PdCl]_2_ as a precatalyst. Pleasingly, this reaction proceeded smoothly to generate the desired product in 61% yield (entry 4). Exploring a range of alternative solvents (e.g., PhMe, NMP, DCE) failed to improve matters and so we opted to explore the scope of this method under these conditions.

**Table 1:**
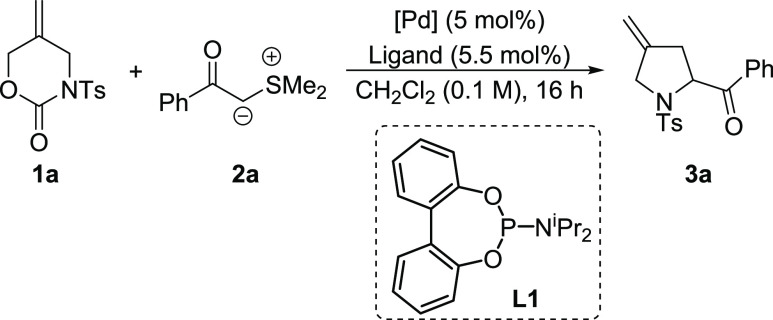
Catalyst Optimization Studies

entry^a^	[Pd]	ligand	*T* (°C)	yield (%)
1	Pd(PPh_3_)_4_	--	rt	0
2	Pd(dba)_2_	L1	rt	10
3	Pd(dba)_2_	L1	50	48
4	[η^3^-(C_3_H_5_)PdCl]_2_	L1	50	61

a Carbamate **1a** (1.0 equiv), L1 (5.5 mol %), and [Pd] 5 mol % stirred in anhydrous CH_2_Cl_2_ (0.5 mL per mmol of carbamate) at rt for 15 min, followed by addition of a solution of **2a** (1.5 equiv) in CH_2_Cl_2_ (0.5 mL per mmol of carbamate) and the mixture stirred overnight at rt or 50 °C.

**Scheme 2:**
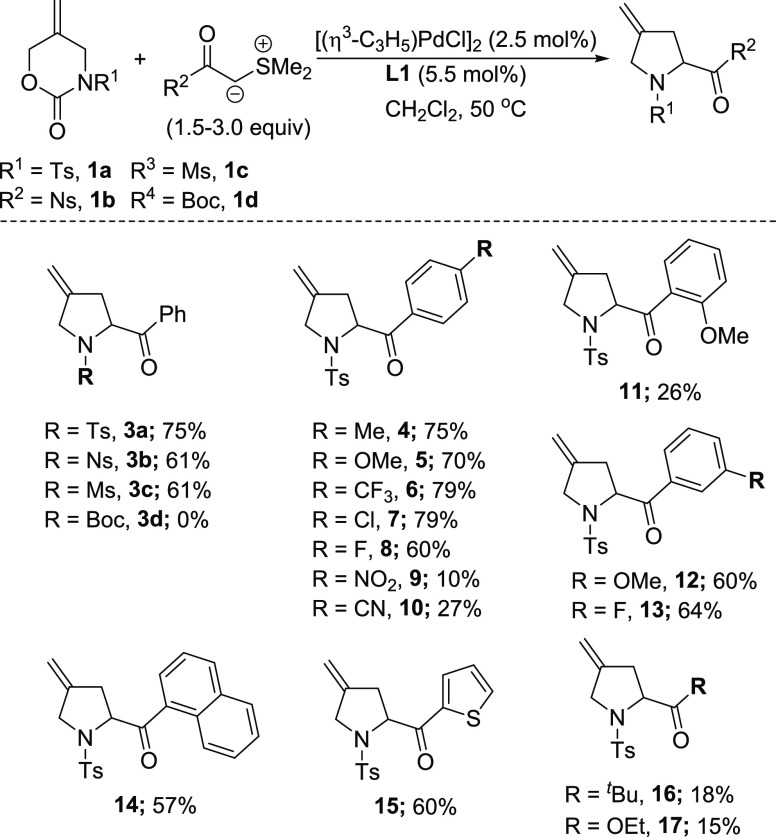
Scope of the [4 + 1] Annulation; Carbamate **1a**–**d** (1.0 Equiv), L1 (5.5 mol %), and [η^3^-(C_3_H_5_)PdCl]_2_ 2.5 mol % Strirred in Anhydrous CH_2_Cl_2_ (0.5 mL per mmol of Carbamate) at rt for 15 min, Followed by Addition of a Solution of Ylide (1.5–3.0 Equiv) in CH_2_Cl_2_ (0.5 mL per mmol of Carbamate) and the Mixture Stirred Overnight at 50 °C

We first explored the scope of the method with respect to zwitterion precursor **1**. While the use of sulfone functional groups tosyl (Ts), nosyl (Ns), and mesyl (Ms) **1a**–**c** were broadly effective, the reaction failed when using a Boc-containing analogue, and only **1d** was recovered. Next, we investigated the scope of the Pd-catalyzed reaction of carbamate **1a** with sulfur ylides. The reactions of *para*-substituted aromatic sulfur ylides containing both electron-donating **4**–**5** and weak electron-withdrawing groups **6**–**8** were successful, giving similar yields (65–79%). More strongly electron-withdrawing groups were less effective, leading to complex crude reaction mixtures from which **9**–**10** were isolated in low yields. A similar outcome was observed in the case of the *ortho*-MeO containing example **11**. Ylides bearing *meta*-substituents, as well as 1-naphthyl and thiophene groups, afforded the corresponding products **12**–**15** in acceptable yields. In contrast, pyrrolidines prepared from ylides featuring alkyl and ester functional groups **16**–**17** did not proceed efficiently (Scheme 2).

**Scheme 3:**
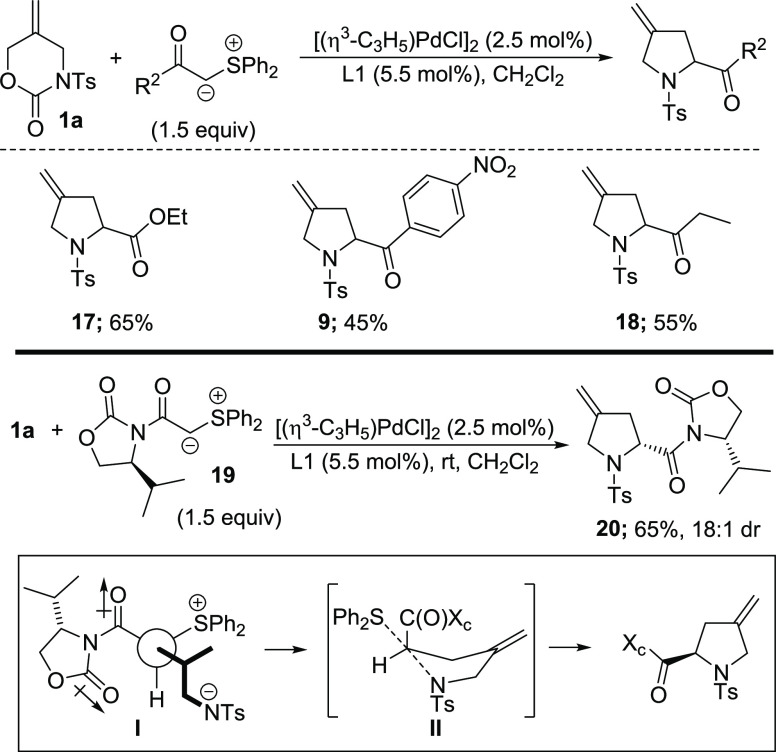
Reactivity of Diphenyl Sulfur Ylides

The low yields observed in the formation of **17** (Scheme 2) were particularly disappointing, as the potential to exploit this chemistry in the synthesis of proline derivatives was a key objective. Therefore, we speculated that the reactivity of the corresponding ylide might be modulated by altering the nature of the sulfonium cation. To this end, we changed to the corresponding diphenyl sulfur ylide and were pleased to find that the reaction proceeded smoothly at room temperature to generate the desired product **17** in 69% yield. Interestingly, the diphenylsulfonium group also delivered generally enhanced yields in the case of aromatic ketone **9** and alkyl-substituted ketone **18** as compared with the dimethyl sulfur ylides. Encouraged by this result, we prepared sulfur ylide **19** bearing an oxazolidinone auxiliary in an effort to control stereochemistry at C2. Pleasingly, this also underwent the [4 + 1] annulation to provide proline derivative **20** in 76% yield with 18:1 dr. Regarding the source of diastereocontrol in this case, our working hypothesis is that this originates from the addition of the Pd π-allyl complex to the open face of the enolate **I**, followed by cyclization via **II** (Scheme 3).

**Scheme 4:**
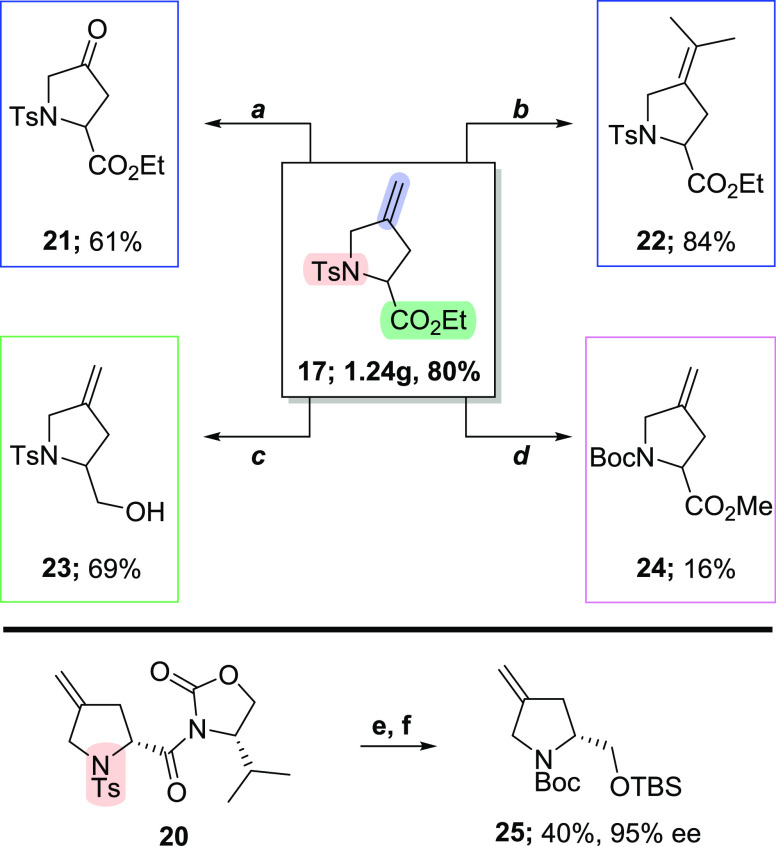
Chemoselective Functionalization of Proline Derivatives **17** and **20** Reagents and conditions: (a) RuCl_3_ (0.3 equiv), NaIO_4_ (8 equiv), MeCN/DCM/H_2_O (1:1:2 v/v/v), 0 °C to rt, 4 h; (b) H-G II (10 mol %), 2-methyl-2-butene, Ti(OiPr)4 (30 mol %), DCE, 50 °C, 16 h; (c) LiAlH4 (1.2 equiv), THF, 0 °C, 1 h; (d) Mg (70 equiv), MeOH, ultrasonication, 6 h; Boc_2_O (2.0 equiv), TEA (2.2 equiv), DMAP (10 mol %), DCM, rt, 16 h; (e) LiAlH_4_ (1.0 equiv), THF, 0 °C, 1 h; TBSCl (1.1 equiv), imidazole (1.1 equiv), DCM, rt, 16 h; and (f) Mg (70 equiv), MeOH, ultrasonication, 60 °C, 6 h; Boc_2_O (2.0 equiv), TEA (2.2 equiv), DMAP (10 mol %), DCM, rt, overnight.

Finally, we wanted to investigate the synthetic versatility of these compounds. Accordingly, we performed the gram-scale synthesis of **17** that served to highlight the scalability of this method while delivering material to explore further functionalization reactions. In this regard, the alkene underwent efficient Ru-catalyzed oxidative cleavage to obtain **21** under mild conditions. In addition, olefin metathesis generated the corresponding dimethyl substituted olefin **22** in the presence of catalytic amount of Hoveyda–Grubbs second generation catalyst and Ti(O*i*Pr)_4_.^10^ Reduction of **17** provided alcohol **23** in high yield, while deprotection of the Ts group was achieved using Mg/MeOH that also led to transesterification to generate **24**. The deprotected intermediate was conveniently isolated as Boc derivative **24**, albeit in a low overall yield. Interestingly, attempts to remove the Ts group in **20** using Mg/MeOH led to racemization; however, **20** could be converted to Boc-protected pyrrolidine **26** with high stereochemical retention while at the same time confirming the absolute stereochemistry at C2 (see the Supporting Information for details) (Scheme 4).

## Conclusions

In summary, we have described a robust and versatile palladium-catalyzed [4 + 1] annulation for the synthesis of 4-methyleneproline derivatives. These compounds have the potential for orthogonal functionalization, and this, together with their low molecular weight, makes them a useful class of scaffolds for early-stage drug discovery programs.

## Data Availability

The data underlying this study are available in the published article and its online Supporting Information.

## Supporting Information Available

Details of experimental procedures and spectroscopic data; NMR spectral data are included (PDF)
