# Supplementary material for: Pd-Catalyzed [4 + 1] Annulation Strategy to Functionalized 4-Methyleneproline Derivatives
Source: J Org Chem. 2024 Jan 23;89(3):1552–5. doi: 10.1021/acs.joc.3c02178 (PMC10845110; doi:10.1021/acs.joc.3c02178)
Supplement: Supplementary file 1 — jo3c02178_si_001.pdf [file jo3c02178_si_001.pdf]

Supporting Information

Pd-Catalyzed [4 + 1] Annulation Strategy to Functionalized 4-Methyleneproline Derivatives

Jiaxin Han, Wenzheng Gao, and Joseph P. A. Harrity*

Department of Chemistry, University of Sheffield, Sheffield, S3 7HF, United Kingdom

* *Email: j.harrity@sheffield.ac.uk*

Table of contents

Experimental Section **S2**

General Considerations **S2**

General Procedures A-D **S3**

Ylide Syntheses **S4**

Cycloaddition Reactions **S10**

Gram scale synthesis of compound 17 **S20**

Functionalization Reactions **S20**

NMR Spectra **S25**

HPLC Traces **S53**

References **S55**

Experimental Section

General Considerations

All reactions were carried out in flame-dried glassware equipped with a magnetic stir bar under nitrogen atmosphere, unless stated otherwise. Solvents were purified using a PureSolv MD purification system and transferred under nitrogen. A DrySyn block combined with a temperature probe was used as the heating source, where required. Infrared (IR) spectra were recorded on a Perkin Elmer Paragon FTIR spectrometer (v_{max} in cm^{-1}). Samples were recorded neat as thin films. ^{1}H NMR spectra were recorded on a Bruker AVIII HD 400 (400 MHz), Bruker AVI 400 (400 MHz) or Bruker AMX400 (400 MHz). Chemical shifts are reported in parts per million (ppm) from tetramethylsilane, using the residual protic solvent resonance as the internal reference: ($CHCl_3$: δ 7.26) unless otherwise stated. Data are reported as follows: chemical shift (integration, multiplicity (s = singlet, d = doublet, t = triplet, q = quartet, br = broad, m = multiplet), coupling constant (Hz)). ^{13}C NMR spectra were recorded on a Bruker AVIII HD 400 (101 MHz), Bruker AVI 400 (101 MHz) or Bruker AMX-400 (101 MHz) with broadband proton decoupling. Chemical shifts are reported in ppm from trimethylsilane with the solvent as the internal reference ($CDCl_3$: δ 77.16). ^{19}F NMR spectra were recorded on a Bruker AV III HD 400 (377 MHz) and are uncorrected. High resolution mass spectra (HRMS) recorded for accurate mass analysis, were performed on either a Micromass LCT operating in electrospray mode (TOF, ES^+) or a Micromass Prospec operating in FAB (FAB^+), EI (EI^+) or CI (CI^+) mode. Thin layer chromatography (TLC) was performed on aluminium-backed plates pre coated with silica (0.2 mm, Merck 60 F_{254}) which were developed using standard visualizing agents: UV light or potassium permanganate. Flash chromatography was performed on silica gel (Merck 40-63 µm). Melting points were recorded on Gallenkamp melting point apparatus and are uncorrected.

General Procedure A (GPA)

To a solution of bromoketone (1.0 equiv.) in acetone (0.5 M), dimethyl sulfide (1.2 equiv.) was added and the mixture was stirred for 48 h at rt. Then, the sulfonium salt was collected by filtration, washed with acetone, and dried under high vacuum to afford the product salt that was used without further purification.

To a suspension of sulfonium salt (1.0 equiv.) in DCM (0.3 M) was added a solution of NaOH (5.0 equiv.) in H_2O (0.3 M) and the mixture was vigorously stirred for 1 h at room temperature. The product was extracted with EtOAc, the extract dried over anhydrous $MgSO_4$, filtered, and concentrated under high vacuum to afford the sulfur ylide that was used without further purification.

General Procedure B (GPB)

A flamed dried flask was charged with bromide (1.0 equiv.), diphenyl sulfide (2.0 equiv.) and $AgBF_4$ (1.0 equiv). Anhydrous $CHCl_3$ (0.5 M) was added and the reaction mixture was stirred at 85 °C overnight. The resulting mixture was then filtered through celite and washed with DCM. The filtrate was concentrated, and the residue was purified by flash column chromatography to afford the desired sulfonium salt.

To a solution of sulfonium salt (1.0 equiv) in anhydrous THF (0.2 M) was added NaH (2.0 equiv, 60 wt% in mineral oil) portionwise at 0 °C. The resulting suspension was slowly warmed to room temperature and stirred for 2 hours. Then, the reaction mixture was filtered through celite and washed with DCM. The desired product was afforded after evaporation and used without further purification.

Note: The use of anhydrous THF is essential for the generation of pure diphenyl sulfonium ylide products. In addition, the diphenyl sulfonium ylides decompose at room temperature and should be stored in a freezer. It's also important to check the quality of ylide by NMR spectroscopy before its use.

General Procedure C (GPC)

A flame-dried flask was charged with carbamate (1.0 equiv.), *N,N*-diisopropyldibenzo[d,f][1,3,2]dioxaphosphepin-6-amine (5.5 mol%), $[(\eta^3\text{-}C_3H_5)PdCl]_2$ (2.5 mol%),

under nitrogen. Anhydrous DCM (0.5 mL per mmol of carbamate) was then added and the mixture stirred at rt for 15 minutes. Dimethyl sulfur ylide (1.5 equiv. or 3 equiv.) in DCM (0.5 mL per mmol of carbamate) was added to the mixture and the reaction mixture was stirred overnight at 50 °C. The resulting mixture was then concentrated under vacuum and purified by flash column chromatography.

General Procedure D (GPD)

A flame-dried flask was charged with 5-methylene-3-tosyl-1,3-oxazinan-2-one (1.0 equiv.), *N,N*-diisopropyldibenzo[d,f][1,3,2]dioxaphosphepin-6-amine (5.5 mol%) and $[(\eta^3\text{-}C_3H_5)PdCl]_2$ (2.5 mol%) under nitrogen. Anhydrous DCM (0.5 mL per mmol of carbamate) was then added, and the mixture stirred at rt for 15 minutes. Diphenyl sulfur ylide (1.5 equiv.) in DCM (0.5 mL per mmol of ylide) was added to the mixture and the reaction mixture was stirred overnight at rt or 50°C. If incomplete conversion was observed, a further portion of *N,N*-diisopropyldibenzo[d,f][1,3,2]dioxaphosphepin-6-amine (5.5 mol%), $[(\eta^3\text{-}C_3H_5)PdCl]_2$ (2.5 mol%) and diphenyl sulfur ylide (0.5 equiv.) were added and the reaction mixture was stirred for another 2 h at rt or 50°C. The resulting mixture was then concentrated under vacuum and purified by flash silica column chromatography.

2-(dimethyl-λ⁴-sulfaneylidene)-1-phenylethan-1-one (2a)[1]

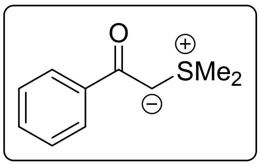

Following the General Procedure A with 2-bromoacetophenone (1.99 g, 10 mmol), dimethyl sulfide (0.745 g, 12 mmol) in acetone (20 mL). NaOH (2.0 g, 50 mmol) in H_2O (33 mL) was added to the generated sulfonium salt in DCM (33 mL), which afforded 2-(dimethyl-λ^4-sulfaneylidene)-1-phenylethan-1-one (**2a**) as a yellow solid (0.93 g, 51%).

^{1}H NMR (400 MHz, $CDCl_3$): δ 7.53 – 7.51 (m, 2H), 7.11 – 6.98 (m, 3H), 4.04 (s, 1H), 2.52 (s, 6H).

^{13}C{^{1}H} NMR (101 MHz, $CDCl_3$): δ 181.6, 140.6, 129.0, 127.5, 125.9, 53.1, 28.2.

2-(dimethyl-λ⁴-sulfaneylidene)-1-(*p*-tolyl)ethan-1-one (2b)[1]

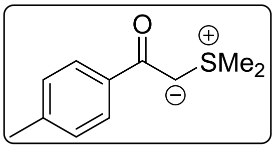

Following the General Procedure A with 2-bromo-4'-methylacetophenone (2.13 g, 10 mmol), dimethyl sulfide (0.745 g, 12

mmol) in acetone (20 mL). NaOH (2.0 g, 50 mmol) in H_2O (33 mL) was added to the generated sulfonium salt in DCM (33 mL), afforded 2-(dimethyl-λ4-sulfaneylidene)-1-(*p*-tolyl)ethan-1-one (**2b**) as a yellow solid (1.10 g, 57%).

^{1}H NMR (400 MHz, $CDCl_3$): δ 7.66 (d, *J* = 8.0 Hz, 2H), 7.13 (d, *J* = 8.0 Hz, 2H), 4.27 (s, 1H), 2.95 (s, 6H), 2.33 (s, 3H). **$^{13}C\{^1H\}$ NMR (101 MHz, $CDCl_3$)**: δ 183.5, 139.8, 138.5, 128.9, 126.6, 50.4, 28.9, 21.6.

2-(dimethyl-λ4-sulfaneylidene)-1-(4-methoxyphenyl)ethan-1-one (2c)[1]

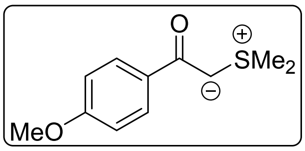

Following the General Procedure A with 2-bromo-1-(4-methoxyphenyl)ethan-1-one (2.29 g, 10 mmol), dimethyl sulfide (0.745 g, 12 mmol) in acetone (20 mL). NaOH (2.0 g, 50 mmol) in H_2O (33 mL) was added to the generated sulfonium salt in DCM (33 mL), afforded 2-(dimethyl-λ4-sulfaneylidene)-1-(4-methoxyphenyl)ethan-1-one (**2c**) as a yellow solid (0.744 g, 36%).

^{1}H NMR (400 MHz, $CDCl_3$): δ 7.73 (d, *J* = 9.0 Hz, 2H), 6.83 (d, *J* = 9.0 Hz, 2H), 4.24 (s, 1H), 3.79 (s, 3H), 2.96 (s, 6H). **$^{13}C\{^1H\}$ NMR (101 MHz, $CDCl_3$):** δ 183.0, 161.1, 134.0, 128.2, 113.3, 55.5, 50.0, 28.9.

2-(dimethyl-λ4-sulfaneylidene)-1-(4-(trifluoromethyl)phenyl)ethan-1-one (2d)[2]

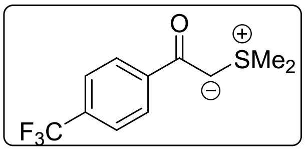

Following the General Procedure A with 2-bromo-1-(4-(trifluoromethyl)phenyl)ethan-1-one (2.67 g, 10 mmol), dimethyl sulfide (0.745 g, 12 mmol) in acetone (20 mL). NaOH (2.0 g, 50 mmol) in H_2O (33 mL) was added to the generated l sulfonium salt in DCM (33 mL), afforded 2-(dimethyl-λ4-sulfaneylidene)-1-(4-(trifluoromethyl)phenyl)ethan-1-one (**2d**) as a yellow solid (1.18 g, 48%).

^{1}H NMR (400 MHz, $CDCl_3$): δ 7.84 (d, *J* = 8.0 Hz, 2H), 7.57 (d, *J* = 8.0 Hz, 2H), 4.32 (s, 1H), 2.97 (s, 6H); **^{19}F NMR (376 MHz, $CDCl_3$):** δ -62.5; **$^{13}C\{^1H\}$ NMR (101 MHz, $CDCl_3$):** δ 181.6, 144.5, 131.3 (q, *J* = 31.5 Hz), 126.9, 125.4, 124.5 (q, *J* = 272.5 Hz), 52.7, 28.5.

2-(dimethyl-λ4-sulfaneylidene)-1-(4-chlorophenyl)-ethan-1-one (2e)[2]

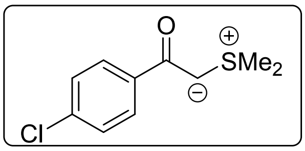

Following the General Procedure A with 2-bromo-1-(4-chlorophenyl)ethan-1-one (2.33 g, 10 mmol), dimethyl sulfide (0.745

g, 12 mmol) in acetone (20 mL). NaOH (2.0 g, 50 mmol) in H_2O (33 mL) was added to the generated sulfonium salt in DCM (33 mL), afforded 1-(4-chlorophenyl)-2-(dimethyl-λ^4-sulfaneylidene)ethan-1-one (**2e**) as an orange solid (1.60 g, 74%).

^{1}H NMR (400 MHz, $CDCl_3$): δ 7.78 – 7.67 (m, 2H), 7.37 – 7.26 (m, 2H), 4.28 (s, 1H), 2.97 (s, 6H).

^{13}C{^{1}H} NMR (101 MHz, $CDCl_3$): δ 181.7, 139.6, 135.5, 128.2, 128.0, 51.7, 28.6.

2-(dimethyl-λ^4-sulfaneylidene)-1-(4-fluorophenyl)ethan-1-one (2f)[2]

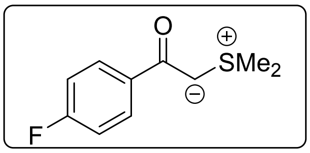

Following the General Procedure A with 2-bromo-1-(4-fluorophenyl)ethan-1-one (2.17 g, 10 mmol), dimethyl sulfide (0.745 g, 12 mmol) in acetone (20 mL). NaOH (2.0 g, 50 mmol) in H_2O (33 mL) was added to the generated l sulfonium salt in DCM (33 mL), afforded 2-(dimethyl-λ^4-sulfaneylidene)-1-(4-fluorophenyl)ethan-1-one (**2f**) as an orange solid (1.04 g, 53%).

^{1}H NMR (400 MHz, $CDCl_3$): δ 7.76 – 7.73 (m, 2H), 7.01 – 6.96 (m, 2H), 4.24 (s, 1H), 2.96 (s, 6H); **^{19}F NMR (377 MHz, $CDCl_3$):** δ -112.3; **^{13}C{^{1}H} NMR (101 MHz, $CDCl_3$):** δ 182.2, 164.0 (d, *J* = 248.0 Hz), 137.4, 128.6 (d, *J* = 8.5 Hz), 114.9 (d, *J* = 21.5 Hz), 51.0, 28.8.

4-(2-(dimethyl-λ^4-sulfaneylidene)acetyl)benzonitrile (2h)[2]

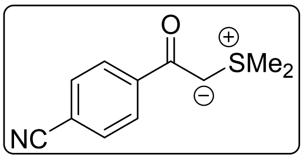

Following the General Procedure A with 4-(2-bromoacetyl)benzonitrile (2.24 g, 10 mmol), dimethyl sulfide (0.745 g, 12 mmol) in acetone (20 mL). NaOH (2.0 g, 50 mmol) in H_2O (33 mL) was added to the generated l sulfonium salt in DCM (33 mL), afforded 4-(2-(dimethyl-λ^4-sulfaneylidene)acetyl)benzonitrile (**2h**) a pale yellow solid (0.493 g, 24%).

^{1}H NMR (400 MHz, $CDCl_3$): δ7.76 (d, *J* = 8.0 Hz, 2H), 7.54 (d, *J* = 8.0 Hz, 2H), 4.28 (s, 1H), 2.91 (s, 6H); **^{13}C{^{1}H} NMR (101 MHz, $CDCl_3$):** δ 180.4, 145.2, 131.9, 127.1, 119.1, 112.6, 54.0, 28.0.

2-(dimethyl-λ^4-sulfaneylidene)-1-(3-methoxyphenyl)ethan-1-one (2j)[3]

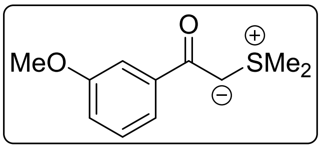

Following the General Procedure A with 2-bromo-1-(3-methoxyphenyl)ethan-1-one (2.29 g, 10 mmol), dimethyl sulfide (0.745 g, 12 mmol) in acetone (20 mL). NaOH (2.0 g, 50 mmol) in H_2O (33 mL) was added to the generated sulfonium salt in DCM (33 mL), afforded 2-(dimethyl-λ^4-

sulfaneylidene)-1-(3-methoxyphenyl)ethan-1-one (**2j**) as an orange oil (1.53 g, 73%).

^{1}H NMR (400 MHz, CDCl$_3$): δ 7.42-7.71 (m, 1H), 7.34-7.30 (m, 1H), 7.26-7.22 (m, 1H), 6.93-6.91 (m, 1H), 4.34 (s, 1H), 3.84 (s, 3H), 2.99 (s, 6H); **^{13}C{^{1}H} NMR (101 MHz, CDCl$_3$)**: δ 183.1, 159.7, 142.7, 129.1, 119.1, 116.4, 111.2, 55.6, 51.6, 28.7.

2-(dimethyl-λ^4-sulfaneylidene)-1-(3-fluorophenyl)ethan-1-one (2k)[4]

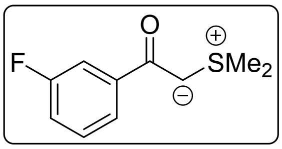

Following the General Procedure A with 2-bromo-1-(3-fluorophenyl)ethan-1-one (2.17 g, 10 mmol), dimethyl sulfide (0.745g, 12 mmol) in acetone (20 mL). NaOH (2.0 g, 50 mmol) in H_2O (33 mL) was added to the generated sulfonium salt in DCM (33 mL), afforded 2-(dimethyl-λ^4-sulfaneylidene)-1-(3-fluorophenyl)ethan-1-one (**2k**) as an orange oil (1.52 g, 77%).

^{1}H NMR (400 MHz, CDCl$_3$): δ 7.55-7.53 (m, 1H), 7.50-7.47 (m, 1H), 7.33-7.27 (m, 1H), 7.07-7.02 (m, 1H), 4.31 (s, 1H), 2.99 (s, 6H); **^{19}F NMR (377 MHz, CDCl$_3$):** δ -113.8 – -114.0 (m); **^{13}C{^{1}H} NMR (101 MHz, CDCl$_3$)**: δ 181.7, 163.0 (d, J = 245.0 Hz), 143.8, 129.6 (d, J = 8.0 Hz), 122.2 (d, J = 2.0 Hz), 116.5 (d, J = 22.0 Hz), 113.5 (d, J = 22.0 Hz), 52.0, 28.6.

2-(dimethyl-λ^4-sulfaneylidene)-1-(naphthalen-2-yl)ethan-1-one (2l)[1]

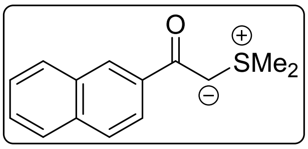

Following the General Procedure A with 2-bromo-1-(naphthalen-2-yl)ethan-1-one (2.49 g, 10 mmol), dimethyl sulfide (0.745 g, 12 mmol) in acetone (20 mL). NaOH (2.0 g, 50 mmol) in H_2O (33 mL) was added to the generated sulfonium salt in DCM (33 mL), afforded 2-(dimethyl-λ^4-sulfaneylidene)-1-(naphthalen-2-yl)ethan-1-one (**2l**) as an orange solid (1.13 g, 49%).

^{1}H NMR (400 MHz, CDCl$_3$): δ 8.32-8.28 (m, 1H), 7.92 – 7.84 (m, 2H), 7.82-7.78 (m, 2H), 7.49 – 7.42 (m, 2H), 4.45 (s, 1H), 2.99 (s, 6H); **^{13}C{^{1}H} NMR (101 MHz, CDCl$_3$):** δ 183.2, 138.5, 134.9, 133.3, 129.2, 127.8, 127.7, 126.7, 126.3, 126.2, 124.5, 51.7, 28.8.

2-(dimethyl-λ^4-sulfaneylidene)-1-(thiophen-2-yl)ethan-1-one (2m)[1]

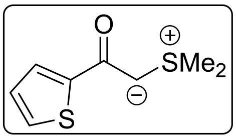

Following the General Procedure A with 2-bromo-1-(thiophen-2-yl)ethan-1-one (2.05 g, 10 mmol), dimethyl sulfide (0.745 g, 12 mmol) in acetone (20 mL). NaOH (2.0 g, 50 mmol) in H_2O (33 mL) was added to the generated

sulfonium salt in DCM (33 mL), afforded 2-(dimethyl-λ4-sulfaneylidene)-1-(thiophen-2-yl)ethan-1-one (**2m**) as a white solid (1.10 g, 59%).

^{1}H NMR (400 MHz, CDCl$_3$): δ 7.36 (d, *J* = 3.0 Hz, 1H), 7.28 (d, *J* = 5.0 Hz, 1H), 6.98 (dd, *J* = 5.0, 3.0 Hz, 1H), 4.22 (s, 1H), 2.99 (s, 6H); **^{13}C{^{1}H} NMR (101 MHz, CDCl$_3$):** δ 176.9, 147.9, 127.5 (2 carbons), 125.6, 50.0, 28.9.

1-(dimethyl-λ4-sulfaneylidene)-3,3-dimethylbutan-2-one (2n) [1]

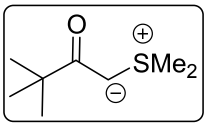

Following the General Procedure A with bromo pinacolone (1.79 g, 10 mmol), dimethyl sulfide (0.745 g, 12 mmol) in acetone (20 mL). NaOH (2.0 g, 50 mmol) in H_2O (33 mL) was added to the generated sulfonium salt in DCM (33 mL), afforded 1-(dimethyl-λ4-sulfaneylidene)-3,3-dimethylbutan-2-one **(2n)** as a white solid (0.248 g, 16%).

^{1}H NMR (400 MHz, CDCl$_3$): δ 3.70 (s, 1H), 2.80 (s, 6H), 1.06 (s, 9H); **^{13}C{^{1}H} NMR (101 MHz, CDCl$_3$):** δ 198.3, 48.5, 40.7, 29.0, 28.6.

ethyl 2-(diphenyl-λ4-sulfaneylidene)acetate (2p)[5]

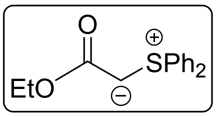

Following the General Procedure B with ethyl 2-bromoacetate (1.67 g, 10 mmol), diphenyl sulfide (3.72 g, 20 mmol), silver tetrafluoroborate (1.94 g, 10 mmol) in $CHCl_3$ (20 mL) after chromatography (gradient from 4-6% MeOH in DCM) afforded (2-ethoxy-2-oxoethyl) diphenyl sulfonium tetrafluoroborate as white solid (2.76 g, 77%). NaH (0.32 g, 8 mmol, 60 wt% in mineral oil) and (2-ethoxy-2-oxoethyl) diphenyl sulfonium tetrafluoroborate (1.44 g, 4 mmol) in anhydrous THF (25 mL), afforded ethyl 2-(diphenyl-λ4-sulfaneylidene)acetate (**2p**) as a sticky pale yellow oil (1.08 g, 99%).

^{1}H NMR (400 MHz, CDCl$_3$): δ 7.57-7.45 (m, 10H), 4.10 (q, *J* = 7.0 Hz, 2H), 3.47-3.22 (br, 1H), 1.25 (t, *J* = 7.0 Hz, 3H); **^{13}C{^{1}H} NMR (101 MHz, CDCl$_3$):** δ 170.4, 136.7, 131.1, 130.0, 128.3, 58.9, 36.2, 15.3.

2-(diphenyl-λ4-sulfaneylidene)-1-(4-nitrophenyl)ethan-1-one (2g)[6]

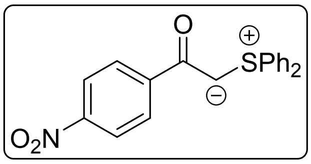

Following the General Procedure B with 1-bromobutan-2-one (2.44 g, 10 mmol), diphenyl sulfide (3.72 g, 20 mmol), silver tetrafluoroborate (1.94 g, 10 mmol) in $CHCl_3$(20 mL) after

chromatography (gradient from 2-5% MeOH in DCM) afforded (2-(4-nitrophenyl)-2-oxoethyl)diphenylsulfonium tetrafluoroborate as an orange solid (2.82 g, 65%). NaH (0.32 g, 8 mmol, 60 wt% in mineral oil) and (2-oxobutyl) diphenyl sulfonium tetrafluoroborate (1.75 g, 4 mmol) in anhydrous THF (25 mL), afforded 1-(diphenyl-λ⁴-sulfaneylidene)butan-2-one (**2g**) as a yellow oil (0.293 g, 21%).

^{1}H NMR (400 MHz, $CDCl_3$): δ 8.19 (d, *J* = 9.0 Hz, 2H), 8.04 (d, *J* = 9.0 Hz, 2H), 7.64-7.51 (m, 10H), 4.87 (s, 1H); **$^{13}C\{^1H\}$ NMR (101 MHz, $CDCl_3$):** δ 60.4, 123.4, 127.3, 128.4, 130.4, 131.6, 133.7, 146.6, 148.6, 178.9.

1-(diphenyl-λ^4-sulfaneylidene)butan-2-one (2q)

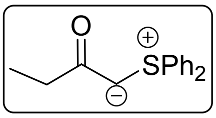

Following the General Procedure B with 1-bromobutan-2-one (1.51 g, 10 mmol), diphenyl sulfide (3.72 g, 20 mmol), silver tetrafluoroborate (1.94 g, 10 mmol) in $CHCl_3$(20 mL) after chromatography (gradient from 2-4% MeOH in DCM) afforded (2-oxobutyl)diphenylsulfonium tetrafluoroborate as yellow oil (2.58 g, 75%). NaH (0.32g, 8 mmol, 60 wt% in mineral oil) and (2-oxobutyl)diphenylsulfonium tetrafluoroborate (1.37 g, 4 mmol) in anhydrous THF (25 mL), afforded 1-(diphenyl-λ^4-sulfaneylidene)butan-2-one (**2q**) as a sticky yellow oil (1.01 g, 99%).

^{1}H NMR (400 MHz, $CDCl_3$): δ 7.55-7.44 (m, 10H), 4.08 (s, 1H), 2.29 (q, *J* = 7.5 Hz, 2H), 1.17 (t, *J* = 7.5 Hz, 3H); **$^{13}C\{^1H\}$ NMR (101 MHz, $CDCl_3$):** δ 191.7, 135.3, 131.4, 130.1, 128.7, 55.7, 34.3, 11.8; **FTIR: ν_{max}/cm^{-1} (neat):** 3054, 1783, 1264, 895, 732, 704; **HRMS (ESI$^+$) m/z:** [M + H]+ Calcd for $C_{16}H_{17}OS$: 257.1000; Found: 257.1000.

Cycloaddition Reactions

(4-methylene-1-tosylpyrrolidin-2-yl)(phenyl)methanone (3a)

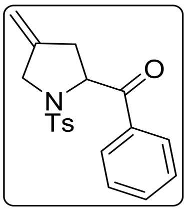

Following the General Procedure C with 2-(dimethyl-λ^4-sulfaneylidene)-1-phenylethan-1-one (**2a**) (54 mg, 0.3 mmol), 5-methylene-3-tosyl-1,3-oxazinan-2-one (**1a**) (27 mg, 0.1 mmol), *N,N*-diisopropyldibenzo[d,f][1,3,2]dioxaphosphepin-6-amine (1.7 mg, 0.0055 mmol), [(η^3-C_3H_5)PdCl]$_2$ (0.98 mg, 0.0025 mol) in DCM (1 mL) after chromatography (10% EtOAc in 40-60 petroleum ether) afforded (4-methylene-1-tosylpyrrolidin-2-yl)(phenyl)methanone (**3a**) as a white solid (21 mg, 75%).

^{1}H NMR (400 MHz, CDCl$_3$): δ 7.96 (d, *J* = 8.0 Hz, 2H), 7.72 (d, *J* = 8.0 Hz, 2H), 7.63 – 7.57 (m, 1H), 7.52 – 7.46 (m, 2H), 7.29 (d, *J* = 7.5 Hz, 2H), 5.50 (dd, *J* = 9.5, 3.0 Hz, 1H), 5.00 – 4.96 (m, 1H), 4.96 – 4.91 (m, 1H), 4.15 (d, *J* = 13.5 Hz, 1H), 4.06 (d, *J* = 13.5 Hz, 1H), 2.92 (dd, *J* = 15.5, 9.5 Hz, 1H), 2.59 (dd, *J* = 15.5, 1.0 Hz, 1H), 2.42 (s, 3H); **^{13}C{^{1}H} NMR (101 MHz, CDCl$_3$):** δ 196.8, 144.1, 142.4, 136.1, 134.7, 133.9, 129.9,129.1, 129.0, 127.9, 109.1 63.3, 52.3, 37.1, 21.9; **FTIR: v_{max}/cm^{-1} (neat):** 3061, 2923, 2851, 1662, 1596, 1447, 1329, 1218, 1159, 1094, 1015, 664, 558; **HRMS (ESI$^+$) m/z:** [M + H]+ Calcd for $C_{19}H_{20}NO_3S$: 342.1165; Found: 342.1168.

(4-methylene-1-((4-nitrophenyl)sulfonyl)pyrrolidin-2-yl)(phenyl)methanone (3b)

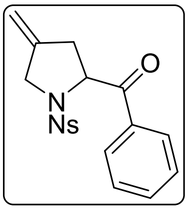

Following the General Procedure C with 2-(dimethyl-λ^4-sulfaneylidene)-1-phenylethan-1-one (**2a**) (27 mg, 0.15 mmol), 5-methylene-3-((4-nitrophenyl)sulfonyl)-1,3-oxazinan-2-one (**1b**) (30 mg, 0.1 mmol), *N,N*-diisopropyldibenzo[d,f][1,3,2]dioxaphosphepin-6-amine (1.7 mg, 0.0055 mmol), [(η^3-C_3H_5)PdCl]$_2$ (0.98 mg, 0.0025 mol) in DCM (1 mL) after chromatography (10% EtOAc in 40-60 petroleum ether) afforded (4-methylene-1-((4-nitrophenyl)sulfonyl)pyrrolidin-2-yl)(phenyl)methanone **(3b)** as a yellow solid (23 mg, 61%).

^{1}H NMR (400 MHz, CDCl$_3$): δ 8.34 (d, *J* = 9.0 Hz, 2H), 7.99 (d, *J* = 9.0 Hz, 2H), 7.89 (d, *J* = 7.5 Hz, 2H), 7.67 – 7.59 (m, 1H), 7.55 – 7.45 (m, 2H), 5.68 (dd, *J* = 9.5, 2.5 Hz, 1H), 5.07 – 5.00 (m, 1H), 5.00 – 4.96 (m, 1H), 4.30 (d, *J* = 13.0 Hz, 1H), 4.05 (d, *J* = 13.0 Hz, 1H), 3.13 (dd, *J* = 14.5, 11.0 Hz, 1H), 2.61 (d, *J* = 15.5 Hz, 1H); **^{13}C{^{1}H} NMR (101 MHz, CDCl$_3$):** δ 196.3, 150.2, 145.1, 141.6, 134.4, 134.2, 129.3, 129.0, 128.9, 124.5, 110.0, 63.9, 52.2, 37.6; **FTIR: v_{max}/cm^{-1} (neat):** 3061, 2919, 2851, 1695,

1529, 1350, 1216, 1166, 1100, 738, 617; **HRMS (ESI$^+$) m/z:** [M + H]+ Calcd for $C_{18}H_{17}N_2O_5S$: 373.0858; Found: 373.0860.

(4-methylene-1-(methylsulfonyl)pyrrolidin-2-yl)(phenyl)methanone (3c)

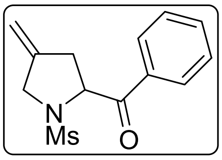

Following the General Procedure C with 2-(dimethyl-λ^4-sulfaneylidene)-1-phenylethan-1-one (**2a**) (27 mg, 0.15 mmol), 5-methylene-3-(methylsulfonyl)-1,3-oxazinan-2-one (**1c**) (19 mg, 0.1 mmol), *N,N*-diisopropyldibenzo[d,f][1,3,2]dioxaphosphepin-6-amine (1.7 mg, 0.0055 mmol), [(η^3-C_3H_5)PdCl]$_2$ (0.98 mg, 0.0025 mol) in DCM (1 mL) after chromatography (35% Et_2O in 40-60 petroleum ether) afforded (4-methylene-1-(methylsulfonyl)pyrrolidin-2-yl)(phenyl)methanone **(3c)** as a yellow oil (16 mg, 61%).

^{1}H NMR (400 MHz, CDCl$_3$): δ 8.01 – 7.86 (m, 2H), 7.68 – 7.57 (m, 1H), 7.53 – 7.45 (m, 2H), 5.63 (dd, *J* = 10.0, 2.5 Hz, 1H), 5.10 – 5.03 (m, 1H), 5.04 – 4.96 (m, 1H), 4.33 – 4.23 (m, 1H), 4.18 – 4.07 (m, 1H), 3.19 (dd, *J* = 15.0, 10.5 Hz, 1H), 3.03 (s, 3H), 2.63 (d, *J* = 15.0 Hz, 1H); **^{13}C{^{1}H} NMR (101 MHz, CDCl$_3$):** δ 197.6, 142.3, 134.3, 134.3, 129.3, 129.0, 109.4, 64.0, 51.9, 39.8, 37.6; **FTIR: ν_{max}/cm^{-1} (neat):** 3105, 2926 1694, 1528, 1349, 1215, 1165, 1101, 738, 616; **HRMS (ESI$^+$) m/z:** [M + Na]+ Calcd for $C_{13}H_{15}NO_3SNa$: 288.0670; Found: 288.0666.

(4-methylene-1-tosylpyrrolidin-2-yl)(p-tolyl)methanone (4)

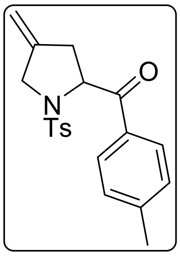

Following the General Procedure C with 2-(dimethyl-λ^4-sulfaneylidene)-1-(*p*-tolyl)ethan-1-one (**2b**) (29 mg, 0.15 mmol), 5-methylene-3-tosyl-1,3-oxazinan-2-one (**1a**) (27 mg, 0.1 mmol), *N,N*-diisopropyldibenzo[d,f][1,3,2]dioxaphosphepin-6-amine (1.7 mg, 0.0055 mmol), [(η^3-C_3H_5)PdCl]$_2$ (0.98 mg, 0.0025 mol) in DCM (1 mL) after chromatography (7.5% EtOAc in 40-60 petroleum ether) afforded (4-methylene-1-tosylpyrrolidin-2-yl)(p-tolyl)methanone (**4**) as a white solid (21 mg, 75%).

^{1}H NMR (400 MHz, CDCl$_3$): δ 7.88 (d, *J* = 8.0 Hz, 2H), 7.74 (d, *J* = 8.0 Hz, 2H), 7.36 – 7.25 (m, 4H), 5.51 (dd, *J* = 9.5, 3.0 Hz, 1H), 5.00-4.98 (m, 1H), 4.98 – 4.92 (m, 1H), 4.17 (d, *J* = 13.5 Hz, 1H), 4.09 (d, *J* = 13.5 Hz, 1H), 2.93 (dd, *J* = 15.5, 9.5 Hz, 1H), 2.59 (d, *J* = 15.5 Hz, 1H), 2.44 (s, 6H); **^{13}C{^{1}H} NMR (101 MHz, CDCl$_3$)**: δ 196.4, 144.9, 143.9, 142.8, 136.1, 132.2, 129.9, 129.8, 129.1, 127.9,

109.0, 63.2, 52.3, 37.3, 22.1, 21.9; **FTIR: ν_{max}/cm^{-1} (neat):** 2920, 2851, 1690, 1606, 1343, 1158, 1097, 664, 588, 547 ; **HRMS (ESI$^+$) m/z:** [M + H]+ Calcd for $C_{20}H_{22}NO_3S$: 356.1320; Found: 356.1315.

(4-methoxyphenyl)(4-methylene-1-tosylpyrrolidin-2-yl)methanone (5)

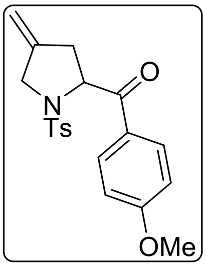

Following the General Procedure C with 2-(dimethyl-λ4-sulfaneylidene)-1-(4-methoxyphenyl)ethan-1-one (**2c**) (32 mg, 0.15 mmol), 5-methylene-3-tosyl-1,3-oxazinan-2-one (**1a**) (27 mg, 0.1 mmol), *N,N*-diisopropyldibenzo[d,f][1,3,2]dioxaphosphepin-6-amine (1.7 mg, 0.0055 mmol), $[(\eta^3\text{-}C_3H_5)PdCl]_2$ (0.98 mg, 0.0025 mol) in DCM (1 mL) after chromatography (10% EtOAc in 40-60 petroleum ether) afforded (4-methoxyphenyl)(4-methylene-1-tosylpyrrolidin-2-yl)methanone (**5**) as a white solid (26 mg, 70%).

^{1}H NMR (400 MHz, CDCl$_3$): δ 7.98 (d, *J* = 9.0 Hz, 2H), 7.74 (d, *J* = 8.0 Hz, 2H), 7.31 (d, *J* = 8.0 Hz, 2H), 6.97 (d, *J* = 9.0 Hz, 2H), 5.48 (dd, *J* = 9.5, 3.0 Hz, 1H), 5.00-4.98 (m, 1H), 4.96 – 4.92 (m, 1H), 4.17 (d, *J* = 13.5 Hz, 1H), 4.08 (d, *J* = 13.5 Hz, 1H), 3.90 (s, 3H), 2.90 (dd, *J* = 15.5, 9.5 Hz, 1H), 2.60 (d, *J* = 15.5 Hz, 1H), 2.44 (s, 3H); **^{13}C{^{1}H} NMR (101 MHz, CDCl$_3$):** δ 195.3, 164.2, 143.9, 142.9, 136.0, 131.4, 129.9, 127.9, 127.8, 114.3, 108.8, 62.9, 55.9, 52.3, 37.3, 21.9; **FTIR: ν_{max}/cm^{-1} (neat):** 2920, 2851, 1600, 1161, 1260, 1097, 666, 588 ; **HRMS (ESI$^+$) m/z:** [M + H]+ Calcd for $C_{20}H_{22}NO_4S$: 372.1270; Found: 372.1274.

(4-methylene-1-tosylpyrrolidin-2-yl)(4-(trifluoromethyl)phenyl)methanone (6)

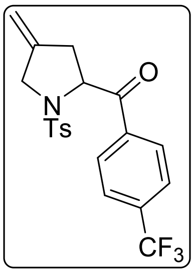

Following the General Procedure C with 2-(dimethyl-λ^4-sulfaneylidene)-1-(4-(trifluoromethyl)phenyl)ethan-1-one (**2d**) (74 mg, 0.3 mmol), 5-methylene-3-tosyl-1,3-oxazinan-2-one (**1a**) (27 mg, 0.1 mmol), *N,N*-diisopropyldibenzo[d,f][1,3,2]dioxaphosphepin-6-amine (1.7 mg, 0.0055 mmol), $[(\eta^3\text{-}C_3H_5)PdCl]_2$ (0.98 mg, 0.0025 mol) in DCM (1 mL) after chromatography (7.5% EtOAc in 40-60 petroleum ether) afforded (4-methylene-1-tosylpyrrolidin-2-yl)(4-(trifluoromethyl)phenyl)methanone(**6**) as a yellow solid (32 mg, 79%).

^{1}H NMR (400 MHz, CDCl$_3$): δ 8.21 – 8.05 (m, 2H), 7.77 (d, *J* = 8.0 Hz, 2H), 7.74 (d, *J* = 8.0 Hz, 2H), 7.33 (d, *J* = 8.0 Hz, 2H), 5.41 (dd, J = 9.5, 3.5 Hz, 1H), 5.03 (s, 1H), 4.99 (s, 1H), 4.14 (d, *J* = 14.0 Hz, 1H), 4.08 (d, *J* = 14.0 Hz, 1H), 2.91 (dd, J = 16.0, 9.5 Hz, 1H), 2.71 – 2.56 (m, 1H), 2.46 (s, 3H); **^{19}F**

NMR (377 MHz, $CDCl_3$): δ -63.2; **$^{13}C\{^1H\}$ NMR (101 MHz, $CDCl_3$):** δ 196.3, 144.3, 142.2, 137.6, 135.4, 135.1 (q, *J* = 33.0 Hz), 130.1, 129.5, 128.0, 126.2, 123.8 (q, *J* = 272.5 Hz), 109.5, 63.8, 52.4, 36.7, 21.9; **FTIR: ν_{max}/cm^{-1} (neat):** 2917, 2850, 1702, 1326, 1161, 1067, 589; **HRMS (ESI^+) m/z:** [M + H]+ Calcd for $C_{20}H_{19}F_3NO_3S$: 410.1038; Found: 410.1044.

(4-chlorophenyl)(4-methylene-1-tosylpyrrolidin-2-yl)methanone (7)

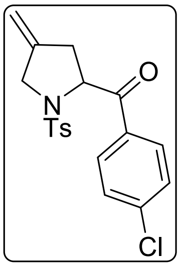

Following the General Procedure C with 1-(4-chlorophenyl)-2-(dimethyl-λ^4-sulfaneylidene)ethan-1-one (**2e**) (32 mg, 0.15 mmol), 5-methylene-3-tosyl-1,3-oxazinan-2-one (**1a**) (27 mg, 0.1 mmol), *N,N*-diisopropyldibenzo[d,f][1,3,2]dioxaphosphepin-6-amine (1.7 mg, 0.0055 mmol), $[(\eta^3\text{-}C_3H_5)PdCl]_2$ (0.98 mg, 0.0025 mol) in DCM (1 mL) after chromatography (7.5% EtOAc in 40-60 petroleum ether) afforded (4-chlorophenyl)(4-methylene-1-tosylpyrrolidin-2-yl)methanone (**7**) as a yellow solid (32 mg, 79%).

^1H NMR (400 MHz, $CDCl_3$): δ 7.95 (d, *J* = 7.0 Hz, 2H), 7.73 (d, *J* = 7.0 Hz, 2H), 7.48 (d, *J* = 7.0 Hz, 2H), 7.32 (d, *J* =7.0 Hz, 2H), 5.40 (d, *J* = 7.0 Hz, 1H), 5.00 (s, 1H), 4.96 (s, 1H), 4.13 (d, *J* = 13.0 Hz, 1H), 4.07 (d, *J* = 13.0 Hz, 1H), 2.99 – 2.79 (m, 1H), 2.61 (d, *J* = 15.5 Hz, 1H), 2.45 (s, 3H); **$^{13}C\{^1H\}$ NMR (101 MHz, $CDCl_3$):** δ 195.8, 144.1, 142.4, 140.5, 135.6, 133.1, 130.5, 130.0, 129.5, 127.9, 109.2, 63.4, 52.3, 36.9, 21.9; **FTIR: ν_{max}/cm^{-1} (neat):** 2920, 2851, 1695, 1589, 1343, 1158, 1092, 663, 558; **HRMS (ESI^+) m/z:** [M + H]+ Calcd for $C_{19}H_{19}{}^{35}ClNO_3S$: 376.0774; Found: 376.0784.

(4-fluorophenyl)(4-methylene-1-tosylpyrrolidin-2-yl)methanone (8)

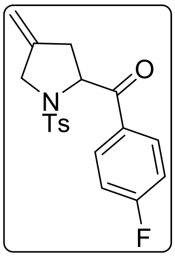

Following the General Procedure C with 2-(dimethyl-λ^4-sulfaneylidene)-1-(4-fluorophenyl)ethan-1-one (**2f**) (30 mg, 0.15 mmol), 5-methylene-3-tosyl-1,3-oxazinan-2-one (**1a**) (27 mg, 0.1 mmol), *N,N*-diisopropyldibenzo[d,f][1,3,2]dioxaphosphepin-6-amine (1.7 mg, 0.0055 mmol), $[(\eta^3\text{-}C_3H_5)PdCl]_2$ (0.98 mg, 0.0025 mol) in DCM (1 mL) after chromatography (7.5% EtOAc in 40-60 petroleum ether) afforded (4-fluorophenyl)(4-methylene-1-tosylpyrrolidin-2-yl)methanone (**8**) as a yellow solid (22 mg, 60%).

^1H NMR (400 MHz, $CDCl_3$): δ 8.02 (dd, *J* = 8.5, 5.5 Hz, 2H), 7.71 (d, *J* = 8.0 Hz, 2H), 7.30 (d, *J* = 8.0 Hz, 2H), 7.18-7.13 (m, 2H), 5.41 (dd, *J* = 9.5, 3.0 Hz, 1H), 4.98 (s, 1H), 4.94 (s, 1H), 4.12 (d, *J* = 14.0

Hz, 1H), 4.05 (d, J = 14.0 Hz, 1H), 2.93 – 2.82 (m, 1H), 2.59 (d, J = 15.5 Hz, 1H), 2.43 (s, 3H); **^{19}F NMR (376 MHz, $CDCl_3$):** δ -103.9 – -104.0 (m); **$^{13}C\{^1H\}$ NMR (101 MHz, $CDCl_3$)**: δ 195.5, 167.6, 165.1, 144.1, 142.6, 135.7, 131.8, 130.0, 128.0, 116.3 (d, J = 22.0 Hz), 109.2, 63.3, 52.3, 37.0, 21.9. **FTIR: ν_{max}/cm^{-1} (neat):** 2919, 2851, 1701, 1588, 1345, 1260, 1161, 750, 651, 589**; HRMS (ESI$^+$) m/z:** [M + H]+ Calcd for $C_{19}H_{19}FNO_3S$: 360.1070; Found: 360.1067.

4-methylene-2-(4-nitrophenyl)-1-tosylpyrrolidine (9)

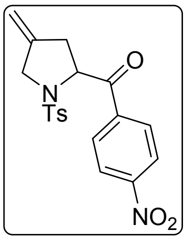

Following the General Procedure D with 2-(diphenyl-l^4-sulfaneylidene)-1-(4-nitrophenyl)ethan-1-one (**2l**) (70 mg, 0.2 mmol), 5-methylene-3-tosyl-1,3-oxazinan-2-one (**1a**) (27 mg, 0.1 mmol), *N,N*-diisopropyldibenzo[d,f][1,3,2]dioxaphosphepin-6-amine (3.5 mg, 0.011 mmol), $[(\eta^3\text{-}C_3H_5)PdCl]_2$ (1.83 mg, 0.005 mol) in DCM (1 mL) at 50 °C after chromatography (30% Et_2O in 40-60 petroleum ether) afforded 4-methylene-2-(4-nitrophenyl)-1-tosylpyrrolidine (**9**) as a yellow oil (17 mg, 45%).

^{1}H NMR (400 MHz, $CDCl_3$)：δ 8.35 (d, J = 8.5 Hz, 2H), 8.20 (d, J = 8.5 Hz, 2H), 7.74 (d, J = 8.0 Hz, 2H), 7.35 (d, J = 8.0 Hz, 2H), 5.32 (dd, J = 9.5, 4.0 Hz, 1H), 5.04 (s, 1H), 5.00 (s, 1H), 4.09 (br, 2H), 2.89 (dd, J = 16.0, 9.5 Hz, 1H), 2.67 (d, J = 16.0 Hz, 1H), 2.46 (s, 3H); **$^{13}C\{^1H\}$ NMR (101 MHz, $CDCl_3$)**: δ 196.1, 150.9, 144.4, 141.9, 139.3, 134.9, 130.2 (x2C), 128.0, 124.3, 109.8, 64.1, 52.4, 36.4, 21.9; **FTIR: ν_{max}/cm^{-1} (neat):** 2921, 2856, 1703, 1525, 1346, 1160, 1096, 665, 588, 548**; HRMS (ESI$^+$) m/z:** [M + Na]+ Calcd for $C_{19}H_{18}N_2O_5SNa$: 409.0834; Found: 409.0836.

4-(4-methylene-1-tosylpyrrolidine-2-carbonyl)benzonitrile (10)

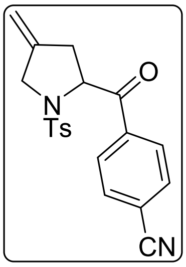

Following the General Procedure C with 2-(dimethyl-λ^4-sulfaneylidene)-1-(4-fluorophenyl)ethan-1-one (**2g**) (31 mg, 0.15 mmol), 5-methylene-3-tosyl-1,3-oxazinan-2-one (**1a**) (27 mg, 0.1 mmol), *N,N*-diisopropyldibenzo[d,f][1,3,2]dioxaphosphepin-6-amine (1.7 mg, 0.0055 mmol), $[(\eta^3\text{-}C_3H_5)PdCl]_2$ (0.98 mg, 0.0025 mol) in DCM (1 mL) after chromatography (20% Et_2O in 40-60 petroleum ether) afforded 4-(4-methylene-1-tosylpyrrolidine-2-carbonyl)benzonitrile (**10**) as a yellow oil (10 mg, 27%). A clean ^{13}C NMR spectrum could not be obtained and so this compound was characterized by ^{1}H NMR, FTIR and

mass spectroscopy only.

^{1}H NMR (400 MHz, $CDCl_3$): δ 8.21 (d, *J* = 8.5 Hz, 2H), 7.90 (d, *J* = 8.5 Hz, 2H), 7.82 (d, *J* = 8.0 Hz, 2H), 7.43 (d, *J* = 8.0 Hz, 2H), 5.41 (dd, *J* = 9.5, 4.0 Hz, 1H), 5.16 – 5.10 (m, 1H), 5.11 – 5.05 (m, 1H), 4.21-4.15 (m, 2H), 2.97 (dd, *J* = 16.0, 9.5 Hz, 1H), 2.73 (dd, *J* = 16.0, 1.5 Hz, 1H), 2.55 (s, 3H); **FTIR: ν_{max}/cm^{-1} (neat):** 2929, 2232, 1705, 1160, 734, 664, 589**; HRMS (ESI$^+$) m/z:** [M + Na]+ Calcd for $C_{20}H_{18}N_2O_3SNa$: 389.0936; Found: 389.0935.

(2-methoxyphenyl)(4-methylene-1-tosylpyrrolidin-2-yl)methanone (11)

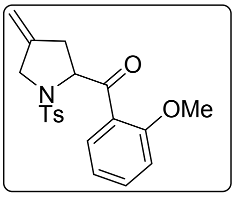

Following the General Procedure C with 2-(dimethyl-λ^4-sulfaneylidene)-1-(2-methoxyphenyl)ethan-1-one (32 mg, 0.15 mmol), 5-methylene-3-tosyl-1,3-oxazinan-2-one (**1a**) (27 mg, 0.1 mmol), *N,N*-diisopropyldibenzo[d,f][1,3,2]dioxaphosphepin-6-amine (1.7 mg, 0.0055 mmol), [(η^3-C_3H_5)PdCl]$_2$ (0.98 mg, 0.0025 mol) in DCM (1 mL) after chromatography (gradient from 25-30% Et_2O in 40-60 petroleum ether) afforded (**11**) (2-methoxyphenyl)(4-methylene-1-tosylpyrrolidin-2-yl)methanone as a yellow oil (22 mg, 26%).

^{1}H NMR (400 MHz, $CDCl_3$): δ 7.71 (d, *J* = 8.0 Hz, 2H), 7.57 – 7.45 (m, 2H), 7.28 (d, *J* = 8.0 Hz, 2H), 7.06 – 6.92 (m, 2H), 5.59 (dd, *J* = 9.5, 2.0 Hz, 1H), 5.02 – 4.92 (m, 1H), 4.92 – 4.84 (m, 1H), 4.18 (d, *J* = 14.0 Hz, 1H), 4.03 (d, *J* = 14.0 Hz, 1H), 3.93 (s, 3H), 2.90 (dd, *J* = 15.0, 10.5 Hz, 1H), 2.55 (d, *J* = 15.0 Hz, 1H), 2.42 (s, 3H); **^{13}C{^{1}H} NMR (101 MHz, $CDCl_3$):** δ 198.4, 159.2, 143.6, 143.3, 136.7, 134.8, 131.7, 129.8, 127.8, 125.5, 121.39, 112.1, 108.6, 67.01, 56.0, 52.6, 37.0, 21.9; **FTIR: ν_{max}/cm^{-1} (neat):** 2924, 2851, 1681, 1598, 1286, 1160, 1097, 666, 588; **HRMS (ESI$^+$) m/z**: [M + H]+ Calcd for $C_{20}H_{22}NO_4S$: 372.1270; Found: 372.1274.

(3-methoxyphenyl)(4-methylene-1-tosylpyrrolidin-2-yl)methanone (12)

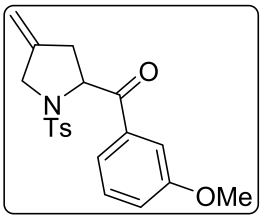

Following the General Procedure C with 2-(dimethyl-λ^4-sulfaneylidene)-1-(3-methoxyphenyl)ethan-1-one (**2h**) (32 mg, 0.15 mmol), 5-methylene-3-tosyl-1,3-oxazinan-2-one (**1a**) (27 mg, 0.1 mmol), *N,N*-diisopropyldibenzo[d,f][1,3,2]dioxaphosphepin-6-amine (1.7 mg, 0.0055 mmol), [(η^3-C_3H_5)PdCl]$_2$ (0.98 mg, 0.0025 mol) in DCM (1 mL) after chromatography (gradient from 25-30% Et_2O in 40-60 petroleum ether) afforded (**12**) (3-methoxyphenyl)(4-methylene-1-

tosylpyrrolidin-2-yl)methanone as a yellow oil (22 mg, 60%).

^{1}H NMR (400 MHz, CDCl$_3$): δ 7.72 (d, *J* = 8.0 Hz, 2H), 7.54 (d, *J* = 8.0 Hz, 1H), 7.48-7.47 (m, 1H), 7.41-7.17 (m, 1H), 7.30 (d, *J* = 8.0 Hz, 2H), 7.14 (dd, *J* = 8.0, 2.0 Hz, 1H), 5.48 (dd, *J* = 9.5, 3.0 Hz, 1H), 4.98 (s, 1H), 4.93 (s, 1H), 4.15 (d, *J* = 13.5 Hz, 1H), 4.06 (d, *J* = 13.5 Hz, 1H), 3.85 (s, 3H), 2.96 – 2.83 (m, 1H), 2.59 (d, *J* = 15.5 Hz, 1H), 2.43 (s, 3H); **^{13}C{^{1}H} NMR (101 MHz, CDCl$_3$)**: δ 196.7, 160.2, 143.9, 142.6, 136.1, (x2C), 130.0, 129.9, 127.9, 121.4, 120. 5, 113.3, 109.0, 63.4, 55.8, 52.5, 37.2, 21.9; **FTIR: ν_{max}/cm^{-1} (neat):** 2920, 2851, 1701, 1600, 1260,1161, 1097, 666, 588**; HRMS (ESI$^+$) m/z:** [M + H]+ Calcd for $C_{20}H_{22}NO_4S$: 372.1270; Found: 372.1274.

(3-fluorophenyl)(4-methylene-1-tosylpyrrolidin-2-yl)methanone (13)

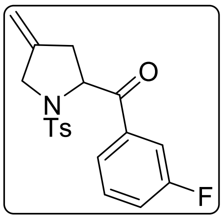

Following the General Procedure C with 2-(dimethyl-λ^4-sulfaneylidene)-1-(3-fluorophenyl)ethan-1-one (**2i**) (30 mg, 0.15 mmol), 5-methylene-3-tosyl-1,3-oxazinan-2-one (**1a**) (27 mg, 0.1 mmol), *N,N*-diisopropyldibenzo[d,f][1,3,2]dioxaphosphepin-6-amine (1.7 mg, 0.0055 mmol), [(η^3-C_3H_5)PdCl]$_2$ (0.98 mg, 0.0025 mol) in DCM (1 mL) after chromatography (30% Et_2O in 40-60 petroleum ether) afforded **(**3-fluorophenyl)(4-methylene-1-tosylpyrrolidin-2-yl)methanone (**13**) as a yellow oil (23 mg, 64%).

^{1}H NMR (400 MHz, CDCl$_3$): δ 7.79 (d, *J* = 7.5 Hz, 1H), 7.74 (d, *J* = 8.0 Hz, 2H), 7.66 (d, *J* = 9.0 Hz, 1H), 7.50 (td, *J* = 8.0, 5.5 Hz, 1H), 7.34-7.31 (m, 3H), 5.42 (dd, *J* = 9.5, 3.0 Hz, 1H), 5.01 (s, 1H), 4.97 (s, 1H), 4.15 (d, *J* = 13.0 Hz, 1H), 4.08 (d, *J* = 13.0 Hz, 1H), 2.93 (dd, *J* = 15.0, 10.5 Hz, 1H), 2.61 (d, *J* = 15.0 Hz, 1H), 2.45 (s, 3H)**; ^{19}F NMR (376 MHz, CDCl$_3$):** δ -111.06 – 111.12 (m)**; ^{13}C{^{1}H} NMR (101 MHz, CDCl$_3$):** δ 195.7, 164.4, 161.8, 144.2, 142.4, 135.6, 130.9 (d, *J* = 7.5 Hz), 130.0, 127.9, 124.8 (d, *J* = 3.0 Hz), 121.0 (d, *J* = 21.5 Hz), 115.8 (d, *J* = 22.5 Hz), 109.4, 63.4, 52.2, 36.7, 21.9; **FTIR: ν_{max}/cm^{-1} (neat):** 2919, 2851, 1701, 1588, 1345, 1260, 1161, 750, 651, 589**; HRMS (ESI$^+$) m/z:** [M + H]+ Calcd for $C_{19}H_{19}FNO_3S$: 360.1070; Found: 360.1067

(4-methylene-1-tosylpyrrolidin-2-yl)(naphthalen-1-yl)methanone (14)

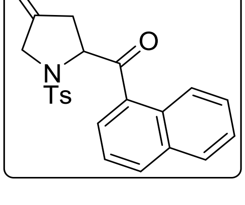

Following the General Procedure C with 2-(dimethyl-λ^4-sulfaneylidene)-1-(naphthalen-2-yl)ethan-1-one (**2j**) (30 mg, 0.15 mmol), 5-methylene-3-

tosyl-1,3-oxazinan-2-one (**1a**) (27 mg, 0.1 mmol), *N,N*-diisopropyldibenzo[d,f][1,3,2]dioxaphosphepin-6-amine (1.7 mg, 0.0055 mmol), $[(\eta^3\text{-}C_3H_5)PdCl]_2$ (0.98 mg, 0.0025 mol) in DCM (1 mL) after chromatography (10% EtOAc in 40-60 petroleum ether) afforded (4-methylene-1-tosylpyrrolidin-2-yl)(naphthalen-1-yl)methanone (**14**) as a yellow oil (23 mg, 57%).

^{1}H NMR (400 MHz, $CDCl_3$): δ 8.52 (s, 1H), 7.98 (d, *J* = 8.5 Hz, 2H), 7.94 – 7.87 (m, 2H), 7.74 (d, *J* = 8.0 Hz, 2H), 7.60 (dt, *J* = 15.0, 7.0 Hz, 2H), 7.29 (d, *J* = 8.0 Hz, 2H), 5.68 (dd, *J* = 9.5, 3.0 Hz, 1H), 5.00 (s, 1H), 4.95 (s, 1H), 4.21 (d, *J* = 13.5 Hz, 1H), 4.11 (d, *J* = 13.5 Hz, 1H), 2.99 (dd, *J* = 16.0, 9.5 Hz, 1H), 2.66 (d, *J* = 16.0 Hz, 1H), 2.42 (s, 3H); **^{13}C{^{1}H} NMR (101 MHz, $CDCl_3$):** δ 196.8, 143.8, 142.8, 136.1, 132.8, 132.1, 130.9, 130.0 (x2C), 129.9, 129.2, 129.1, 128.1, 127.9, 127.3, 124.4, 108.9, 63.4, 52.3, 37.4, 21.9; **FTIR: ν_{max}/cm^{-1} (neat):** 2919, 2851, 1690, 1597, 1345, 1277, 1160, 763, 665, 589**; HRMS (ESI$^+$) m/z:** [M + H]+ Calcd for $C_{23}H_{22}NO_3S$: 392.1320; Found: 392.1317.

(4-methylene-1-tosylpyrrolidin-2-yl)(thiophen-2-yl)methanone (15)

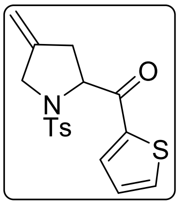

Following the General Procedure C with 2-(dimethyl-λ^4-sulfaneylidene)-1-(thiophen-2-yl)ethan-1-one (**2k**) (26 mg, 0.3 mmol), 5-methylene-3-tosyl-1,3-oxazinan-2-one (**1a**) (27 mg, 0.1 mmol), *N,N*-diisopropyldibenzo[d,f][1,3,2]dioxaphosphepin-6-amine (1.7 mg, 0.0055 mmol), $[(\eta^3\text{-}C_3H_5)PdCl]_2$ (0.98 mg, 0.0025 mol) in DCM (1 mL) after chromatography (10% EtOAc in 40-60 petroleum ether) afforded (4-methylene-1-tosylpyrrolidin-2-yl)(thiophen-2-yl)methanone (**15**) as a yellow oil (21 mg, 60%).

^{1}H NMR (400 MHz, $CDCl_3$): δ 7.93 (d, *J* = 3.5 Hz, 1H), 7.73 – 7.69 (m, 3H), 7.30 (d, *J* = 8.0 Hz, 2H), 7.17 (t, *J* = 4.5 Hz, 1H), 5.11 (dd, *J* = 9.0, 4.0 Hz, 1H), 4.98 (s, 1H), 4.95 (s, 1H), 4.09 (s, 2H), 2.84 (dd, *J* = 15.5, 9.5 Hz, 1H), 2.68 (d, *J* = 15.5 Hz, 1H), 2.43 (s, 3H); **^{13}C{^{1}H} NMR (101 MHz, $CDCl_3$):** δ 190.2, 144.2, 142.4, 140.9, 135.1, 135.06, 133.8, 130.0, 128.6, 128.1, 109.3, 64.4, 52.8, 37.3, 22.4; **FTIR: ν_{max}/cm^{-1} (neat):** 3092, 2924, 1668, 1413, 1343, 1158, 1095, 665, 588, 548**; HRMS (ESI$^+$) m/z:** [M + H]+ Calcd for $C_{17}H_{28}NO_3S_2$: 348.0728; Found: 348.0722.

2,2-dimethyl-1-(4-methylene-1-tosylpyrrolidin-2-yl)propan-1-one (16)

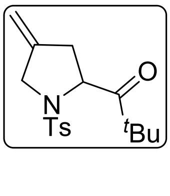

Following the General Procedure C with 2-(dimethyl-λ^4-sulfaneylidene)-1-

(thiophen-2-yl)ethan-1-one (**2l**) (24 mg, 0.15 mmol), 5-methylene-3-tosyl-1,3-oxazinan-2-one (**1a**) (27 mg, 0.1 mmol), *N,N*-diisopropyldibenzo[d,f][1,3,2]dioxaphosphepin-6-amine (1.7 mg, 0.0055 mmol), [(η^3-C_3H_5)PdCl]$_2$ (0.98 mg, 0.0025 mol) in DCM (1 mL) after chromatography (7.5% EtOAc in 40-60 petroleum ether) afforded 2,2-dimethyl-1-(4-methylene-1-tosylpyrrolidin-2-yl)propan-1-one (**16**) as a yellow oil (6 mg, 18%). A clean ^{13}C NMR spectrum could not be obtained and so this compound was characterized by ^{1}H NMR, FTIR and mass spectroscopy only.

^{1}H NMR (400 MHz, CDCl$_3$): δ 7.70 (d, *J* = 8.0 Hz, 2H), 7.30 (d, *J* = 8.0 Hz, 2H), 5.04 (dd, *J* = 9.5, 3.5 Hz, 1H), 4.98 – 4.94 (m, 1H), 4.94 – 4.90 (m, 1H), 4.10 (d, *J* = 13.5 Hz, 1H), 4.04 (d, *J* = 13.5 Hz, 1H), 2.76 (dd, *J* = 15.5, 9.5 Hz, 1H), 2.42 (s, 3H), 2.34 (d, *J* = 15.5 Hz, 1H), 1.20 (s, 9H); **FTIR: ν_{max}/cm^{-1} (neat):** 3054, 2969, 1714, 1528, 1348, 1164, 1100, 1061, 736, 618**; HRMS (ESI$^+$) m/z:** [M + Na]+ Calcd for $C_{17}H_{23}NO_3SNa$: 344.1296; Found: 344.1298.

ethyl 4-methylene-1-tosylpyrrolidine-2-carboxylate (17)

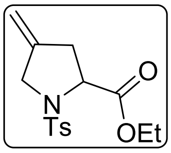

Following the General Procedure D with ethyl 2-(diphenyl-λ^4-sulfaneylidene)acetate (**2k**) (54 mg, 0.2 mmol), 5-methylene-3-tosyl-1,3-oxazinan-2-one (**1a**) (27 mg, 0.1 mmol), *N,N*-diisopropyldibenzo[d,f][1,3,2]dioxaphosphepin-6-amine (3.5 mg, 0.011 mmol), [(η^3-C_3H_5)PdCl]$_2$ (1.83 mg, 0.005 mol) in DCM (1 mL) after chromatography (20% Et_2O in 40-60 petroleum ether) afforded ethyl 4-methylene-1-tosylpyrrolidine-2-carboxylate (**17**) as a yellow oil (20 mg, 65%).

^{1}H NMR (400 MHz, CDCl$_3$): δ 7.74 (d, *J* = 8.0 Hz, 2H), 7.31 (d, *J* = 8.0 Hz, 2H), 4.98 – 4.97 (m, 1H), 4.96 – 4.95 (m, 1H), 4.45 (dd, *J* = 9.0, 3.5 Hz, 1H), 4.19 – 4.04 (m, 2H), 4.02 (s, 2H), 2.77 (dd, *J* = 15.5, 9.0 Hz, 1H), 2.61 (d, *J* = 15.5 Hz, 1H), 2.42 (s, 3H), 1.22 (t, *J* = 7.0 Hz, 3H); **^{13}C{^{1}H} NMR (101 MHz, CDCl$_3$):** δ 171.7, 144.0, 142.5, 135.7, 130.0, 128.0, 108.7, 61.6, 60.9, 52.1, 37.4, 22.0, 14.4; **FTIR: ν_{max}/cm^{-1} (neat):** 2920, 2850, 1742, 1463, 1350, 1163, 1097, 665, 589, 549**; HRMS (ESI$^+$) m/z:** [M + H]+ Calcd for $C_{15}H_{20}NO_4S$: 310.1113; Found: 310.1113.

1-(4-methylene-1-tosylpyrrolidin-2-yl)propan-1-one (18)

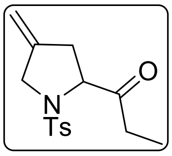

Following the General Procedure D with 1-(diphenyl-λ^4-sulfaneylidene)butan-2-one (**2m**) (51 mg, 0.2 mmol), 5-methylene-3-tosyl-1,3-oxazinan-2-one (**1a**) (27 mg, 0.1 mmol), *N,N*-diisopropyldibenzo[d,f][1,3,2]dioxaphosphepin-6-amine

(3.5 mg, 0.011 mmol), [(η^3-C_3H_5)PdCl]$_2$ (1.83 mg, 0.005 mol) in DCM (1 mL) after chromatography (60% Et_2O in 40-60 petroleum ether) afforded 1-(4-methylene-1-tosylpyrrolidin-2-yl)propan-1-one (**18**) as a yellow oil (17 mg, 55%).

^{1}H NMR (400 MHz, CDCl$_3$) : δ 7.71 (d, *J* = 8.0 Hz, 2H), 7.33 (d, *J* = 8.0 Hz, 2H), 4.94 – 4.93 (m, 1H), 4.93 – 4.92 (m, 1H), 4.20 (dd, *J* = 9.5, 4.0 Hz, 1H), 4.04 (d, *J* = 14.0 Hz, 1H), 3.89 (d, *J* = 14.0 Hz, 1H), 2.95-2.84 (m, 1H), 2.66 – 2.39 (m, 6H), 1.07 (t, *J* = 7.0 Hz, 3H); **^{13}C{^{1}H} NMR (101 MHz, CDCl$_3$)**: δ 210.6, 144.5, 142.2, 134.0, 130.2, 128.0, 109.1, 67.3, 52.9, 35.4, 31.9, 21.9, 7.6; **FTIR: ν_{max}/cm^{-1} (neat):** 3054, 2993, 1722, 1264, 1162, 1097, 896, 733, 704; **HRMS (ESI$^+$) m/z:** [M + H]+ Calcd for $C_{15}H_{20}NO_3S$: 294.1164; Found: 294.1165.

(*S*)-3-(2-(diphenyl-λ^4-sulfaneylidene)acetyl)-4-isopropyloxazolidin-2-one (19)

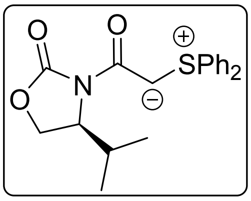

Following the General Procedure B with (*S*)-3-(2-bromoacetyl)-4-isopropyloxazolidin-2-one (2.50 g, 10 mmol), diphenyl sulfide (3.72 g, 20 mmol), silver tetrafluoroborate (1.94 g, 10 mmol) in $CHCl_3$ (20 mL) after chromatography (2% MeOH in DCM) afforded (*S*)-(2-(4isopropyl-2-oxooxazolidin-3-yl)-2-oxoethyl)diphenylsulfonium tetrafluoroborate as yellow solid (3.41 g, 77%). NaH (0.32 g, 8 mmol, 60 wt% in mineral oil) and (2-ethoxy-2-oxoethyl) diphenyl sulfonium tetrafluoroborate (1.77 g, 4 mmol) in anhydrous THF (25 mL), afforded (*S*)-3-(2-(diphenyl-λ^4-sulfaneylidene)acetyl)-4-isopropyloxazolidin-2-one (**19**) as a sticky orange oil (1.40 g, 99%).

^{1}H NMR (400 MHz, CDCl$_3$): δ 7.61-7.40 (m, 10H), 5.19 (s, 1H), 4.52 (dt, *J* = 8.5. 3.5 Hz, 1H), 4.21 (dd, *J* = 9.0, 3.0 Hz, 1H), 4.13 (dd, *J* = 9.0, 3.0 Hz, 1H), 2.50 – 2.36 (m, 1H), 0.91 (d, *J* = 6.5 Hz, 3H), 0.89 (d, *J* = 6.5 Hz, 3H); **^{13}C{^{1}H} NMR (101 MHz, CDCl$_3$):** δ 163.8, 155.6, 135.5, 131.4, 130.2, 128.8, 63.3, 58.9, 29.7, 18.4, 15.2; **FTIR: ν_{max}/cm^{-1} (neat):** 2962, 1748, 1596, 1573, 1350, 1195, 1065, 745, 689; **HRMS (ESI$^+$) m/z:** [M + H]+ Calcd for $C_{20}H_{23}NO_3S$: 356.1320; Found: 356.1328.

(*S*)-4-isopropyl-3-((*R*)-4-methylene-1-tosylpyrrolidine-2-carbonyl)oxazolidin-2-one (20)

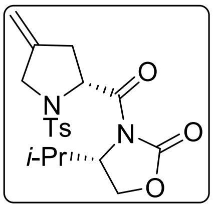

Following the General Procedure D with (*S*)-3-(2-(diphenyl-λ^4-sulfaneylidene)acetyl)-4-isopropyloxazolidin-2-one (**2n**) (71 mg, 0.2 mmol), 5-methylene-3-tosyl-1,3-oxazinan-2-one (**1a**) (27 mg, 0.1 mmol), *N,N*-diisopropyldibenzo[d,f][1,3,2]dioxaphosphepin-6-amine (3.5 mg, 0.011

mmol), [(η^3-C_3H_5)PdCl]$_2$ (1.83 mg, 0.005 mol) in DCM (1 mL) after chromatography (40% Et_2O in 40-60 petroleum ether) afforded (*S*)-4-isopropyl-3-((*R*)-4-methylene-1-tosylpyrrolidine-2-carbonyl)oxazolidin-2-one (**20**) as a yellow oil (25 mg, 65%; >18:1 dr).

^{1}H NMR (400 MHz, CDCl$_3$): δ 7.77 (d, *J* = 8.0 Hz, 2H), 7.34 (d, *J* = 8.0 Hz, 2H), 5.67 (dd, *J* = 9.0, 3.5 Hz, 1H), 4.99 – 4.98 (m, 1H), 4.97 – 4.96 (m, 1H), 4.49 (dt, *J* = 6.5. 3.5 Hz, 1H), 4.21 (dd, *J* = 9.0, 3.0 Hz, 1H), 4.28 (dd, *J* = 9.0, 3.0 Hz, 1H), 4.15 (d, *J* = 14.0 Hz, 1H), 3.98 (d, *J* = 14.0 Hz, 1H), 2.95 (dd, *J* = 17.0, 10.0 Hz, 1H), 2.52 (d, *J* = 15.0 Hz, 1H), 2.45 (s, 3H), 2.39 – 2.31 (m, 1H), 0.92 (d, *J* = 7.0 Hz, 3H), 0.88 (d, *J* = 7.0 Hz, 3H); **^{13}C{^{1}H} NMR (101 MHz, CDCl$_3$):** δ 171.2, 154.4, 143.9, 142.3, 136.0, 130.1, 127.7, 109.1, 64.7, 60.9, 58.7, 52.3, 38.1, 28.9, 21.9, 18.1, 15.2; **FTIR: ν_{max}/cm^{-1} (neat):**2962, 2922, 1776, 1713, 1389, 1343, 1160, 1096, 815**; HRMS (ESI$^+$) m/z:** [M + H]+ Calcd for $C_{19}H_{25}N_2O_5S$: 393.1484; Found: 393.1489.

Gram scale synthesis of compound 17

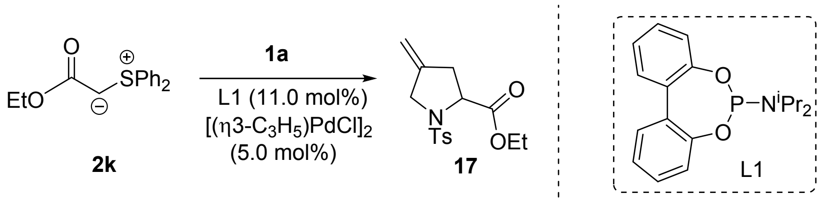

A flame-dried 50 mL round bottomed flask was charged 5-methylene-3-tosyl-1,3-oxazinan-2-one **1a** (1.34 g, 5.0 mmol), *N,N*-diisopropyldibenzo[d,f][1,3,2]dioxaphosphepin-6-amine **L1** (0.081 g, 0.275 mmol) and [(η^3-C_3H_5)PdCl]$_2$ (0.041 g 0.125 mmol) under nitrogen. Anhydrous DCM (5 mL) was then added, and the mixture stirred at rt for 15 minutes. Ethyl 2-(diphenyl-λ^4-sulfaneylidene)acetate (2.04 g, 7.5 mmol) in DCM (5 mL) was added and the reaction mixture was stirred overnight at rt. A further portion of *N,N*-diisopropyldibenzo[d,f][1,3,2]dioxaphosphepin-6-amine **L1** (0.081 g, 0.275 mmol), [(η^3-C_3H_5)PdCl]$_2$ (0.041 g 0.125 mmol) and ethyl 2-(diphenyl-λ4-sulfaneylidene)acetate (0.68 g, 2.5 mmol) were added and the reaction mixture was stirred for another 2 h at rt. The resulting mixture was then concentrated under vacuum and purified by flash column chromatography (20% Et_2O in 40-60 petroleum ether) to afford ethyl 4-methylene-1-tosylpyrrolidine-2-carboxylate (**17**) as a yellow oil (1.24 g, 80%).

Functionalization Reactions

ethyl 4-oxo-1-tosylpyrrolidine-2-carboxylate (21)

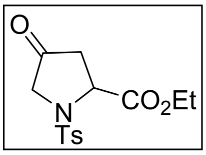

To a solution of ethyl 4-methylene-1-tosylpyrrolidine-2-carboxylate (**17**) (31 mg, 0.1 mmol) dissolved in MeCN/DCM/H_2O (1:1:2 v/v/v, 2.0 mL) was added $RuCl_3$ (6.2 mg, 0.03 mmol) and the reaction mixture was stirred at 0 °C for 30 mins. Then NaIO4 (171 mg, 0.8 mmol) was added, and the reaction mixture was stirred at room temperate. After 4 hours, the resulting mixture was extracted with DCM and the combined organic portions were washed with brine and dried with $MgSO_4$. The mixture was evaporated under high vacuum and purified by column chromatography (50% Et_2O in 40-60 petroleum ether) to afford ethyl 4-oxo-1-tosylpyrrolidine-2-carboxylate (**21**) (19 mg, 61%) as a white solid.

^{1}H NMR (400 MHz, $CDCl_3$): δ 7.73 (d, *J* = 8.0 Hz, 2H), 7.34 (d, *J* = 8.0 Hz, 2H), 4.76 (dd, *J* = 9.5, 2.5 Hz, 1H), 4.11 – 3.99 (m, 2H), 3.84 (d, *J* = 17.4 Hz, 1H), 3.77 (d, *J* = 17.5 Hz, 1H)2.77 (dd, *J* = 18.0, 9.5 Hz, 1H), 2.55 (dd, *J* = 18.0, 1.5 Hz, 1H), 2.44 (s, 3H), 1.19 (t, *J* = 7.1 Hz, 3H); **$^{13}C\{^1H\}$ NMR (101 MHz, $CDCl_3$):** δ 206.7, 170.8, 145.0, 135.0, 130.3, 128.1, 62.2, 57.9, 52.9, 41.9, 21.9, 14.2; **FTIR: ν_{max}/cm^{-1} (neat):** 2987, 2923, 1768, 1743, 1350, 1264, 729, 712; **HRMS (ESI$^+$) m/z:** [M + Na]+ Calcd for $C_{14}H_{17}NO_5SNa$: 334.0725; Found: 334.0723.

ethyl 4-(propan-2-ylidene)-1-tosylpyrrolidine-2-carboxylate （22）

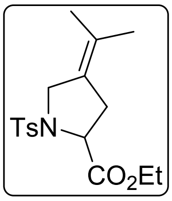

To a solution of ethyl 4-methylene-1-tosylpyrrolidine-2-carboxylate (**17**) (31 mg, 0.1 mmol) and 2-methyl-2-butene (0.5 mL) in DCE (1 mL) under nitrogen was added $Ti(O^iPr)_4$ (9.5 mg, 0.03 mmol). After 15 mins, the 2nd generation Hoveyda-Grubbs catalyst (7 mg, 0.01 mmol) was added and the resulting mixture was heated at 50 °C overnight. After cooling, the mixture was evaporated under vacuum and purified by column chromatography (30% Et_2O in 40-60 petroleum ether) to afford **e**thyl 4-(propan-2-ylidene)-1-tosylpyrrolidine-2-carboxylate (**22**) as a yellow oil (24 mg, 84%).

^{1}H NMR (400 MHz, $CDCl_3$): δ 7.74 (d, *J* = 8.0 Hz, 2H), 7.30 (d, *J* = 8.0 Hz, 2H), 4.40 (dd, *J* = 9.0, 4.0 Hz, 1H), 4.13-4.07 (m, 2H), 4.02 (d, *J* = 14.0 Hz, 1H), 3.93 (d, *J* = 14.0 Hz, 1H), 2.77 (dd, *J* = 15.5, 9.0 Hz, 1H), 2.66 (d, *J* = 15.5 Hz, 1H), 2.42 (s, 3H), 1.56 (s, 6H), 1.22 (t, *J* = 7.0 Hz, 3H); **$^{13}C\{^1H\}$ NMR (101 MHz, $CDCl_3$):** δ 172.0, 143.9, 135.6, 129.9, 127.9, 126.2, 125.5, 61.6, 61.1, 50.3, 34.6, 21.8, 21.3, 21.1, 14.3; **FTIR: ν_{max}/cm^{-1} (neat):** 2988, 2917, 1737, 1347, 1161, 1094, 1031, 815; **HRMS (ESI$^+$) m/z:** [M+H]+ Calcd for $C_{17}H_{24}NO_4S$: 338.1426; Found: 338.1425.

(4-methylene-1-tosylpyrrolidin-2-yl)methanol（23）

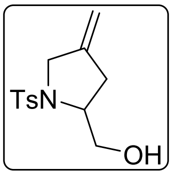

To a suspension of $LiAlH_4$ (4.5 mg, 0.12 mmol) in THF (1 mL) under nitrogen was added ethyl 4-methylene-1-tosylpyrrolidine-2-carboxylate (**17**) (31 mg, 0.1 mmol) in THF (1 mL) and the resulting mixture stirred at 0 °C for 1 hour. H_2O (0.6 mL) was then added dropwise and the reaction stirred for 15 minutes at 0 °C before the addition of anhydrous $MgSO_4$. After 15 minutes the reaction mixture was filtered through celite and the volatiles removed under vacuum. Purification by column chromatography (50% Et_2O in 40-60 petroleum ether) afforded (4-methylene-1-tosylpyrrolidin-2-yl)methanol (**23**) as a colourless oil (18 mg, 69%).

^{1}H NMR (400 MHz, $CDCl_3$): δ 7.73 (d, *J* = 7.5 Hz, 2H), 7.33 (d, *J* = 7.5 Hz, 2H), 4.94-4.88 (m, 2H), 4.03 (d, *J* = 14.5 Hz, 1H), 3.88 (d, *J* = 14.5 Hz, 1H), 3.83 – 3.63 (m, 1H), 3.66-3.57 (m, 2H), 2.57 (br, OH), 2.43 (s, 3H), 2.34-2.27 (m, 2H); **^{13}C{^{1}H} NMR (101 MHz, $CDCl_3$):** δ 144.1, 142.9, 134.2, 130.2, 127.9, 108.5, 65.3, 62.1, 53.4, 35.1, 21.9; **FTIR: v_{max}/cm^{-1} (neat):** 3061, 2989, 1264, 734, 704; **HRMS (ESI$^+$) m/z:** [M + H]+ Calcd for $C_{13}H_{18}NO_3S$: 268.1007; Found: 268.0999.

1-(*tert*-butyl) 2-methyl 4-methylenepyrrolidine-1,2-dicarboxylate (24)[7]

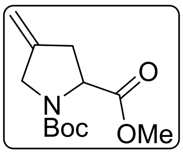

To a solution of ethyl 4-methylene-1-tosylpyrrolidine-2-carboxylate (**17**) (77 mg, 0.25 mmol) in MeOH (12.5 mL) under nitrogen was added magnesium turnings (425 mg, 17.50 mmol). The reaction mixture was ultrasonicated at 60 °C for 6 h. sat. NH_4Cl (4.6 ml) and water (4.6 mL) was added. The mixture was extracted with DCM, dried over $MgSO_4$ and the volatiles removed under high vacuum. To a solution of the crude product in DCM (1.25 mL) under nitrogen was added di-*tert*-butyl dicarbonate (109 mg, 0.50 mmol) portionwise, followed by 4-(dimethylamino) pyridine (3.1 mg, 0.025 mmol) and TEA (0.078 mL, 0.55 mmol) at 0 °C. The reaction mixture was slowly warmed to room temperature and stirred overnight. The resulting mixture was concentrated under vacuum and purified by column chromatography (20% Et_20 in 40-60 petroleum ether) to afford 1-1-(*tert*-butyl) 2-methyl 4-methylenepyrrolidine-1,2-dicarboxylate (**24**) as a colorless oil (9.5 mg, 16%; 5:4 mixture of rotamers). NMR data correlated with literature data.

^{1}H NMR (400 MHz, $CDCl_3$): δ 5.00 (d, *J* = 12.0 Hz, 2H), 4.50 (dd, *J* = 9.5, 2.5 Hz, .0.4H), 4.39 (dd, *J* =

9.5, 2.5 Hz, 0.5H), 4.07 (d, *J* = 15.0 Hz, 2H), 3.72 (s, 3H), 2.96 (dd, *J* = 26.0, 16.0 Hz, 1H), 2.62 (dd, *J* = 16.0, 4.0 Hz, 1H), 1.47 (s, 4H), 1.42 (s, 5H); **$^{13}C\{^1H\}$ NMR (101 MHz, $CDCl_3$):** δ 173.5, 143.8, 142.6, 108.4, 108.2, 80.5, 59.4, 58.9, 52.6, 52.4, 51.1, 50.9, 37.1, 36.4, 28.7, 28.6.

(*R*)-2-(((tert-butyldimethylsilyl)oxy)methyl)-4-methylene-1-tosylpyrrolidine

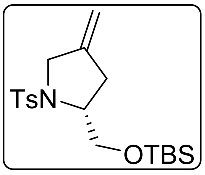

To a solution of (*R*)-(4-methylene-1-tosylpyrrolidin-2-yl)methanol (26.7 mg, 0.1 mmol) in DCM (2 mL) under nitrogen was added imidazole (7.5 mg, 0.11 mmol) and *tert*-butyldimethylsilyl chloride (16.6 mg, 0.11 mmol). The reaction mixture was stirred at room temperature overnight. The resulting mixture was quenched with NH_4Cl and extracted with EtOAc. The organic portion was washed with brine and dried with $MgSO_4$ and the solvent removed in vacuo. Purification by column chromatography (10% Et_2O in 40-60 petroleum ether) afforded (*R*)-2-(((tert-butyldimethylsilyl)oxy)methyl)-4-methylene-1-tosylpyrrolidine as a colorless oil (15 mg, 82%).

^1H NMR (400 MHz, $CDCl_3$): δ 7.71 (d, *J* = 8.0 Hz, 2H), 7.29 (d, *J* = 8.0 Hz, 2H), 4.89 (br, 2H), 3.94 – 3.80 (m, 3H), 3.76 (dd, *J* =10.0, 4.0 Hz, 1H), 3.45 (dd, *J* = 10.0, 8.5 Hz, 1H), 2.44 (d, *J* = 15.5 Hz, 1H), 2.41 (s, 3H), 2.20 (dd, *J* = 15.5, 8.5 Hz, 1H), 0.86 (s, 9H), 0.04 (s, 3H), 0.03 (s, 3H); **$^{13}C\{^1H\}$ NMR (101 MHz, $CDCl_3$):** δ 144.0, 143.8, 135.4, 130.0, 127.6, 108.2, 65.5, 61.2, 52.9, 34.7, 26.1, 21.8, 18.4, -5.27; **FTIR: ν_{max}/cm^{-1} (neat):** 3052, 2863, 1265, 733, 704; **HRMS (ESI^+) m/z:** [M + H]+ Calcd for $C_{19}H_{32}NO_3SSi$: 382.1872; Found: 382.1871.

tert-butyl(*R*)-2-(((*tert*-butyldimethylsilyl)oxy)methyl)-4-methylenepyrrolidine-1-carboxylate (25) [8]

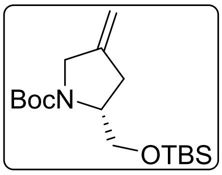

To a solution of (*R*)-2-(((*tert*-butyldimethylsilyl)oxy)methyl)-4-methylene-1-tosylpyrrolidine (152 mg, 0.40 mmol) in MeOH (20.0 mL) under nitrogen was added magnesium turnings (677 mg, 27.88 mmol). The reaction mixture was ultrasonicated at 60 °C for 6 h, and sat. NH_4Cl was added to quench the reaction. The mixture was extracted with DCM, dried over $MgSO_4$ and the volatiles removed under vacuum. To a solution of the crude product in DCM (2.0 mL) under nitrogen was added di-*tert*-butyl dicarbonate (175 mg, 0.80 mmol) portionwise, followed by 4-(dimethylamino)pyridine (4.8 mg, 0.04 mmol) and TEA (0.12 mL, 0.55 mmol) at 0 °C. The reaction mixture was slowly warmed to

room temperature and stirred overnight. The resulting mixture was concentrated under vacuum and purified by column chromatography (5% Et_20 in 40-60 petroleum ether) to afford *tert*-butyl (*R*)-2-(((*tert*-butyldimethylsilyl)oxy)methyl)-4-methylenepyrrolidine-1-carboxylate (**25**) as a colorless oil (52 mg, 40%; 3:2 mixture of rotamers).

^{1}H NMR (400 MHz, $CDCl_3$): δ 4.96-4.90 (m, 2H), 4.08-3.92 (m, 2H), 3.83 (br, 0.6H), 3.79 (br, 0.4H), 3.62 (dd, *J* = 9.5, 3.0 Hz, 1H), 3.61 – 3.29 (m, 1H), 2.72-2.63 (m, 1H), 2.56 (d, *J* = 15.0 Hz, 1H) 1.45 (s, 9H), 0.86 (s, 9H), 0.02 (s, 6H); **^{13}C{^{1}H} NMR (101 MHz, $CDCl_3$):** δ 154.4, 107.5, 106.6, 79.8, 79.5, 63.8, 63.6, 58.7, 58.4, 51.8, 51.1, 35.2, 34.6, 28.9, 26.2, 18.5, -4.9; **HPLC** (Cellulose-2, hexane: ^{i}PrOH 99.9:0.1, flow rate 1.0 mL/min, λ = 210 nm, 25 °C) t_R(major) = 11.960 min, t_R(minor) = 13.637 min, ee = 95%; ; $[\alpha]_D^{22} = +20$ (*c* 1.0, $CHCl_3$).

NMR Spectra

(4-methylene-1-tosylpyrrolidin-2-yl)(phenyl)methanone (3a)

^{1}H NMR, $CDCl_3$, 400 MHz

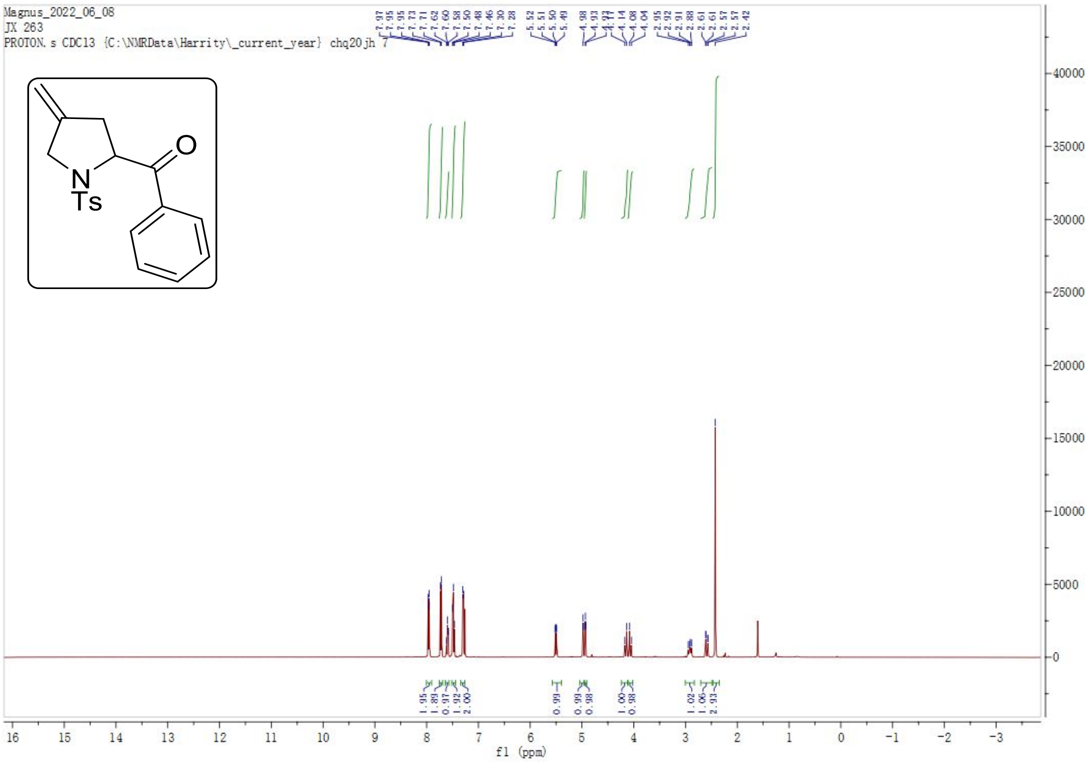

^{13}C{^{1}H} NMR, $CDCl_3$, 101 MHz

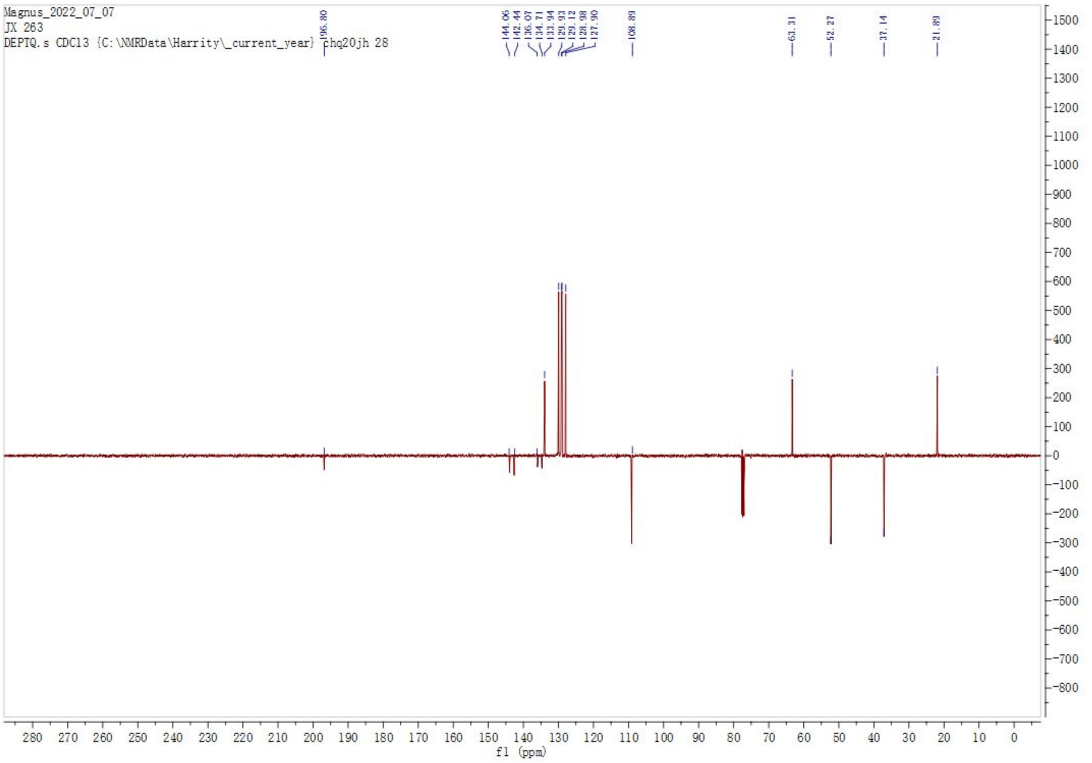

(4-methylene-1-((4-nitrophenyl)sulfonyl)pyrrolidin-2-yl)(phenyl)methanone (3b)

^{1}H NMR, $CDCl_3$, 400 MHz

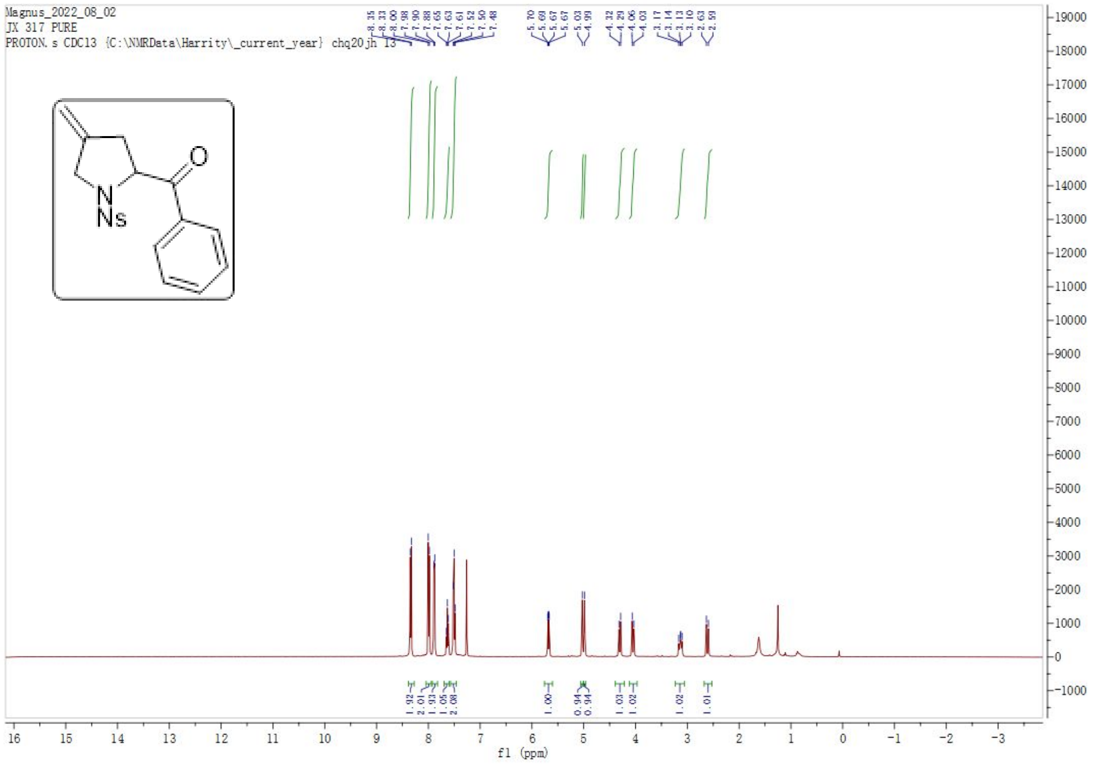

$^{13}C\{^{1}H\}$ NMR, $CDCl_3$, 101 MHz

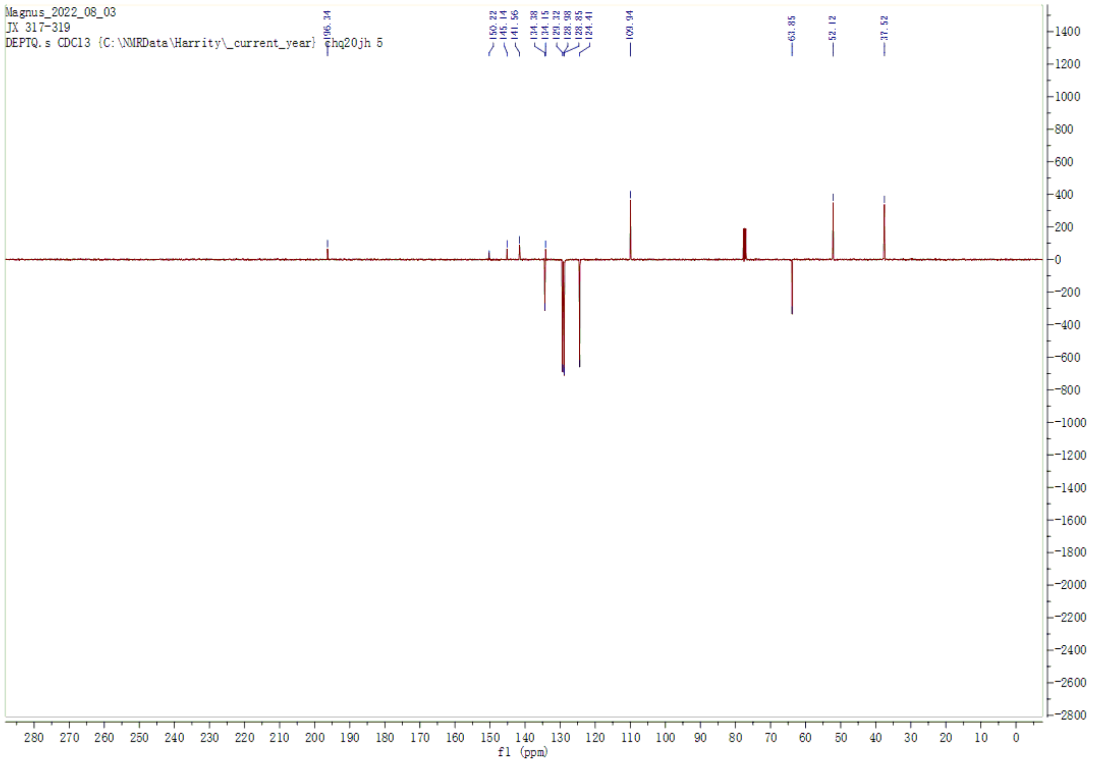

(4-methylene-1-(methylsulfonyl)pyrrolidin-2-yl)(phenyl)methanone (3c)

^{1}H NMR, $CDCl_3$, 400 MHz

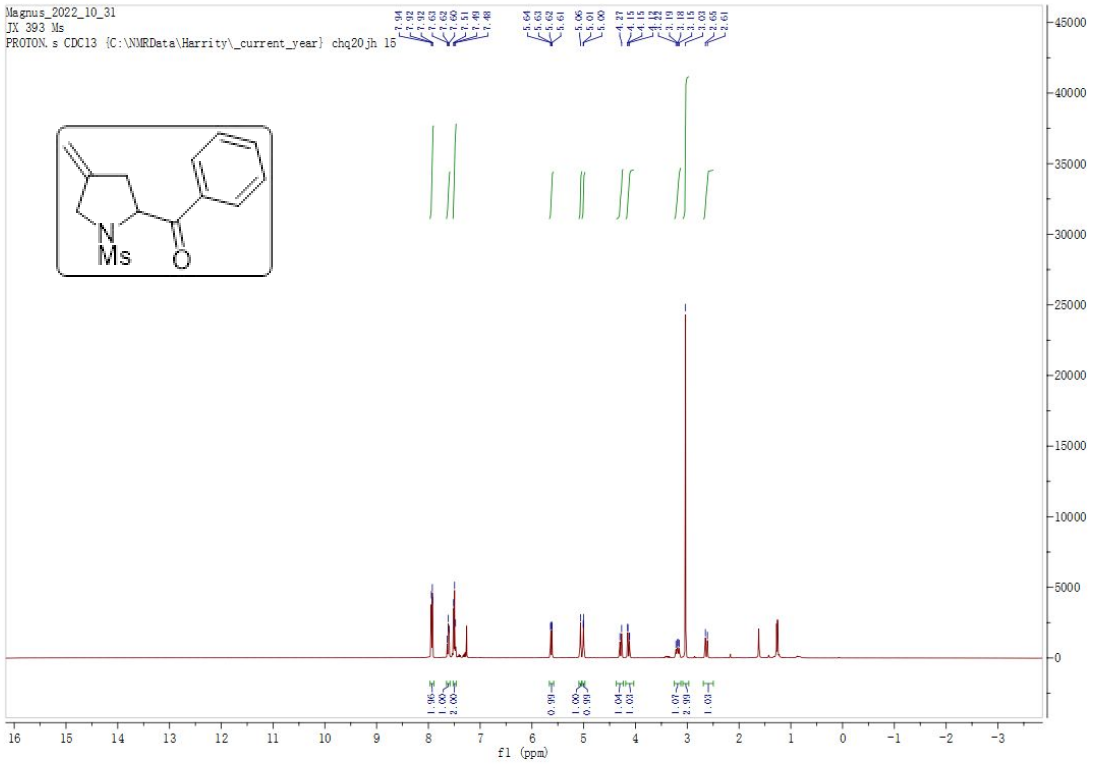

$^{13}C\{^{1}H\}$ NMR, $CDCl_3$, 101 MHz

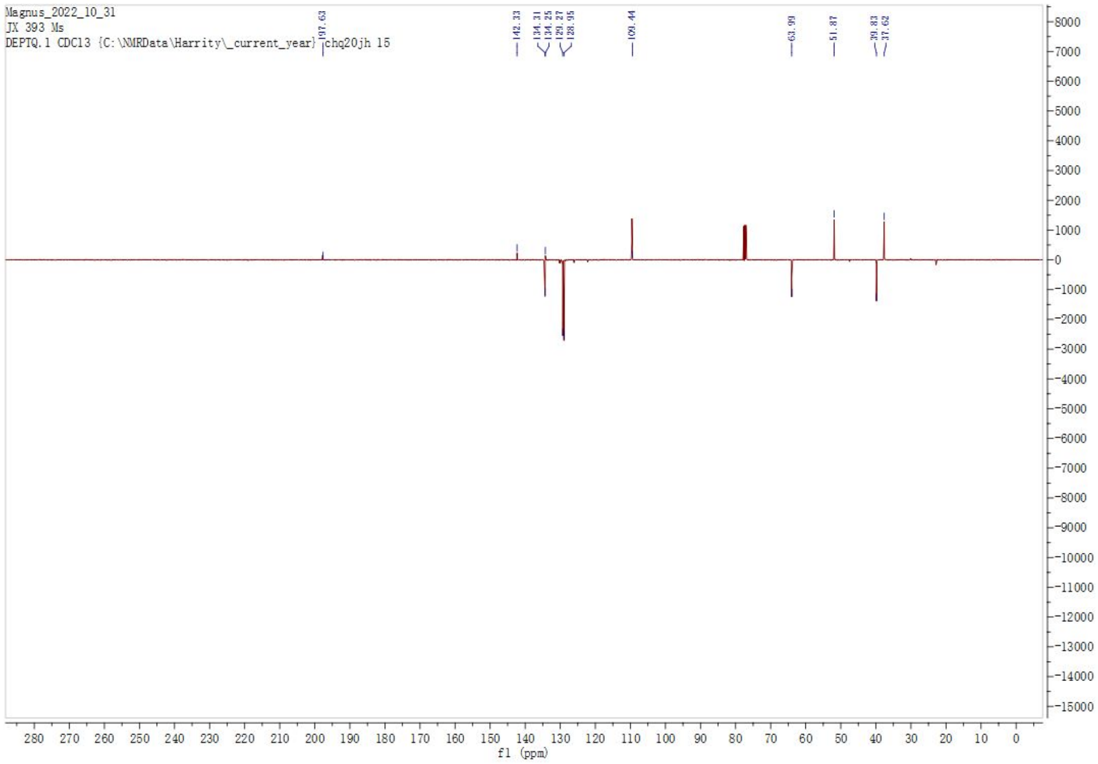

(4-methylene-1-tosylpyrrolidin-2-yl)(p-tolyl)methanone (4)

^{1}H NMR, $CDCl_3$, 400 MHz

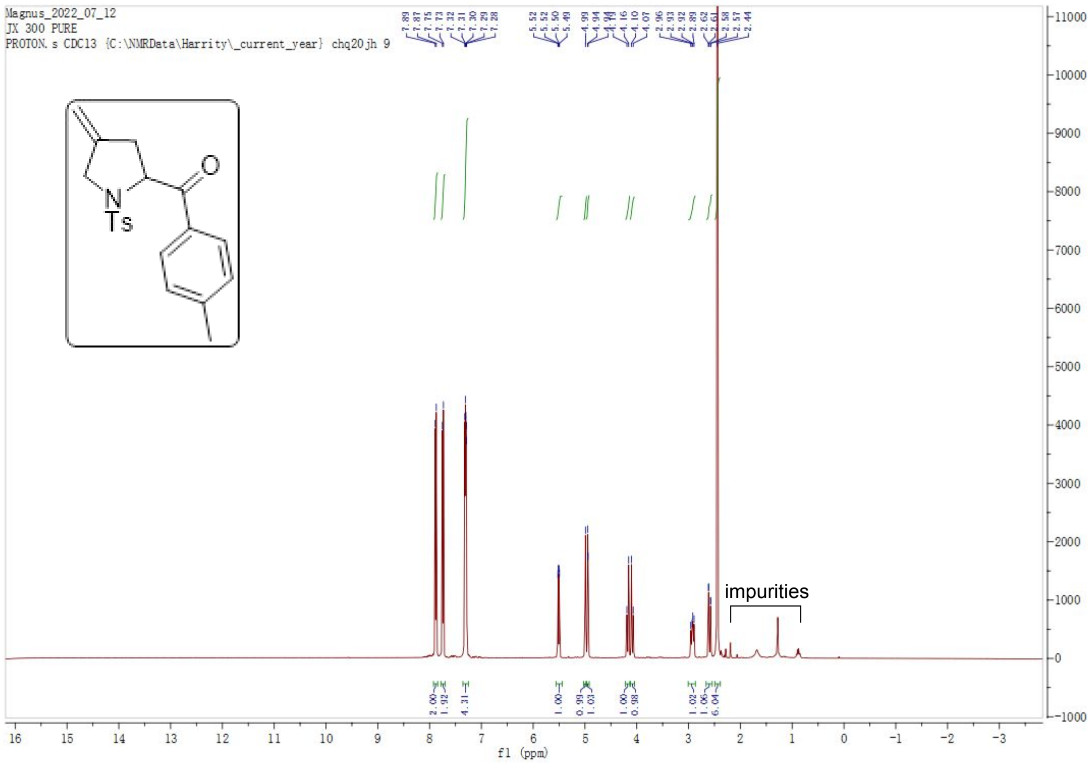

^{13}C{^{1}H} NMR, $CDCl_3$, 101 MHz

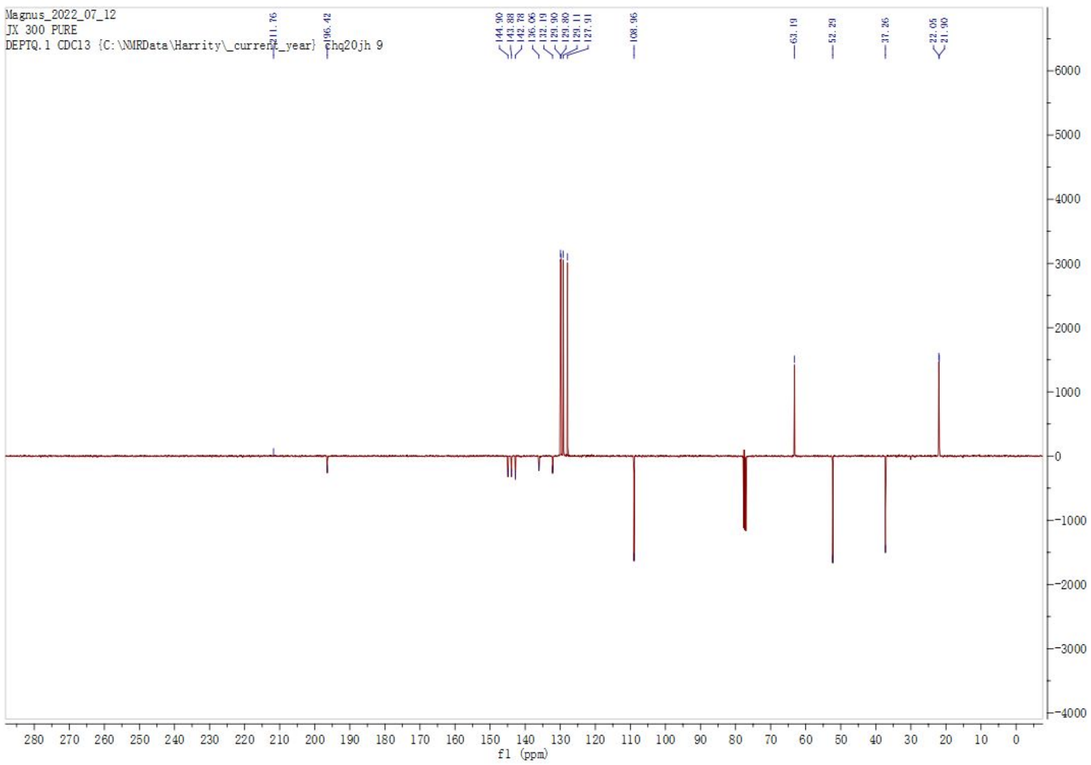

(4-methoxyphenyl)(4-methylene-1-tosylpyrrolidin-2-yl)methanone (5)

^{1}H NMR, $CDCl_3$, 400 MHz

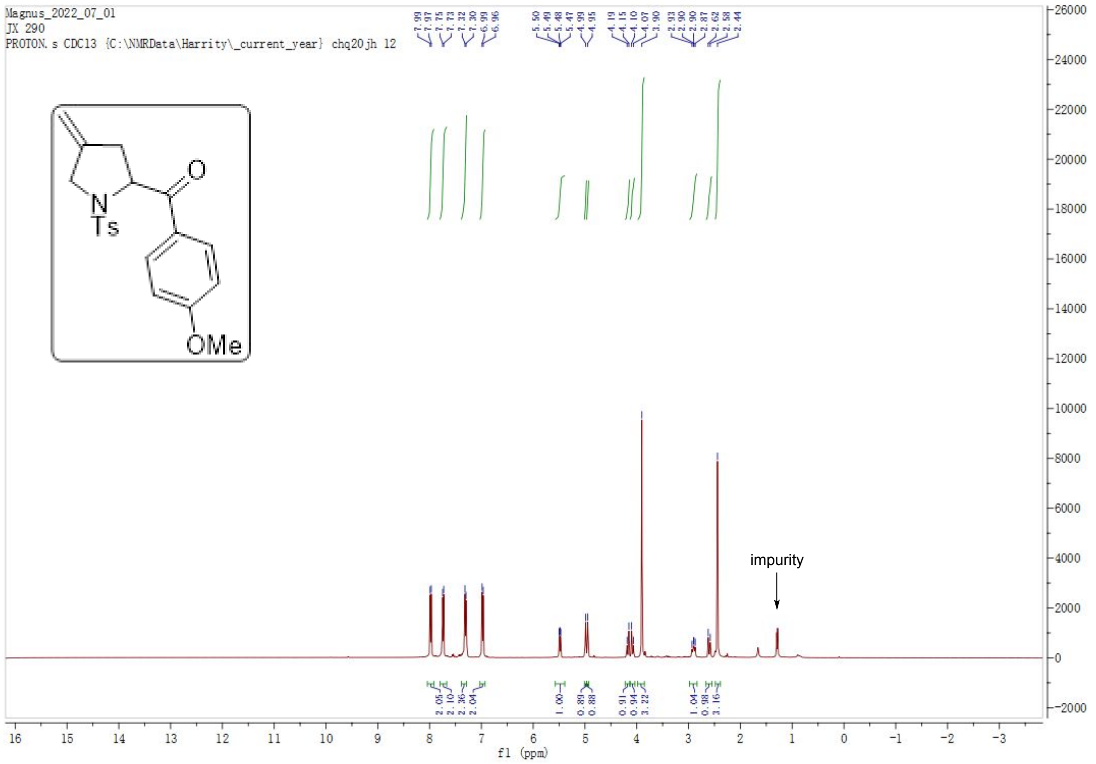

^{13}C{^{1}H} NMR, $CDCl_3$, 101 MHz

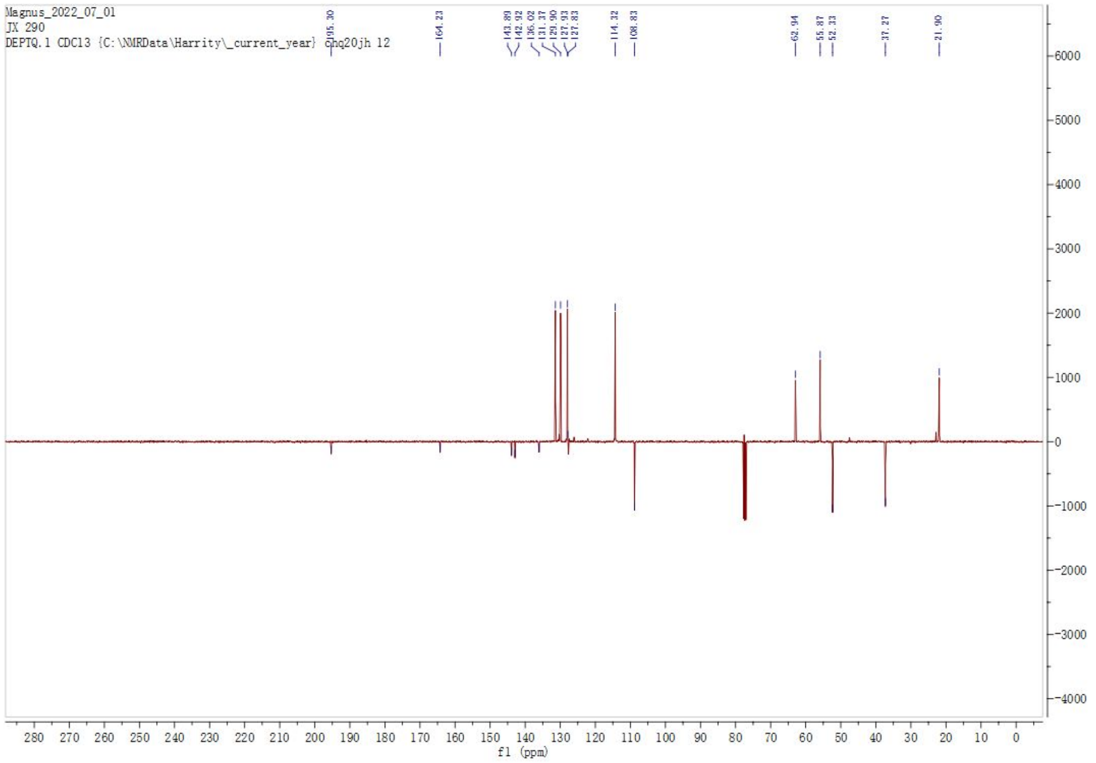

(4-methylene-1-tosylpyrrolidin-2-yl)(4-(trifluoromethyl)phenyl)methanone (6)

^{1}H NMR, $CDCl_3$, 400 MHz

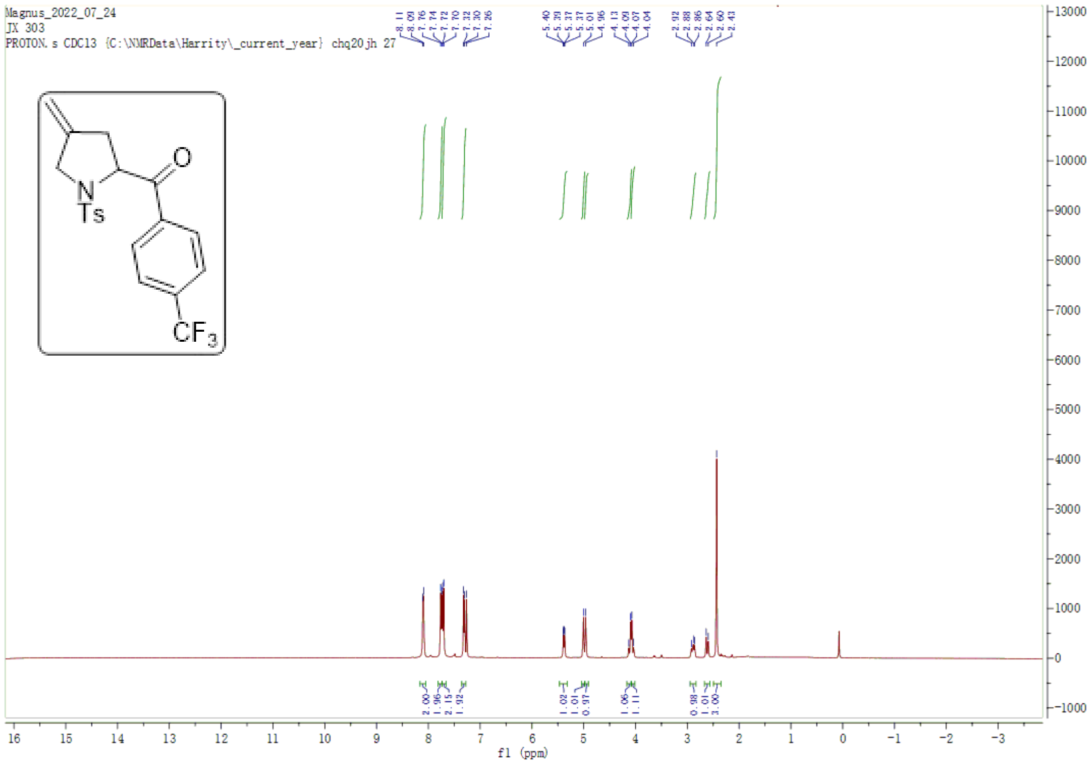

^{19}F NMR, $CDCl_3$, 377 MHz

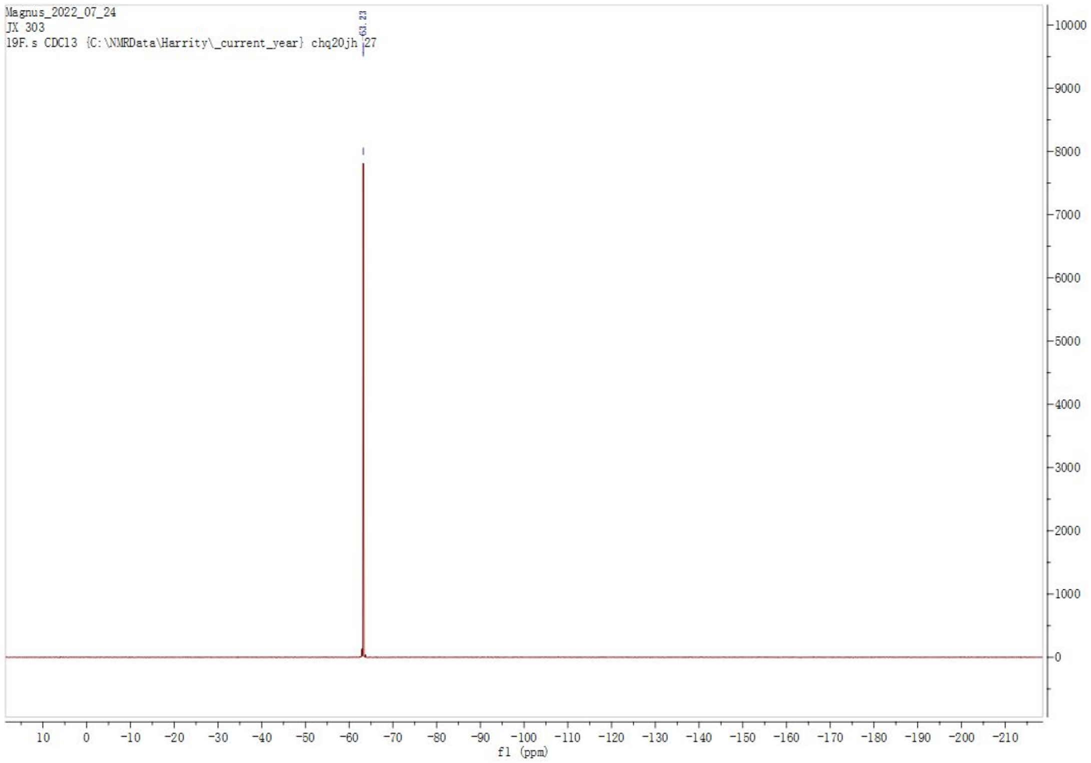

$^{13}C\{^{1}H\}$ NMR, $CDCl_3$, 101 MHz

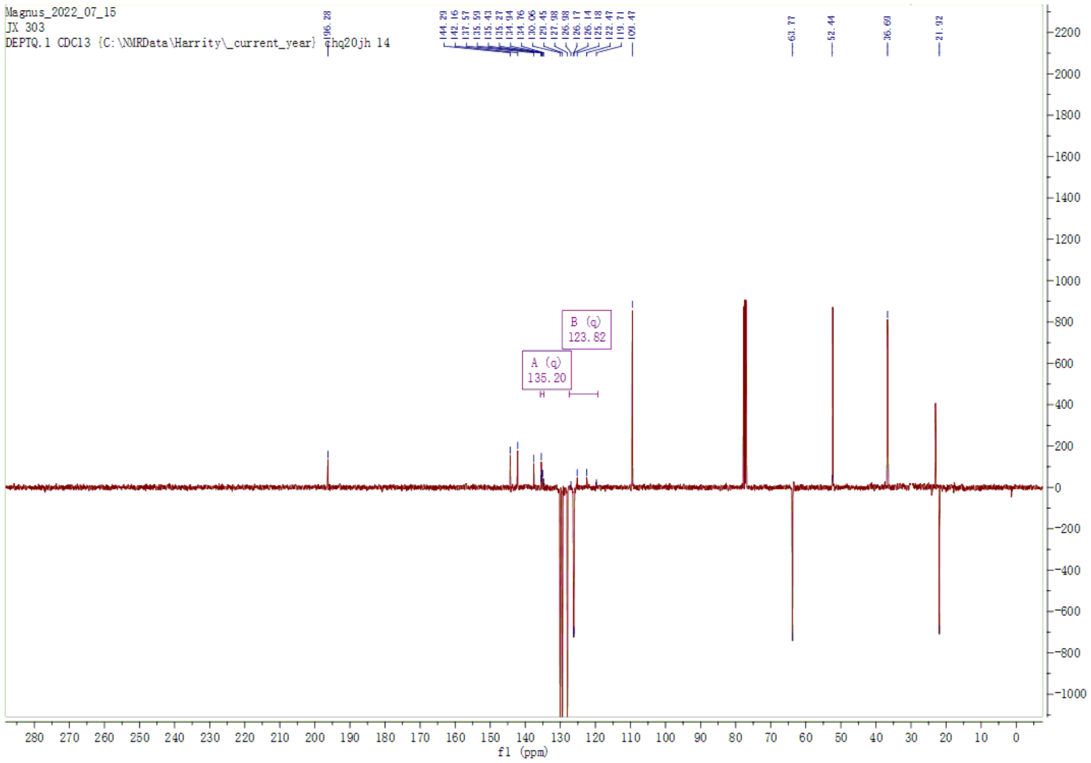

(4-chlorophenyl)(4-methylene-1-tosylpyrrolidin-2-yl)methanone (7)

^{1}H NMR, CDCl$_3$, 400 MHz

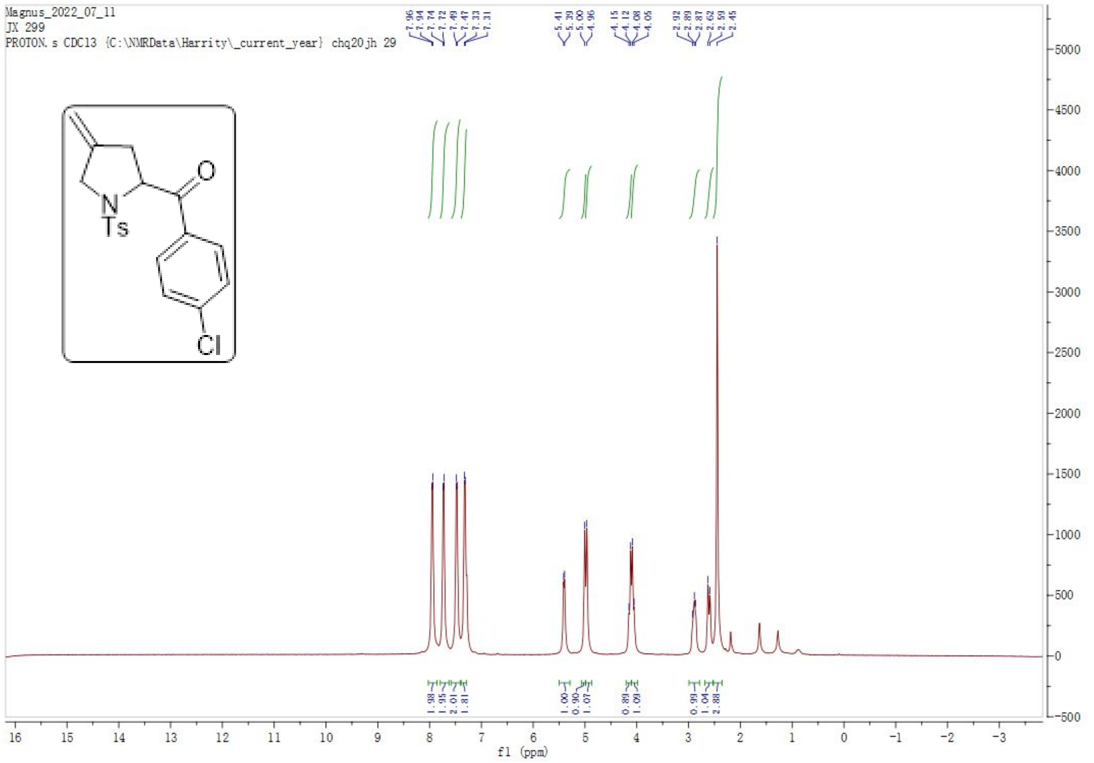

^{13}C{^{1}H} NMR, CDCl$_3$, 101 MHz

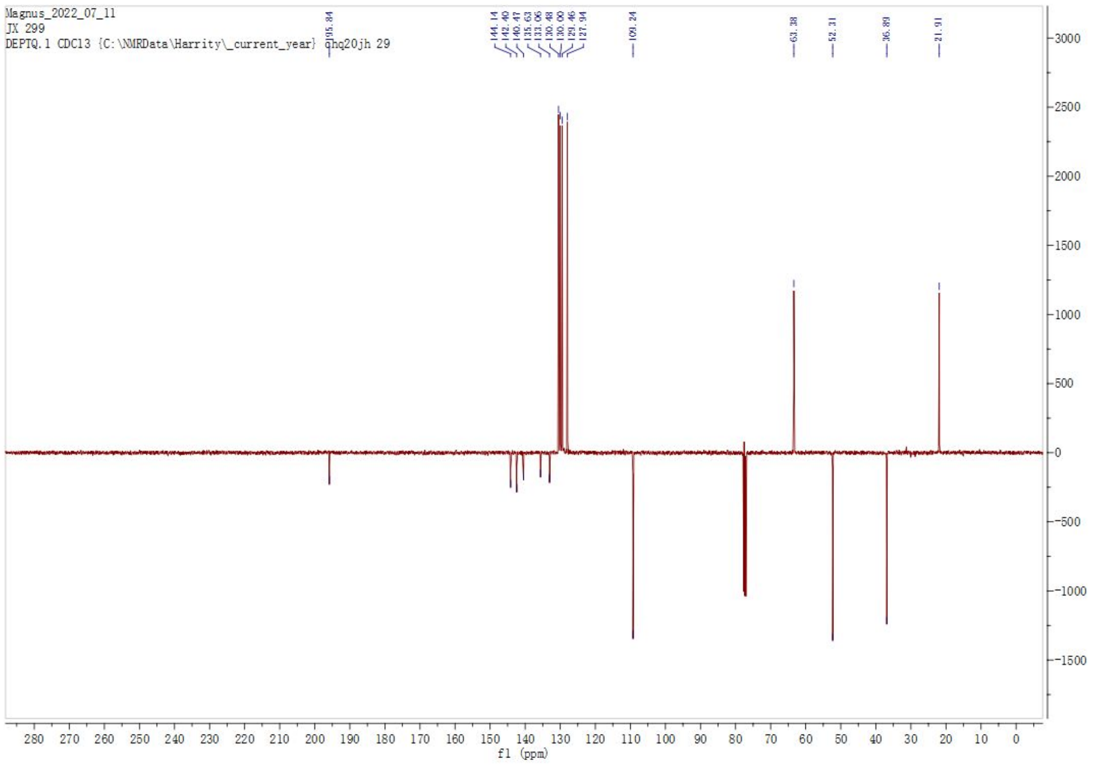

(4-fluorophenyl)(4-methylene-1-tosylpyrrolidin-2-yl)methanone (8)

^{1}H NMR, $CDCl_3$, 400 MHz

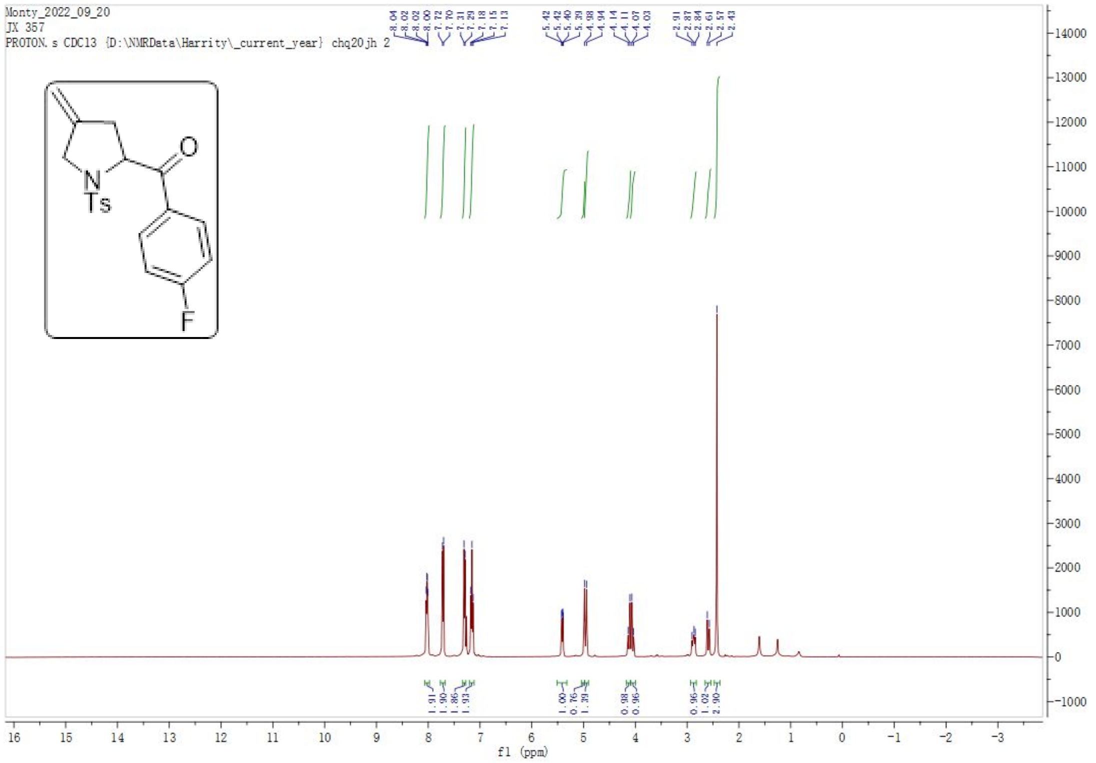

^{19}F NMR, $CDCl_3$, 377 MHz

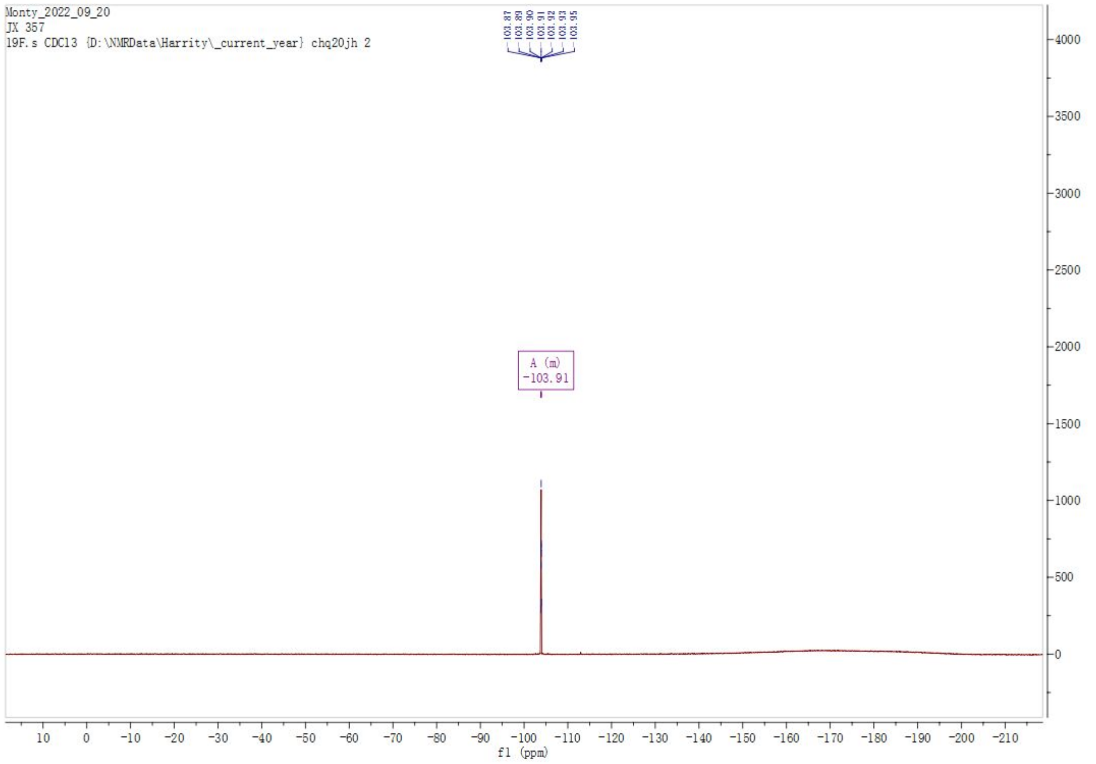

$^{13}C\{^{1}H\}$ NMR, $CDCl_3$, 101 MHz

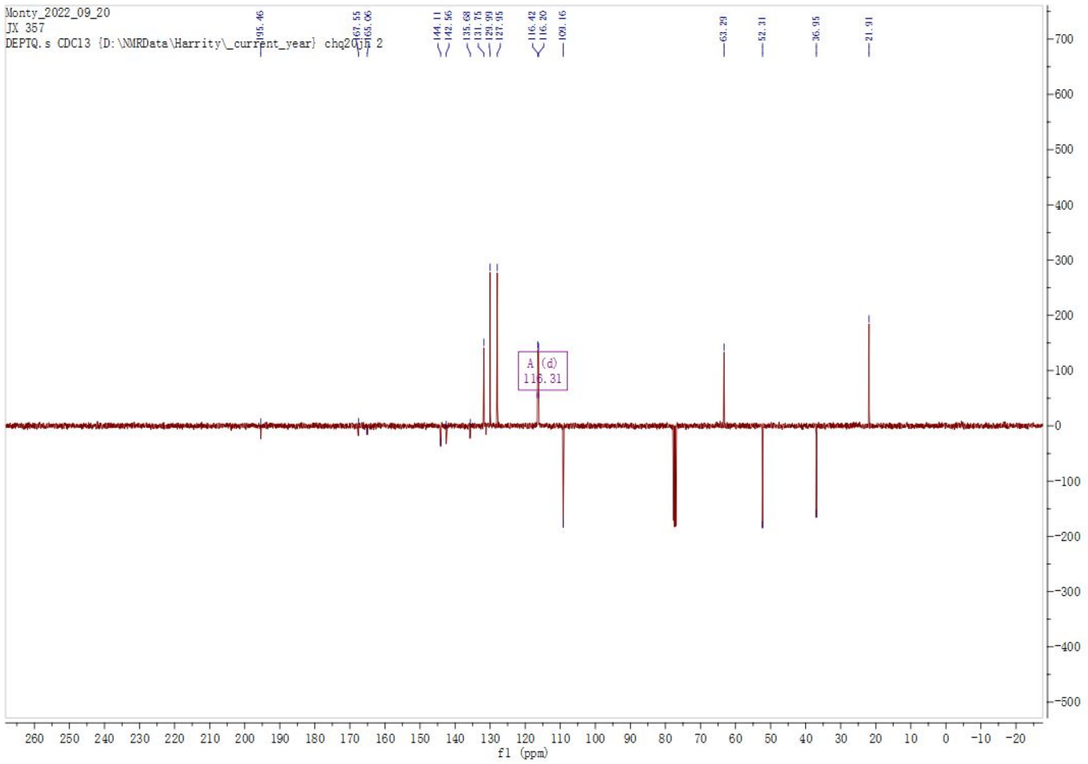

4-methylene-2-(4-nitrophenyl)-1-tosylpyrrolidine (9)

^{1}H NMR, $CDCl_3$, 400 MHz

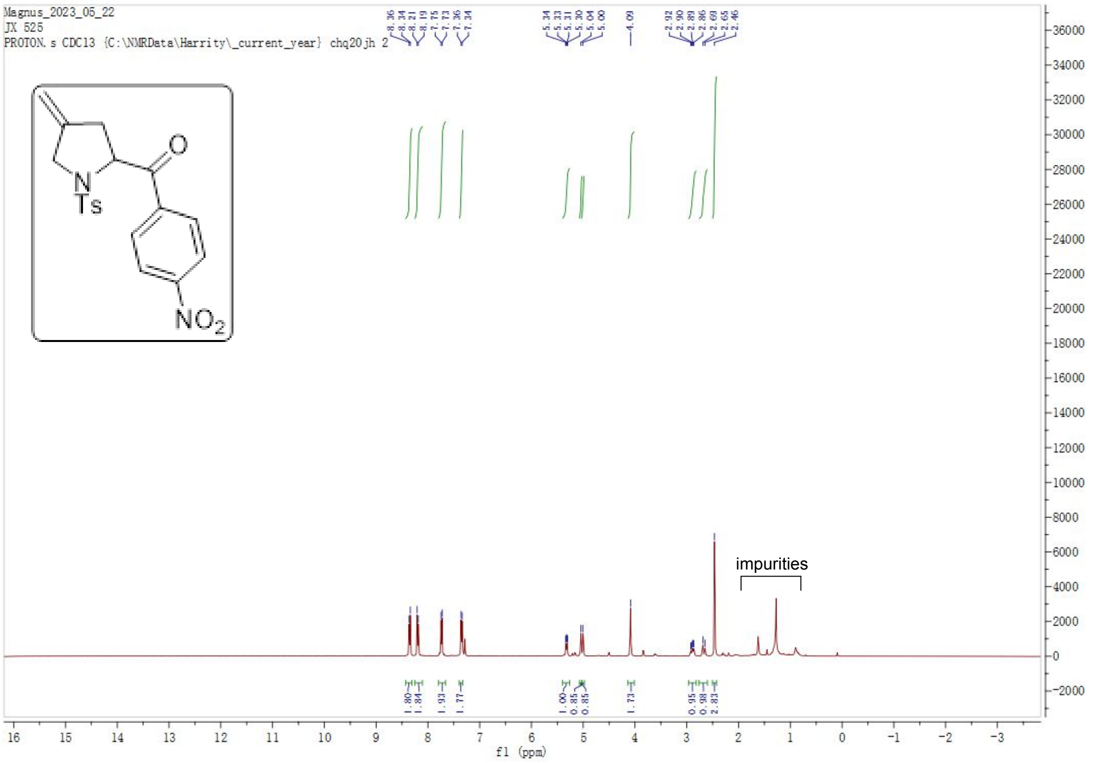

$^{13}C\{^{1}H\}$ NMR, $CDCl_3$, 101 MHz

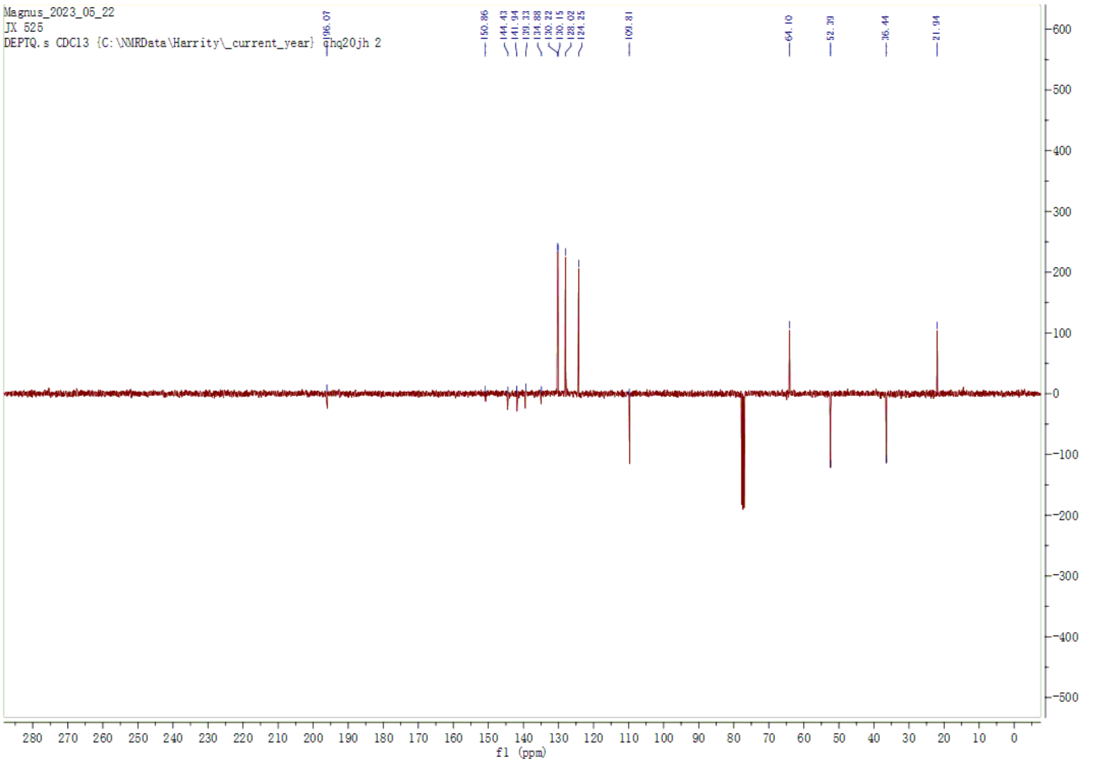

4-(4-methylene-1-tosylpyrrolidine-2-carbonyl)benzonitrile (10)

^{1}H NMR, $CDCl_3$, 400 MHz

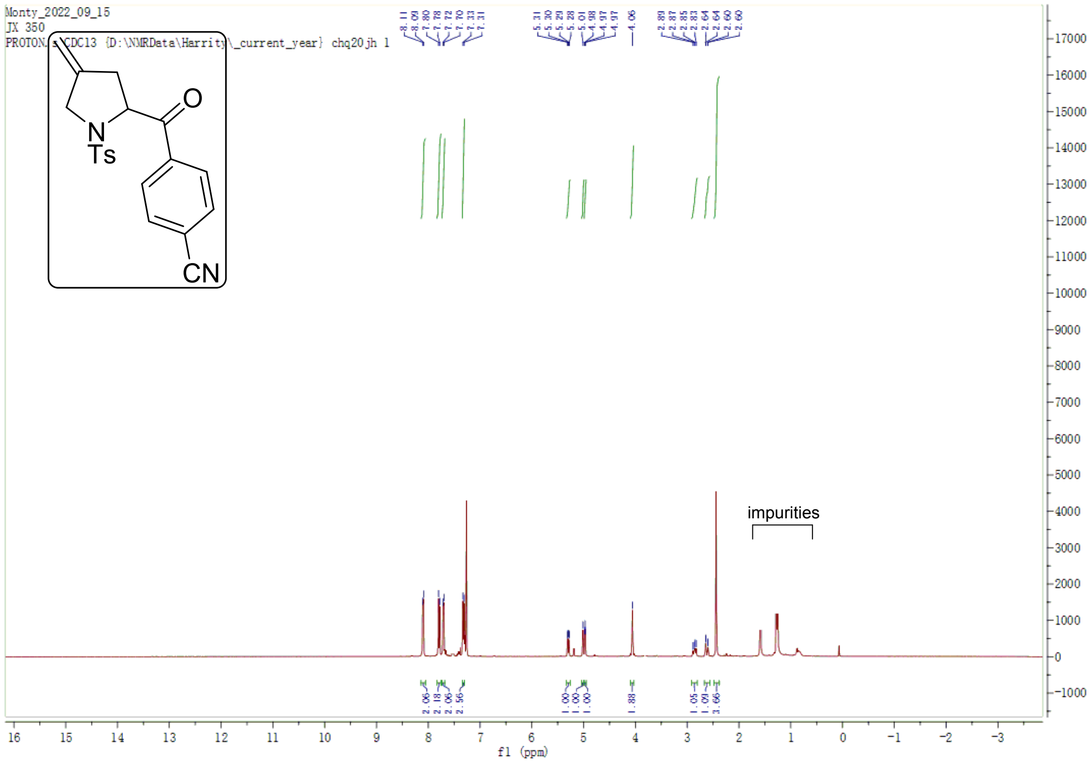

(2-methoxyphenyl)(4-methylene-1-tosylpyrrolidin-2-yl)methanone (11)

^{1}H NMR, $CDCl_3$, 400 MHz

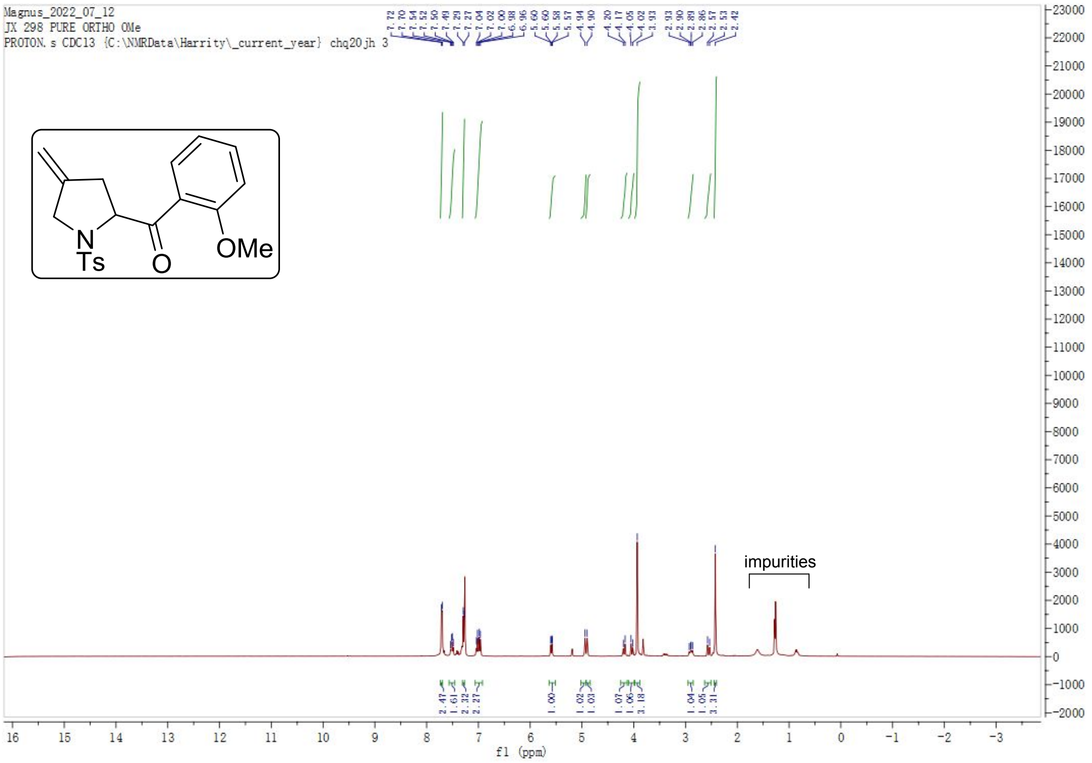

$^{13}C\{^{1}H\}$ NMR, $CDCl_3$, 101 MHz

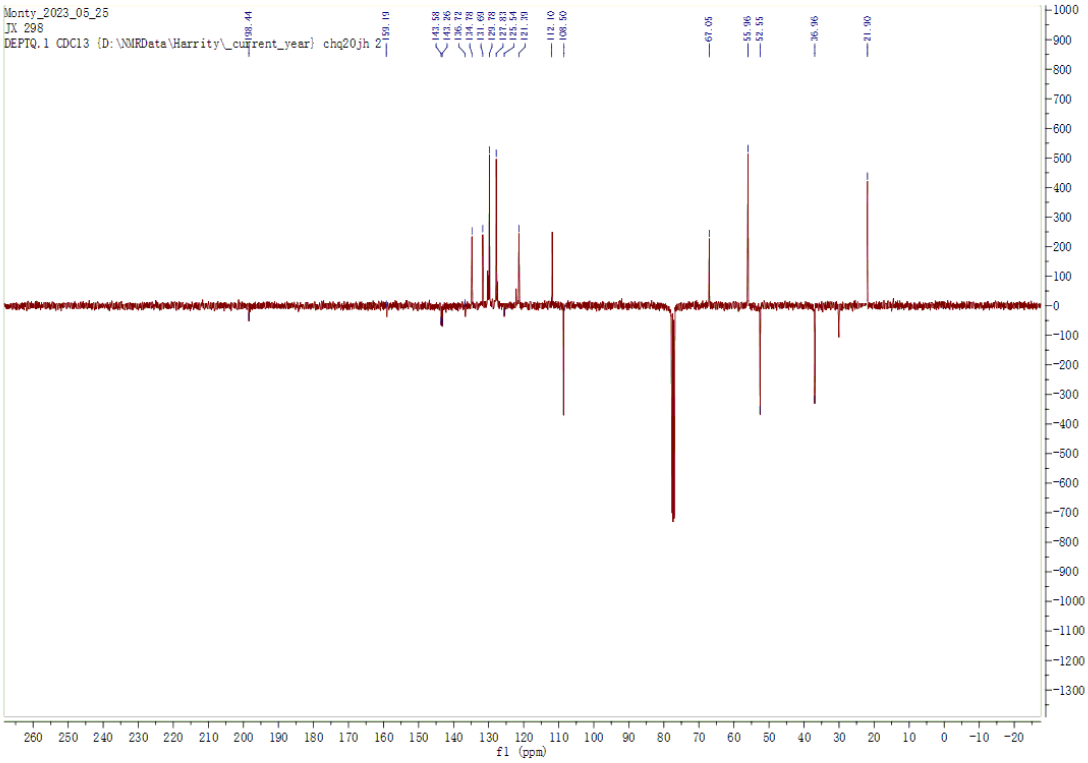

(3-methoxyphenyl)(4-methylene-1-tosylpyrrolidin-2-yl)methanone (12)

^{1}H NMR, $CDCl_3$, 400 MHz

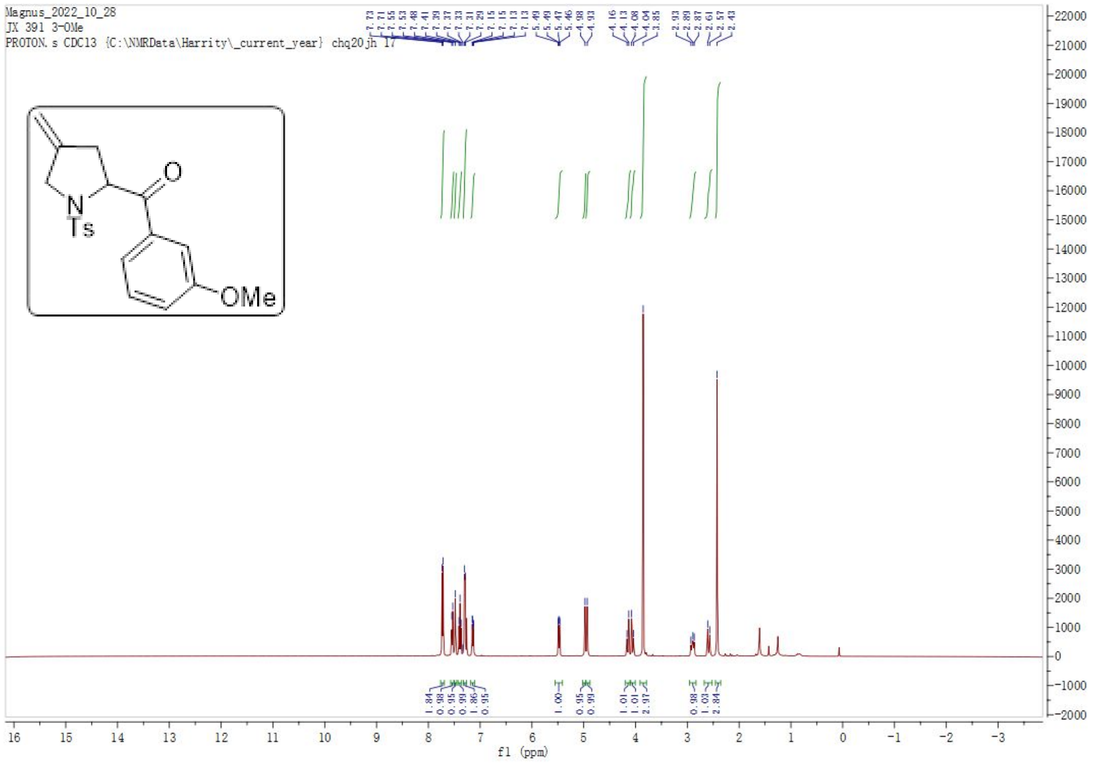

$^{13}C\{^{1}H\}$ NMR, $CDCl_3$, 101 MHz

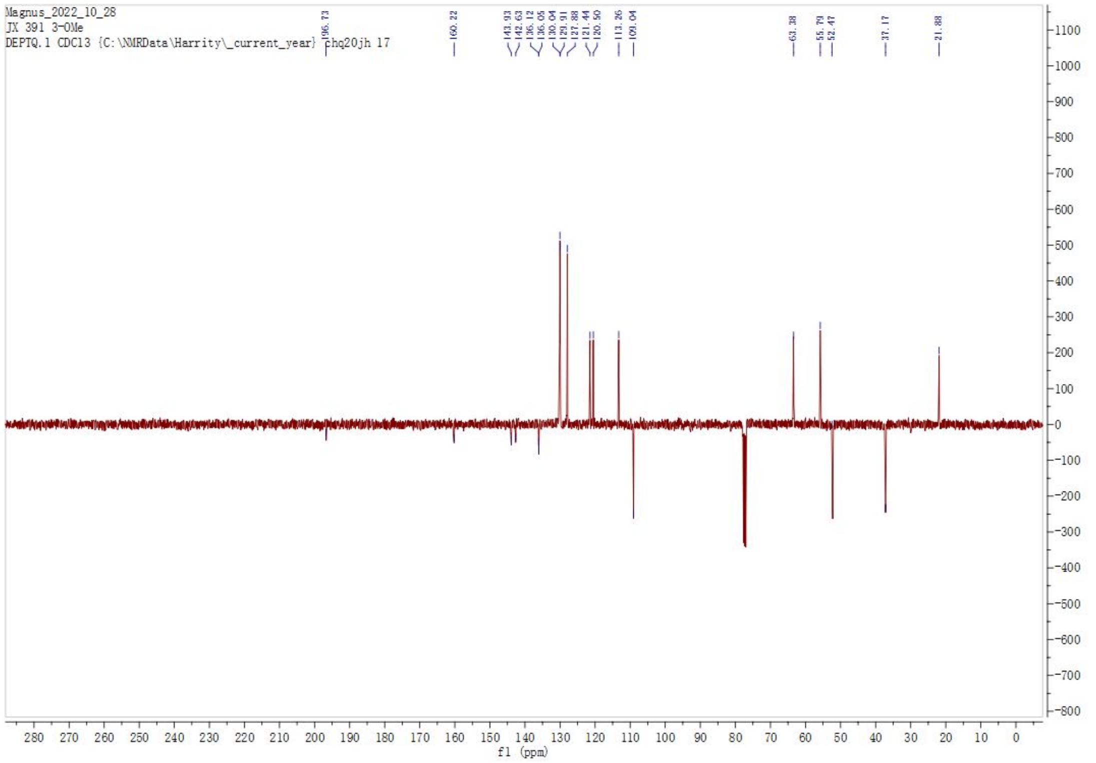

(3-fluorophenyl)(4-methylene-1-tosylpyrrolidin-2-yl)methanone (13)

^{1}H NMR, $CDCl_3$, 400 MHz

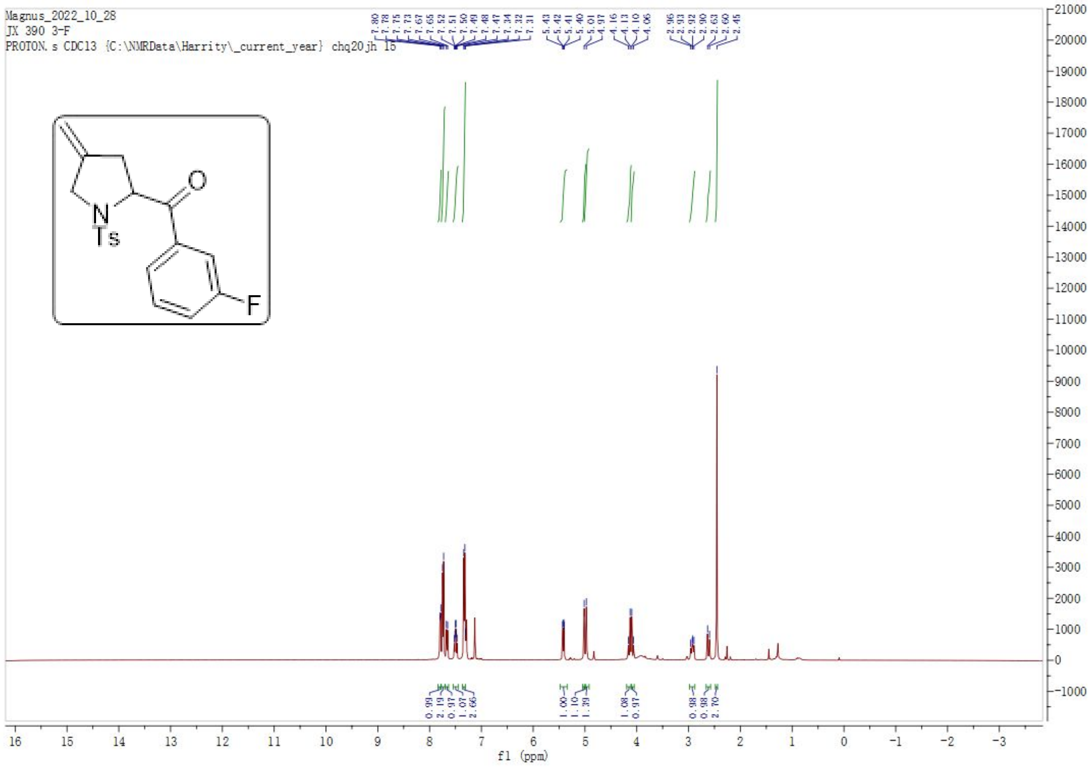

^{19}F NMR, $CDCl_3$, 377 MHz

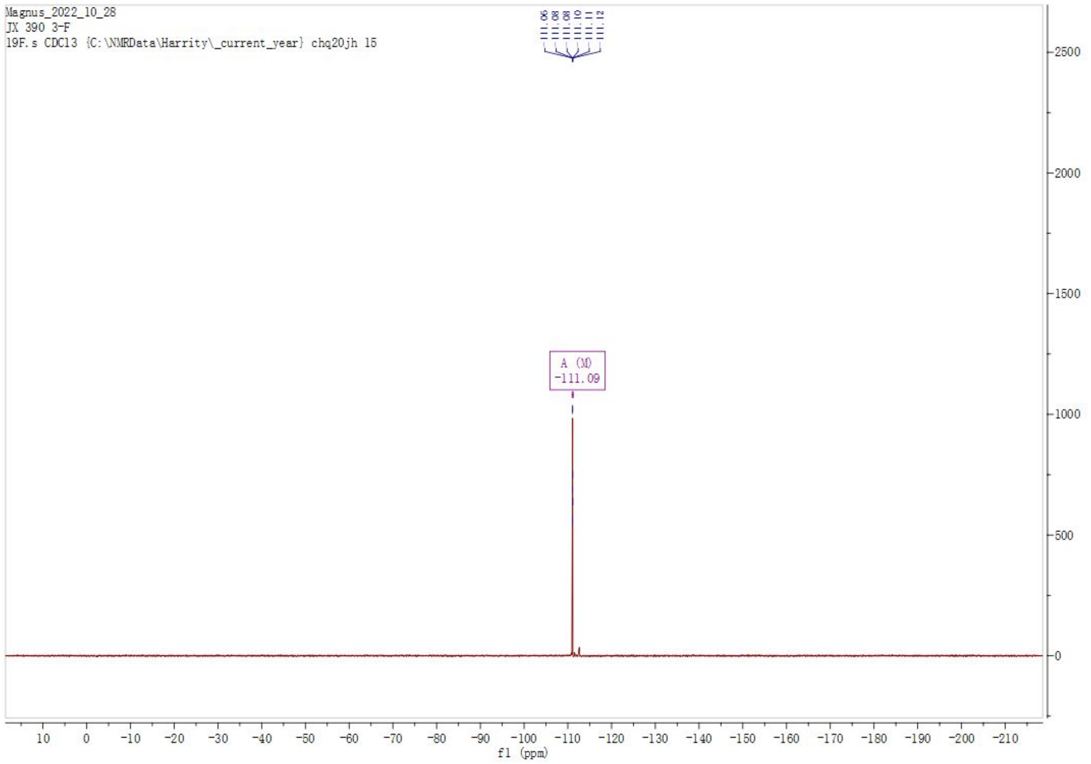

$^{13}C\{^{1}H\}$ NMR, $CDCl_3$, 101 MHz

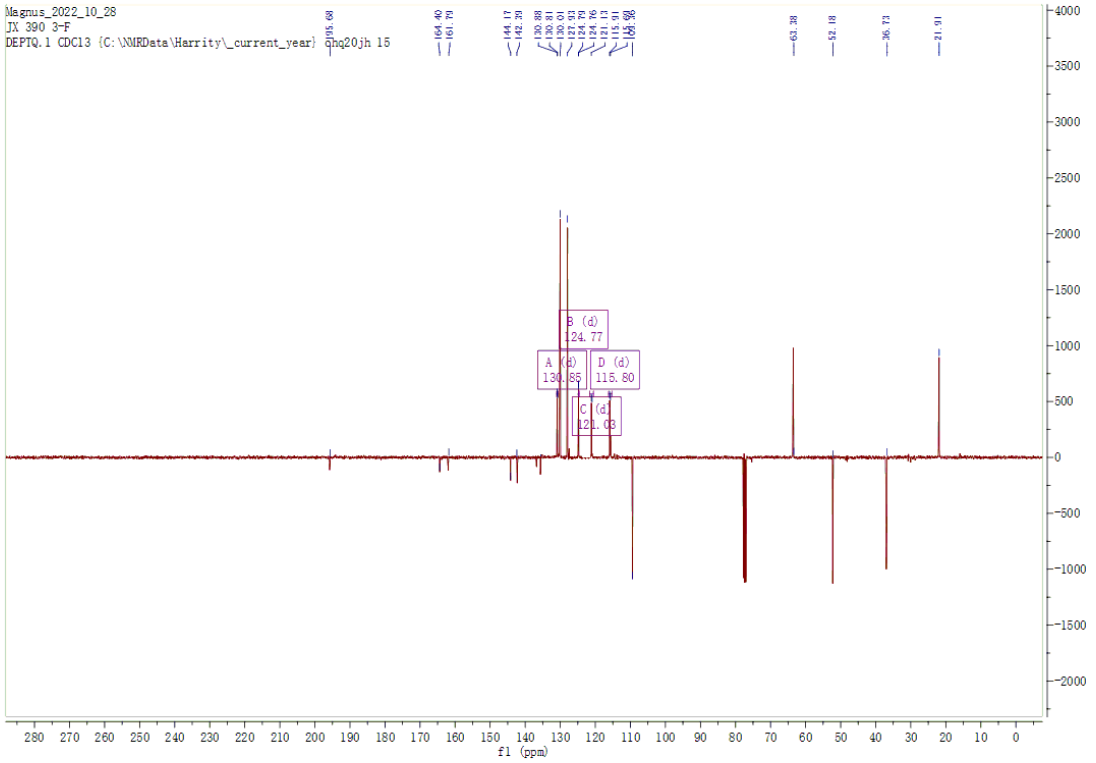

(4-methylene-1-tosylpyrrolidin-2-yl)(naphthalen-1-yl)methanone (14)

^{1}H NMR, $CDCl_3$, 400 MHz

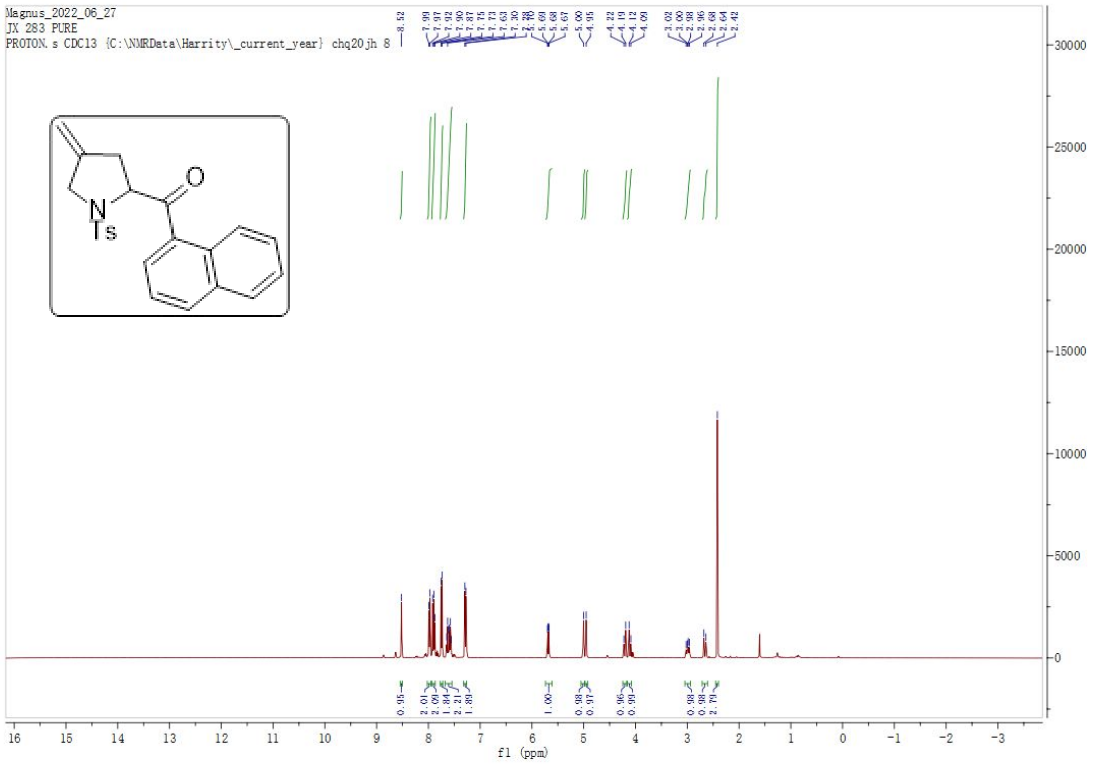

^{13}C{^{1}H} NMR, $CDCl_3$, 101 MHz

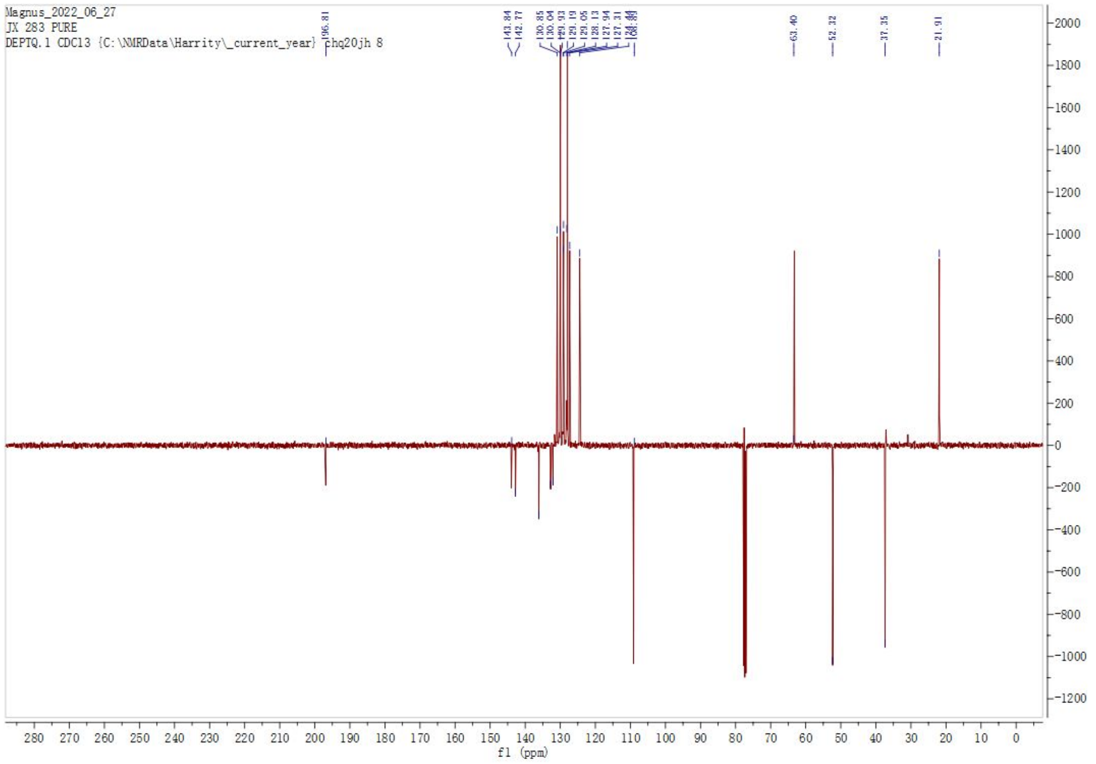

(4-methylene-1-tosylpyrrolidin-2-yl)(thiophen-2-yl)methanone (15)

^{1}H NMR, CDCl$_3$, 400 MHz

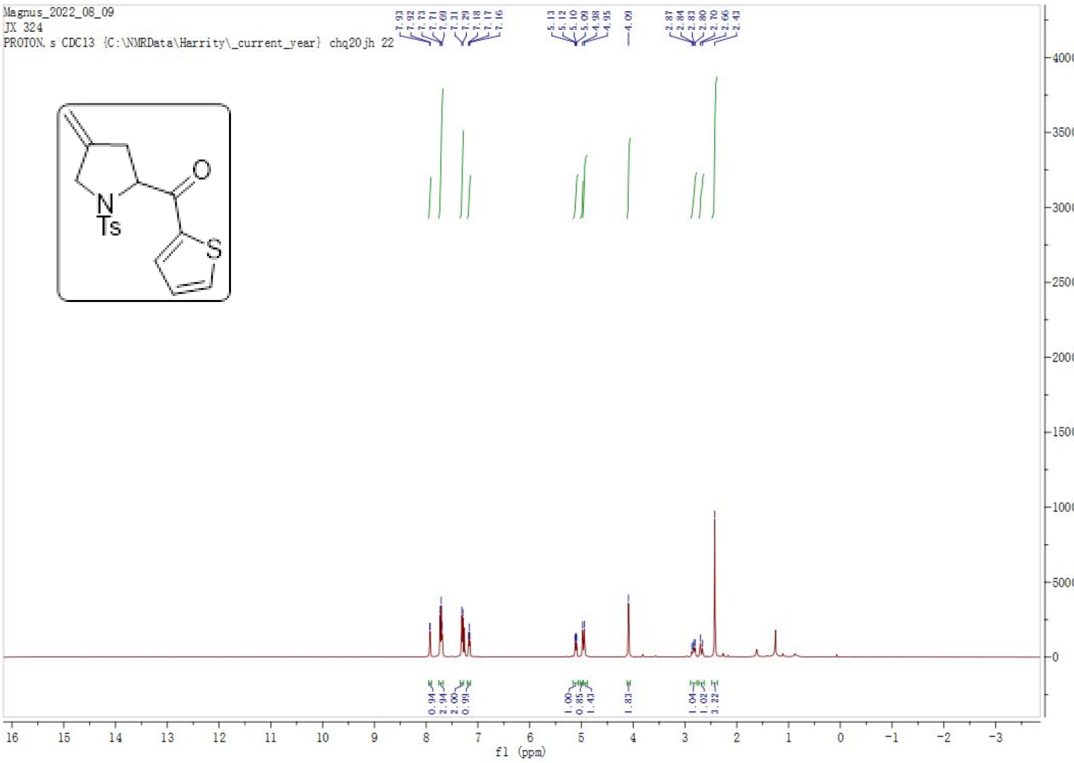

^{13}C{^{1}H} NMR, CDCl$_3$, 101 MHz

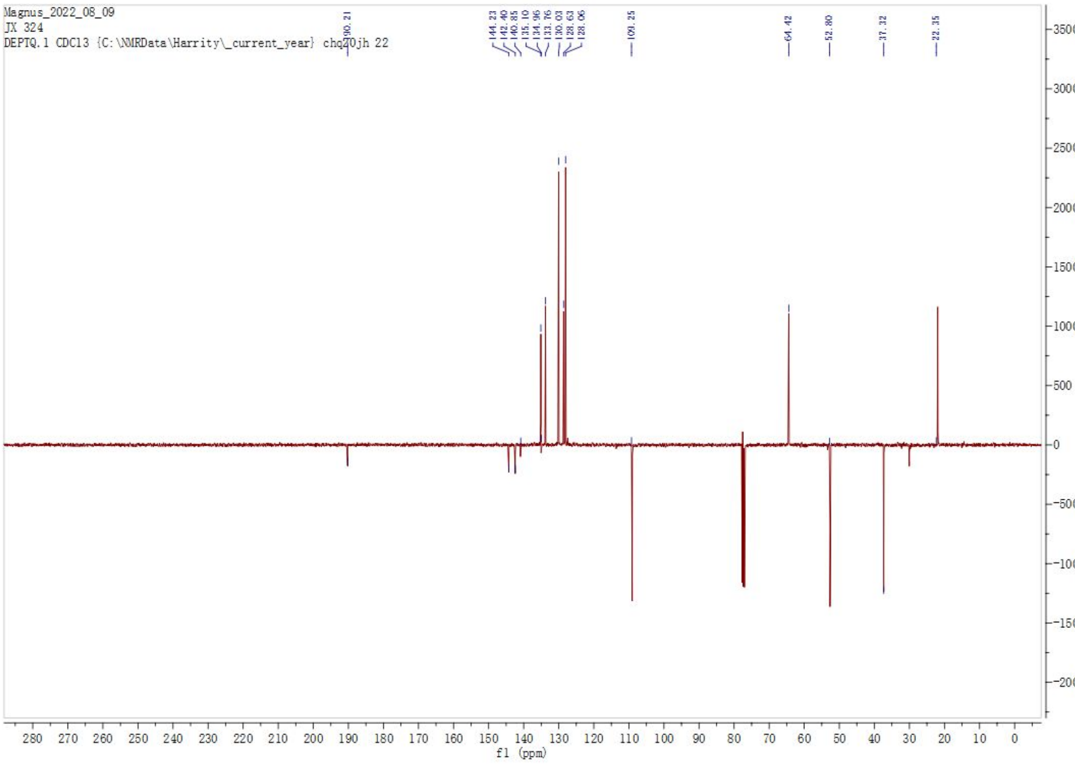

2,2-dimethyl-1-(4-methylene-1-tosylpyrrolidin-2-yl)propan-1-one (16)

^{1}H NMR, $CDCl_3$, 400 MHz

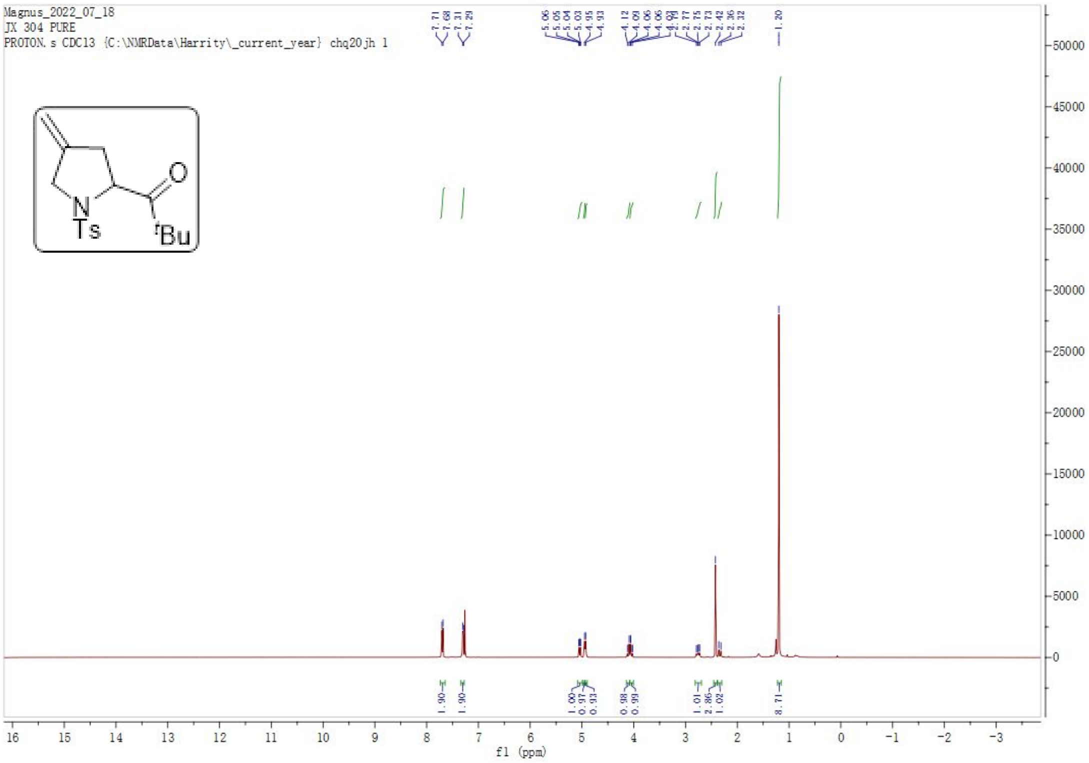

ethyl 4-methylene-1-tosylpyrrolidine-2-carboxylate (17)

^{1}H NMR, $CDCl_3$, 400 MHz

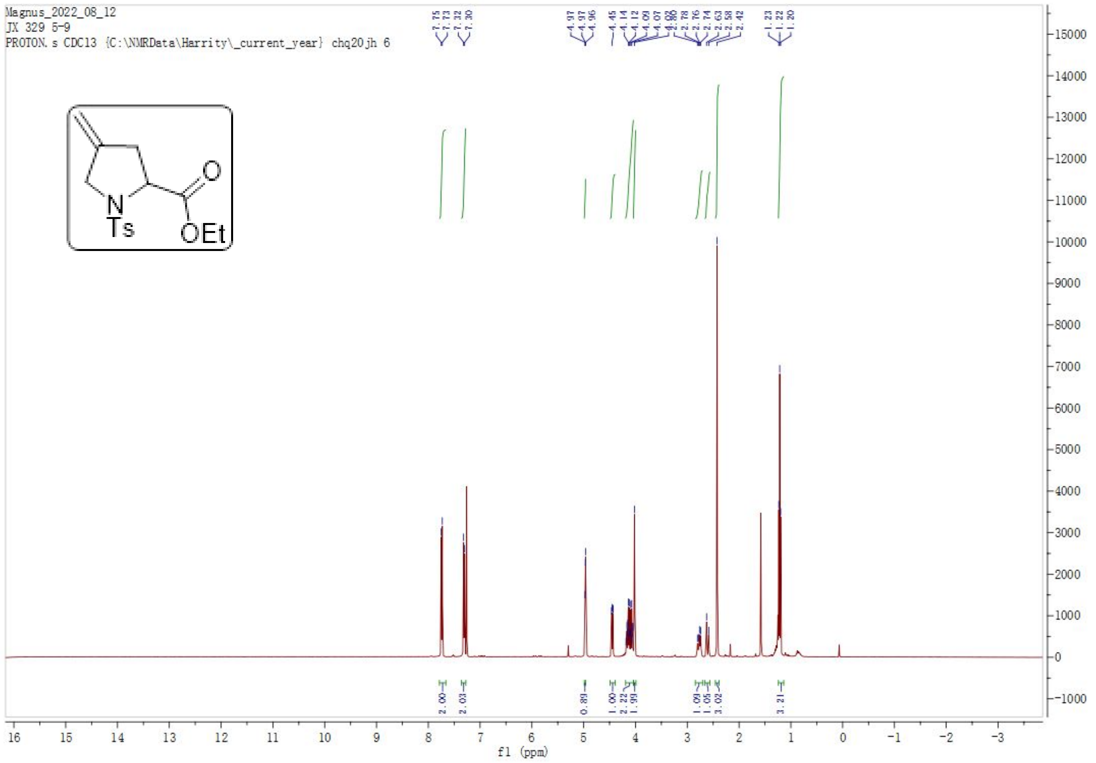

$^{13}C\{^{1}H\}$ NMR, $CDCl_3$, 101 MHz

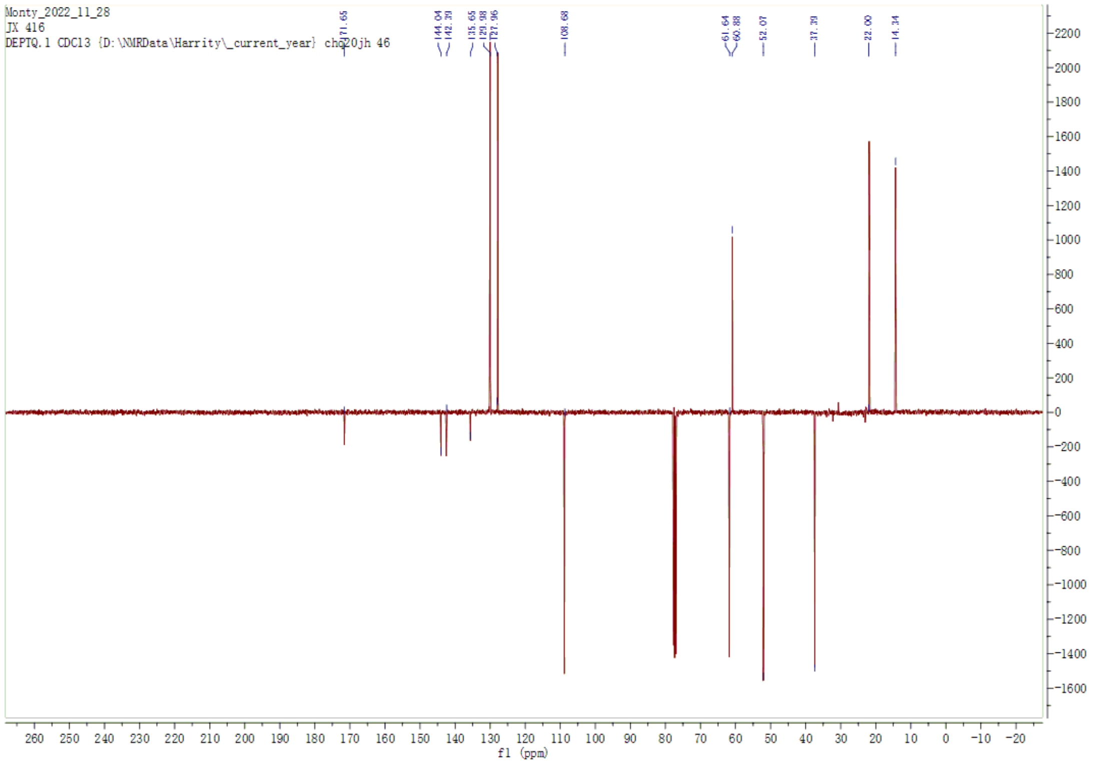

1-(4-methylene-1-tosylpyrrolidin-2-yl)propan-1-one (18)

^{1}H NMR, CDCl$_3$, 400 MHz

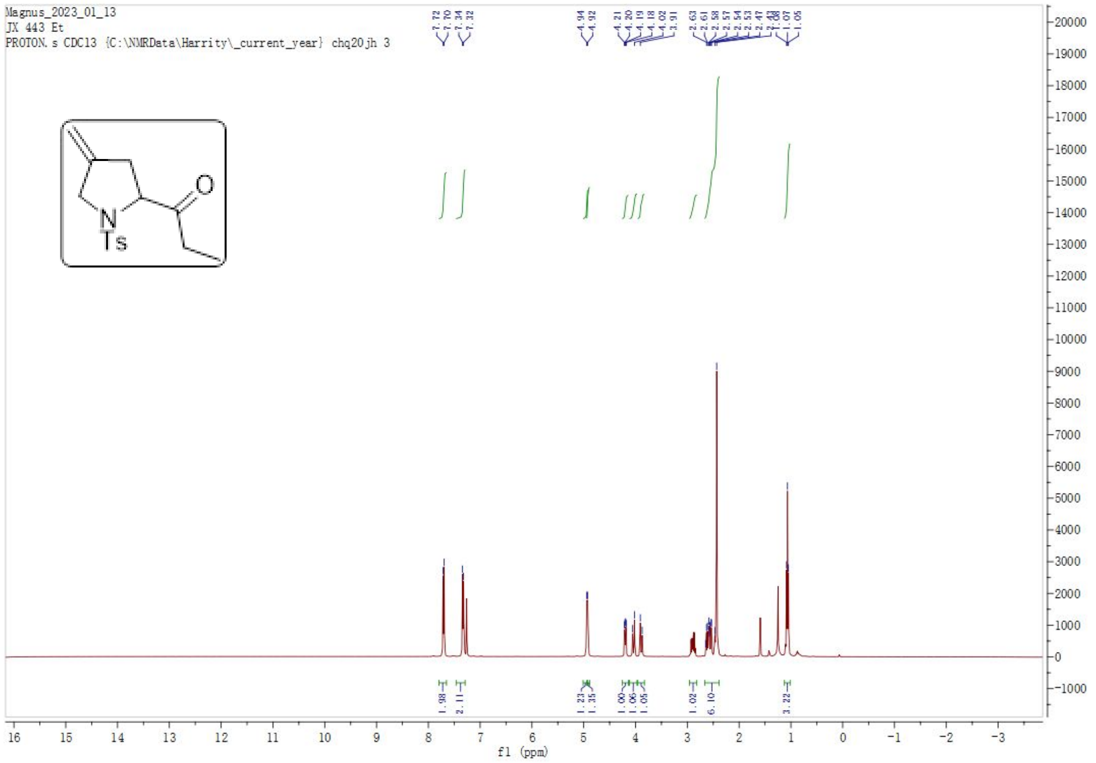

^{13}C{^{1}H} NMR, CDCl$_3$, 101 MHz

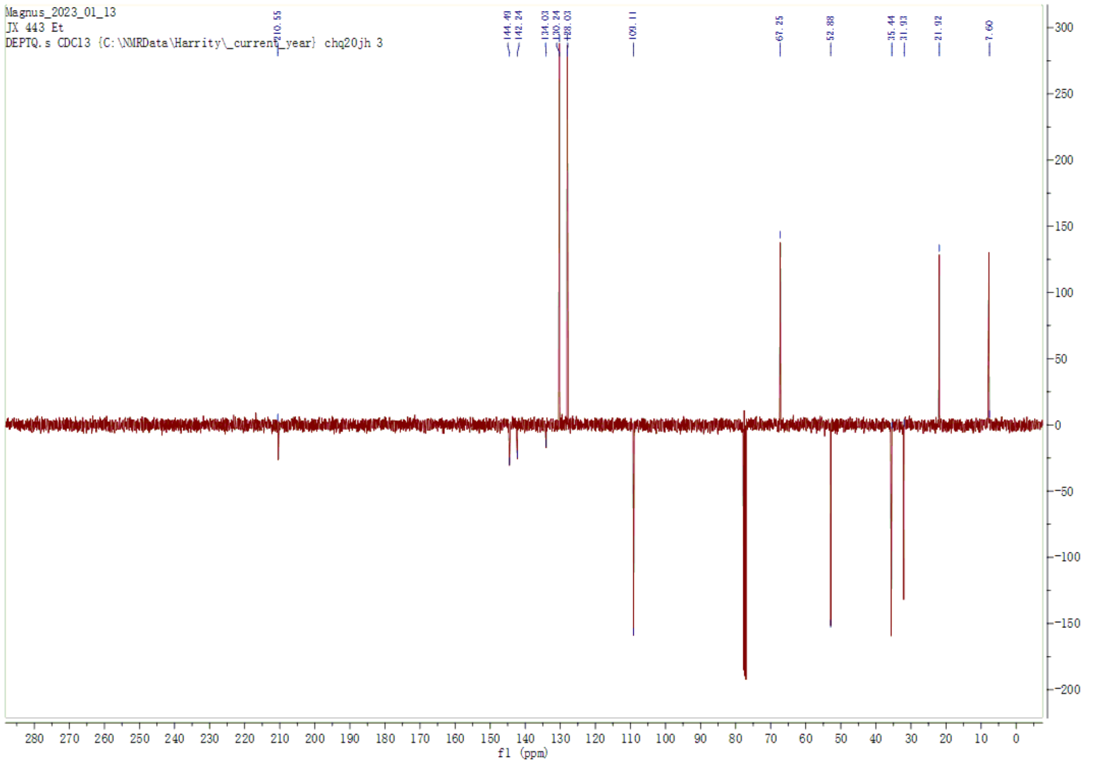

(*S*)-4-isopropyl-3-((*R*)-4-methylene-1-tosylpyrrolidine-2-carbonyl)oxazolidin-2-one (20)

^{1}H NMR, $CDCl_3$, 400 MHz

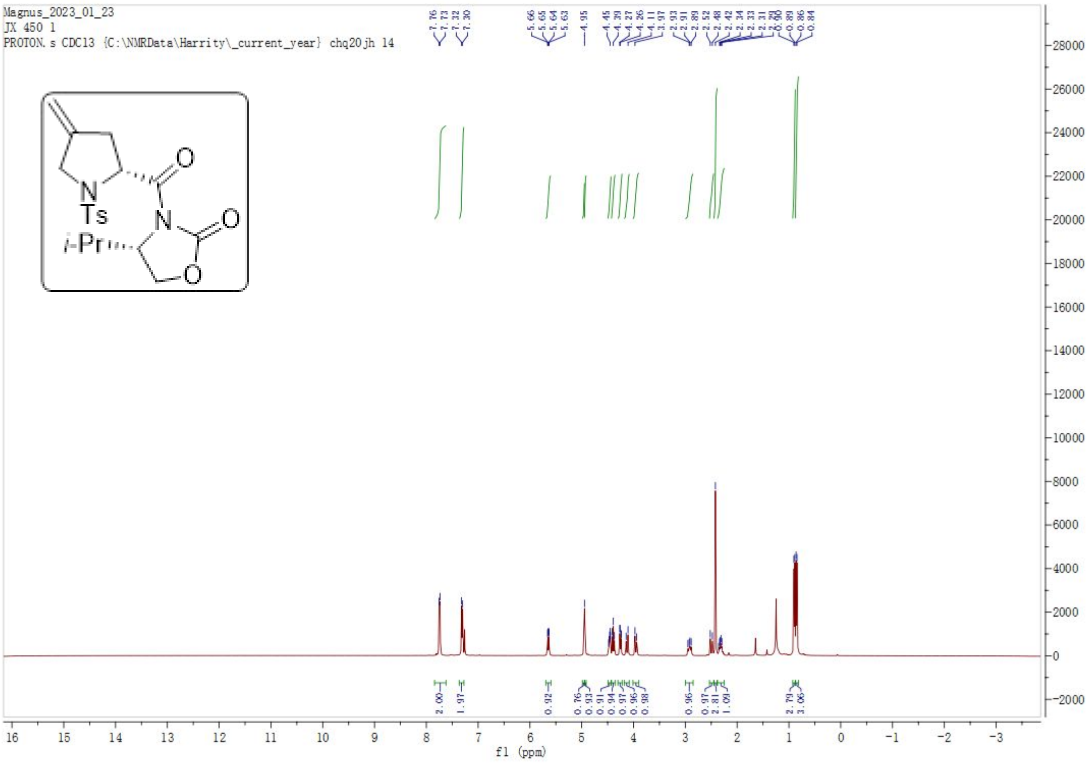

$^{13}C\{^{1}H\}$ NMR, $CDCl_3$, 101 MHz

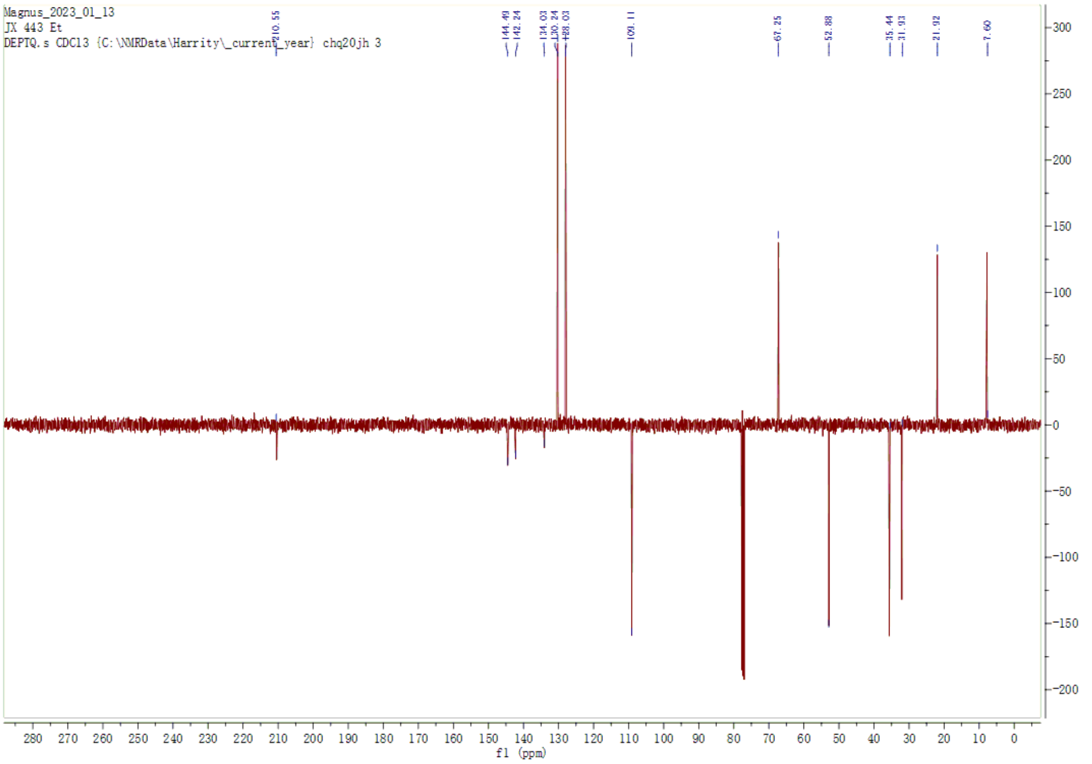

ethyl 4-oxo-1-tosylpyrrolidine-2-carboxylate (21)

^{1}H NMR, $CDCl_3$, 400 MHz

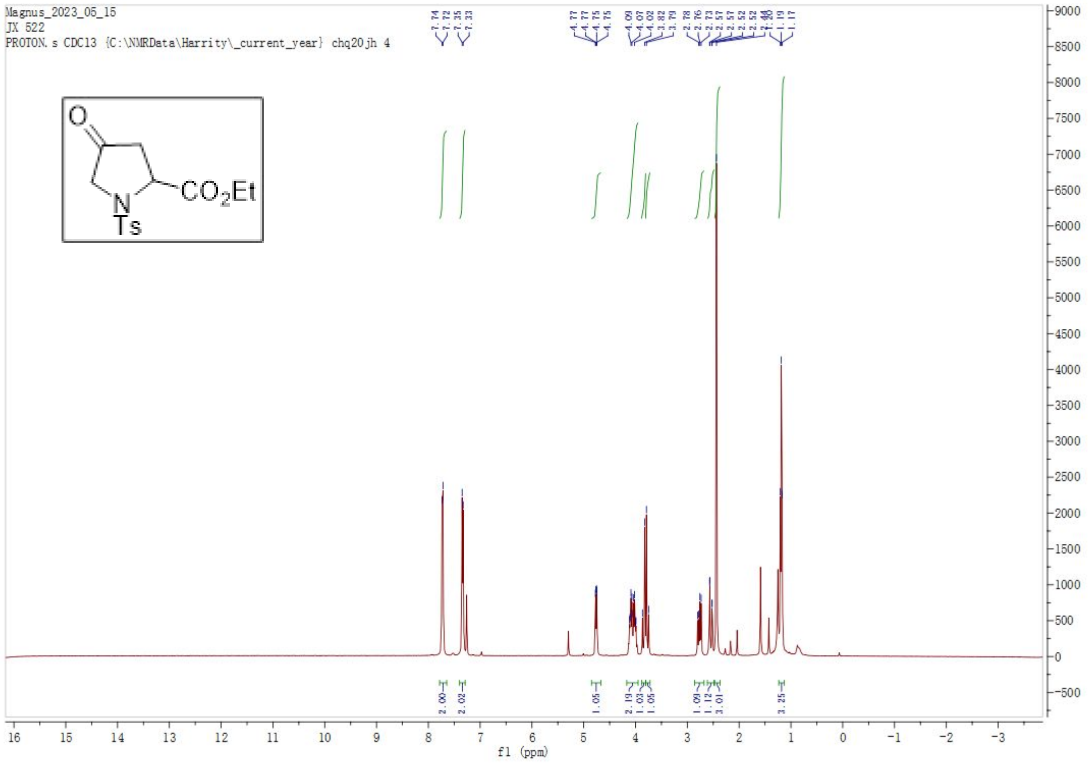

$^{13}C\{^{1}H\}$ NMR, $CDCl_3$, 101 MHz

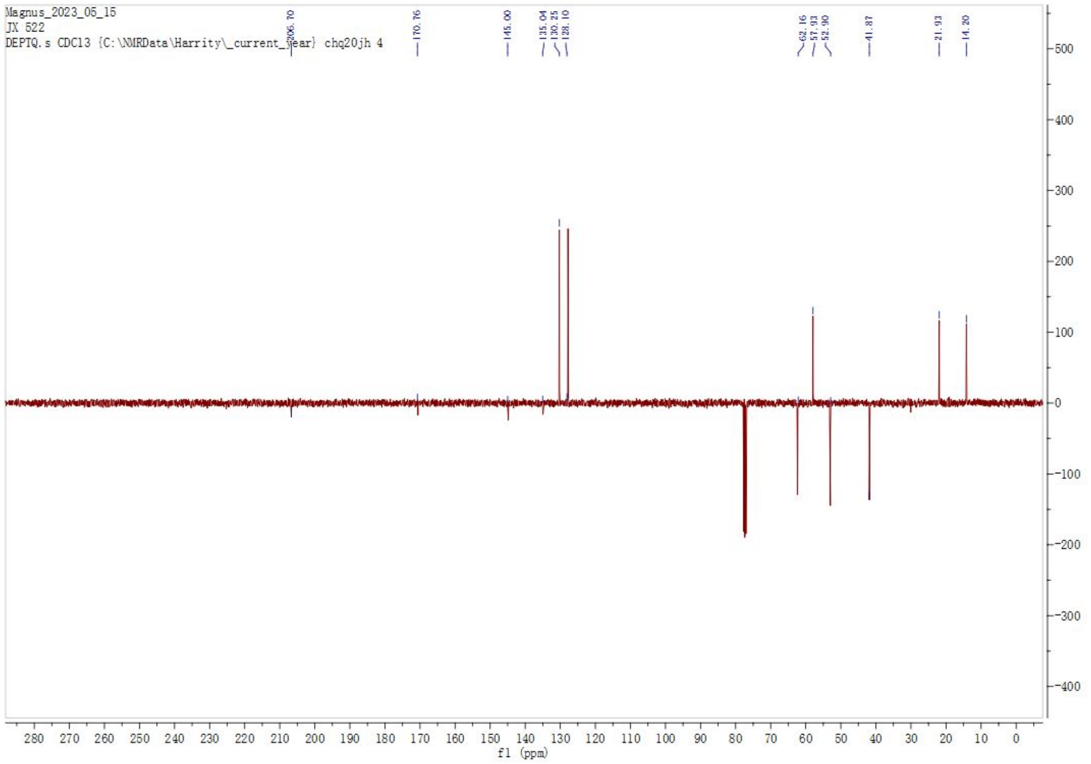

ethyl 4-(propan-2-ylidene)-1-tosylpyrrolidine-2-carboxylate（22）

^{1}H NMR, $CDCl_3$, 400 MHz

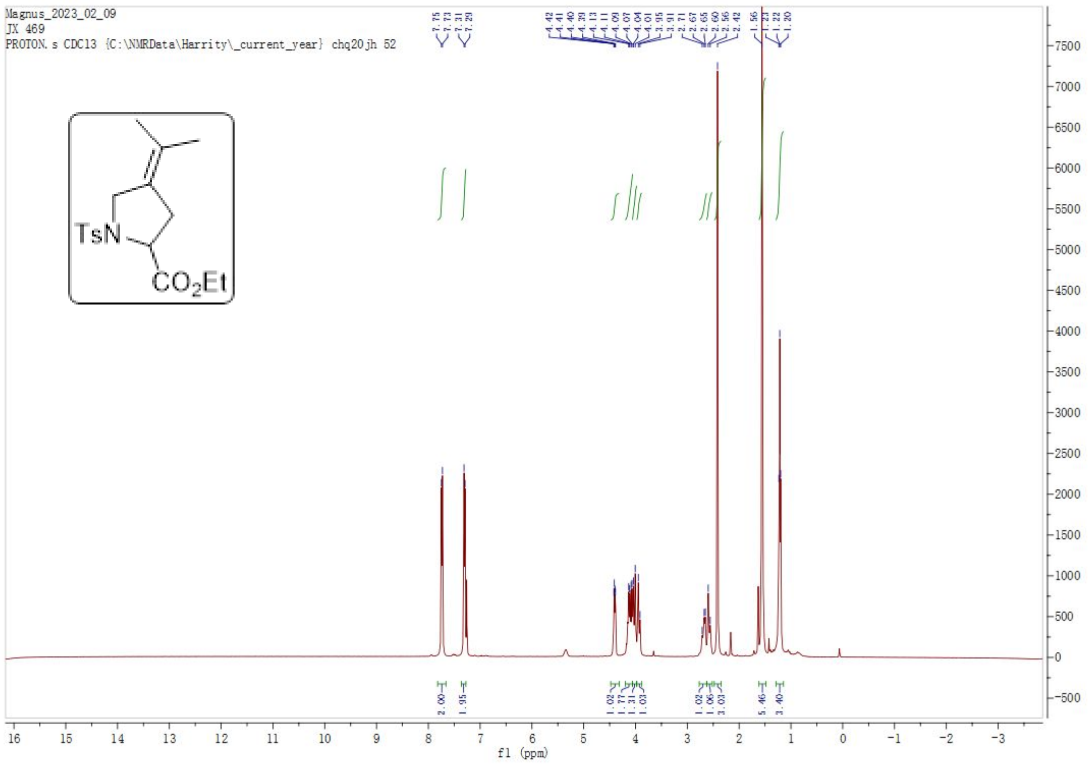

$^{13}C\{^{1}H\}$ NMR, $CDCl_3$, 101 MHz

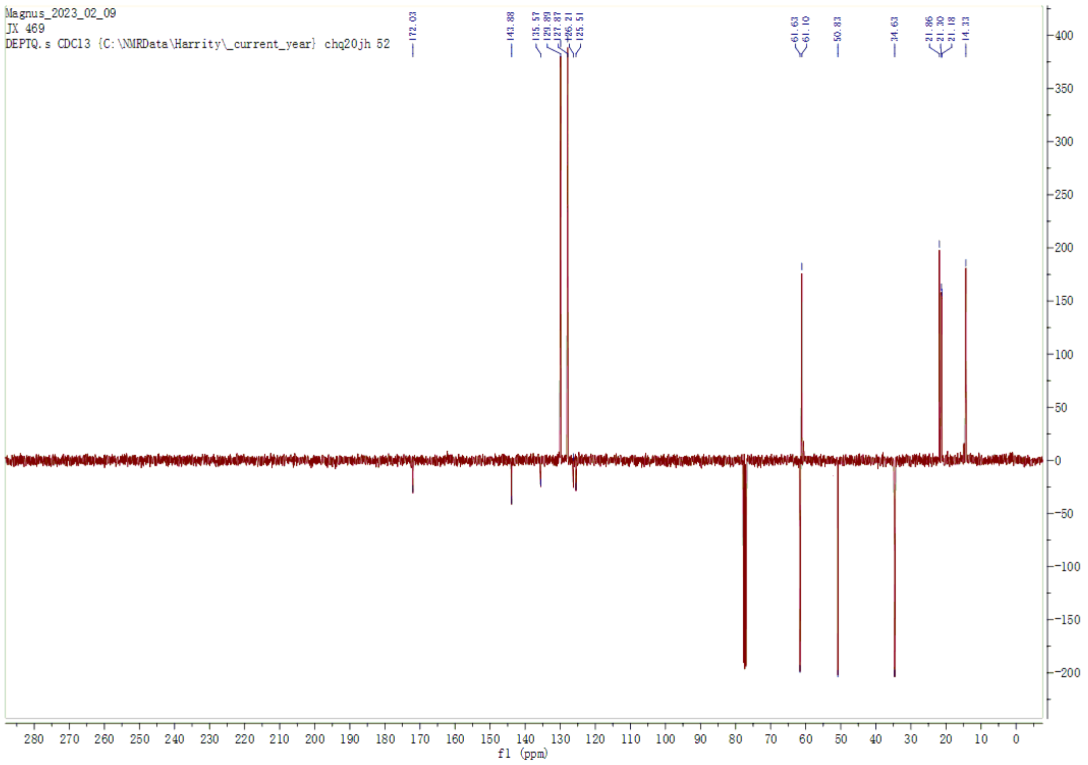

(4-methylene-1-tosylpyrrolidin-2-yl)methanol（23）

^{1}H NMR, $CDCl_3$, 400 MHz

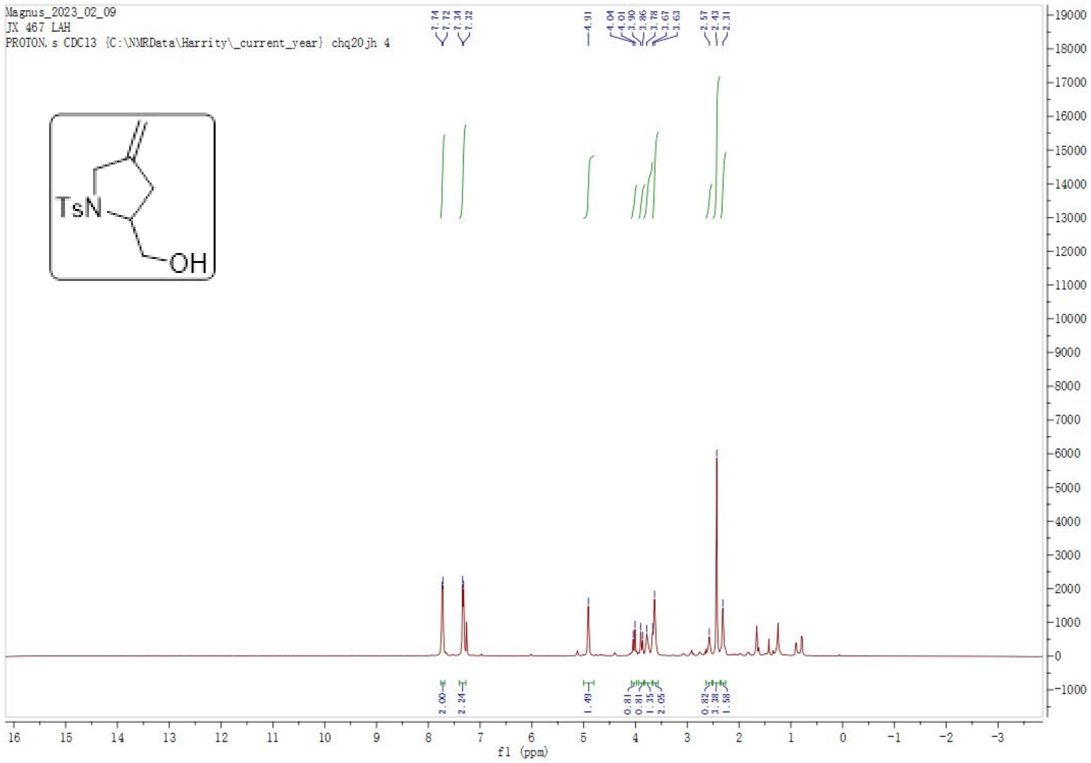

$^{13}C\{^{1}H\}$ NMR, $CDCl_3$, 101 MHz

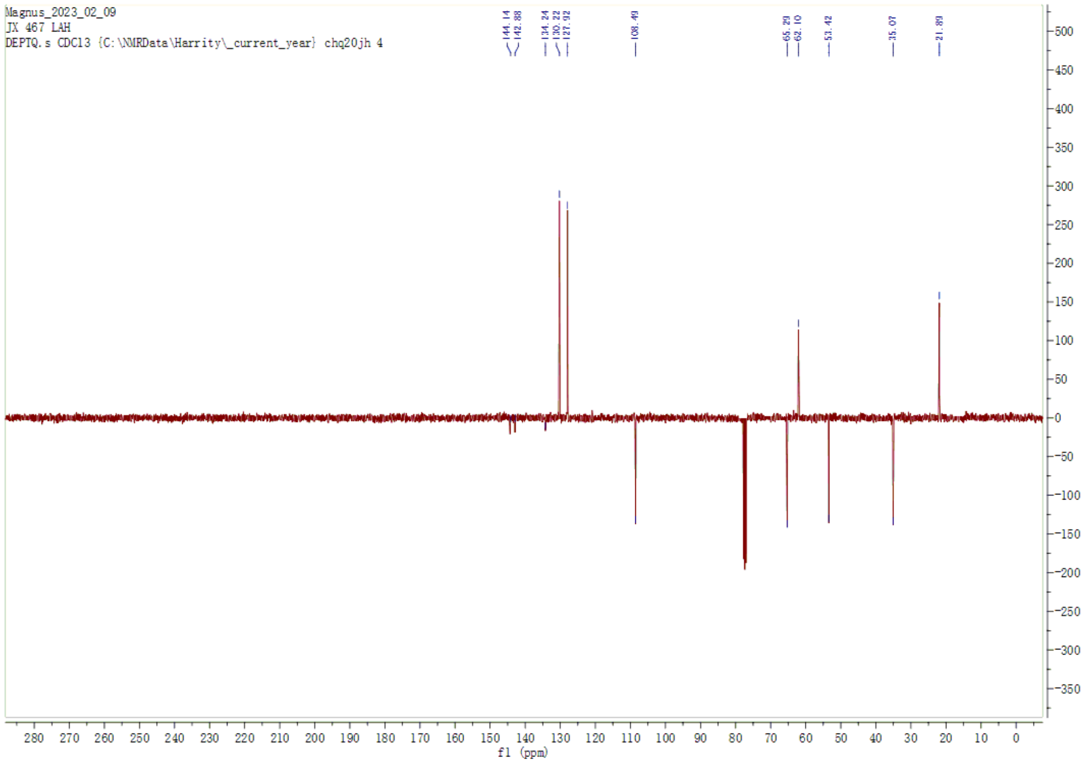

1-(*tert*-butyl) 2-methyl 4-methylenepyrrolidine-1,2-dicarboxylate (24)

^{1}H NMR, $CDCl_3$, 400 MHz

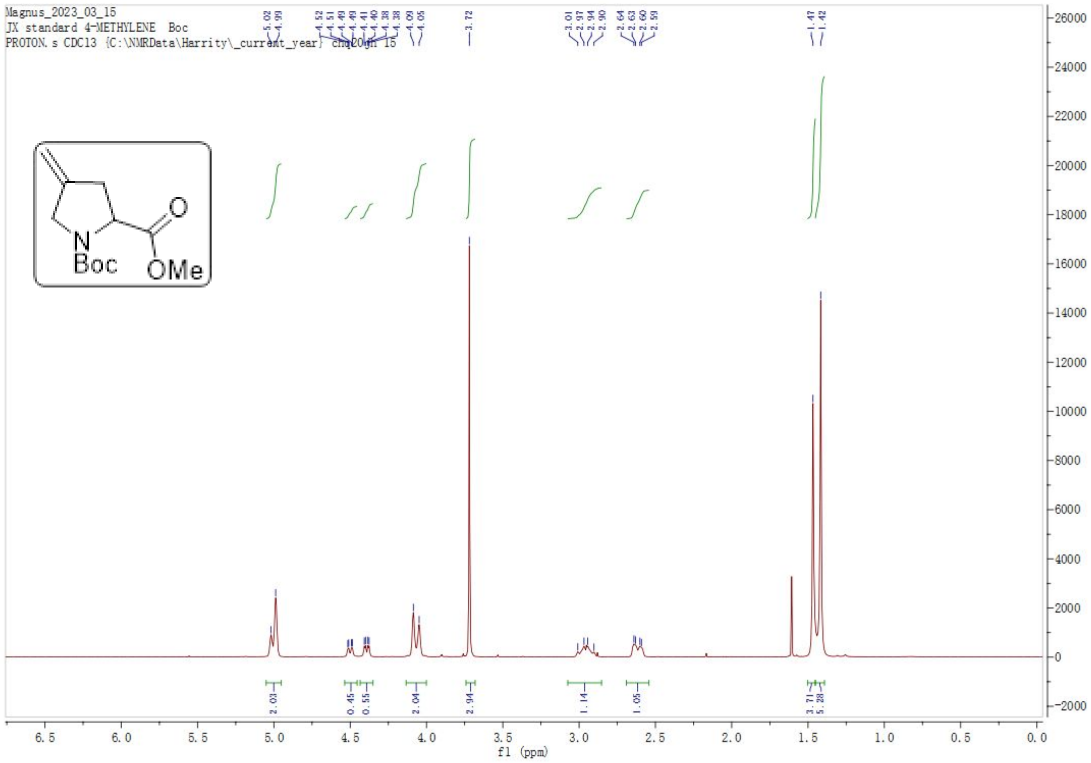

^{13}C{^{1}H} NMR, $CDCl_3$, 101 MHz

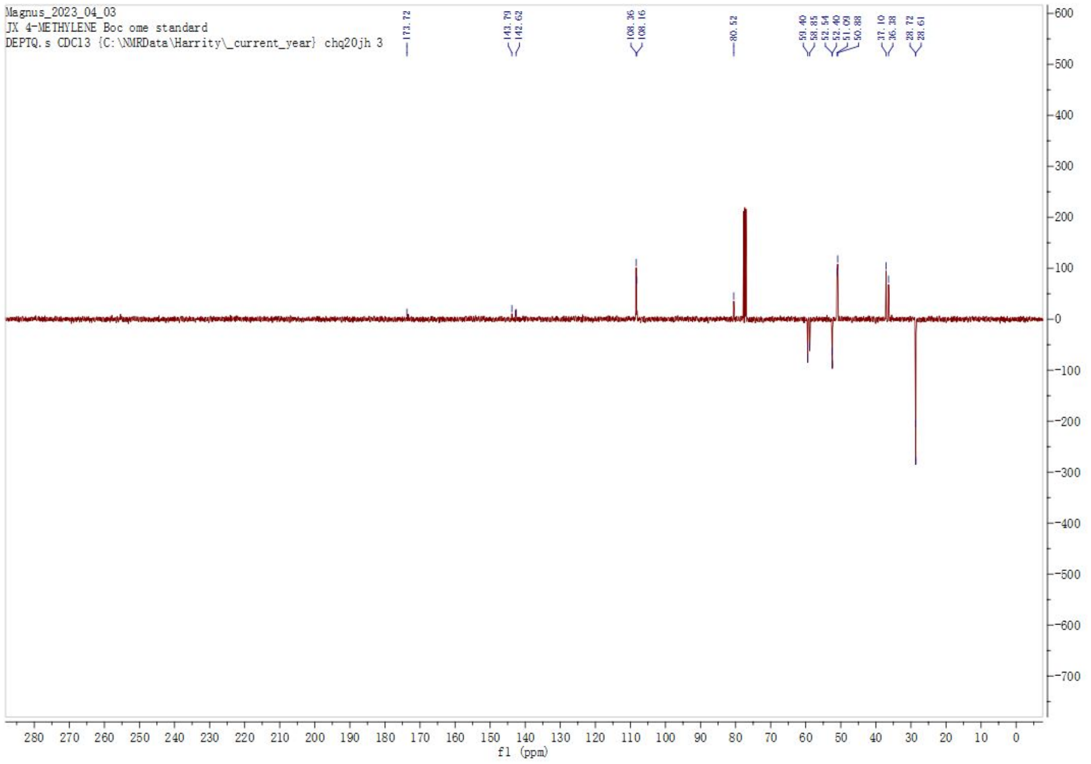

(*R*)-2-(((tert-butyldimethylsilyl)oxy)methyl)-4-methylene-1-tosylpyrrolidine

^{1}H NMR, $CDCl_3$, 400 MHz

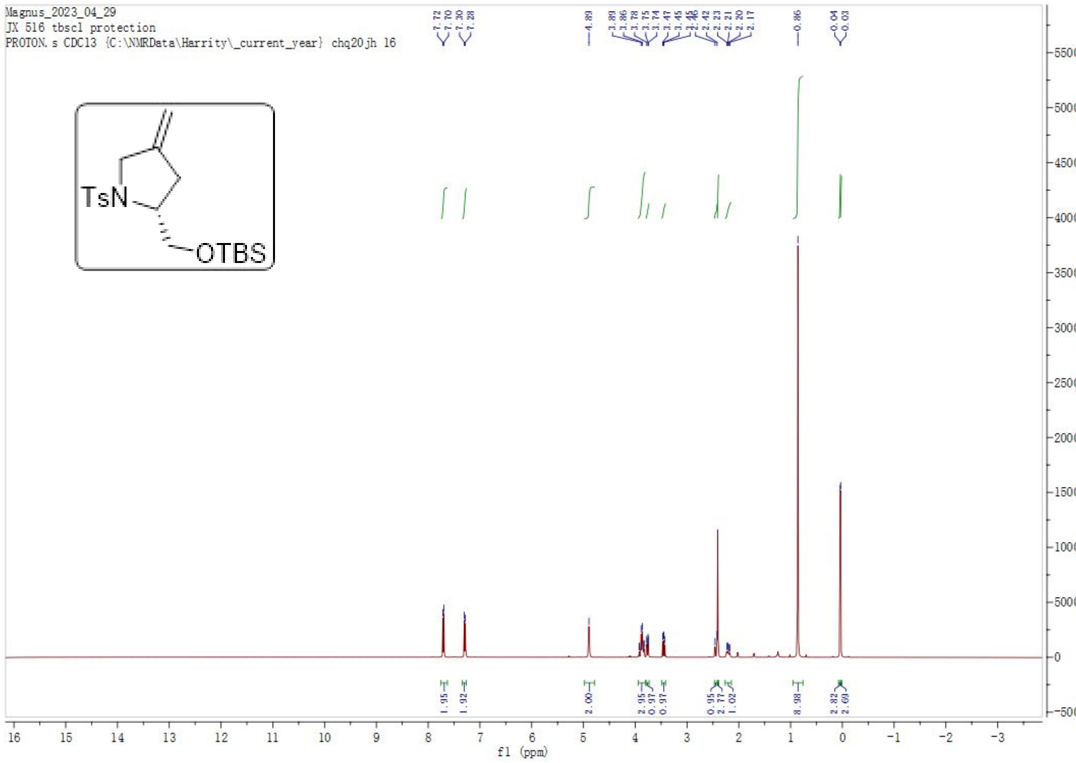

$^{13}C\{^{1}H\}$ NMR, $CDCl_3$, 101 MHz

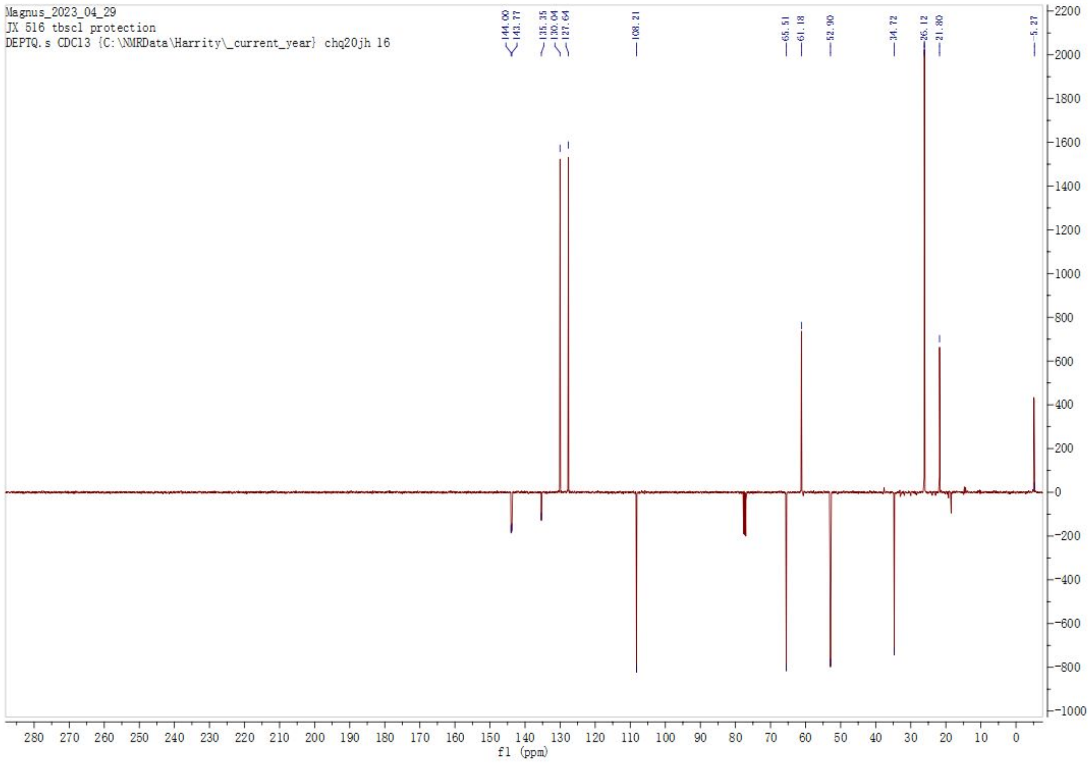

tert-butyl (*R*)-2-(((*tert*-butyldimethylsilyl)oxy)methyl)-4-methylenepyrrolidine-1-carboxylate （25）

^{1}H NMR, CDCl$_3$, 400 MHz

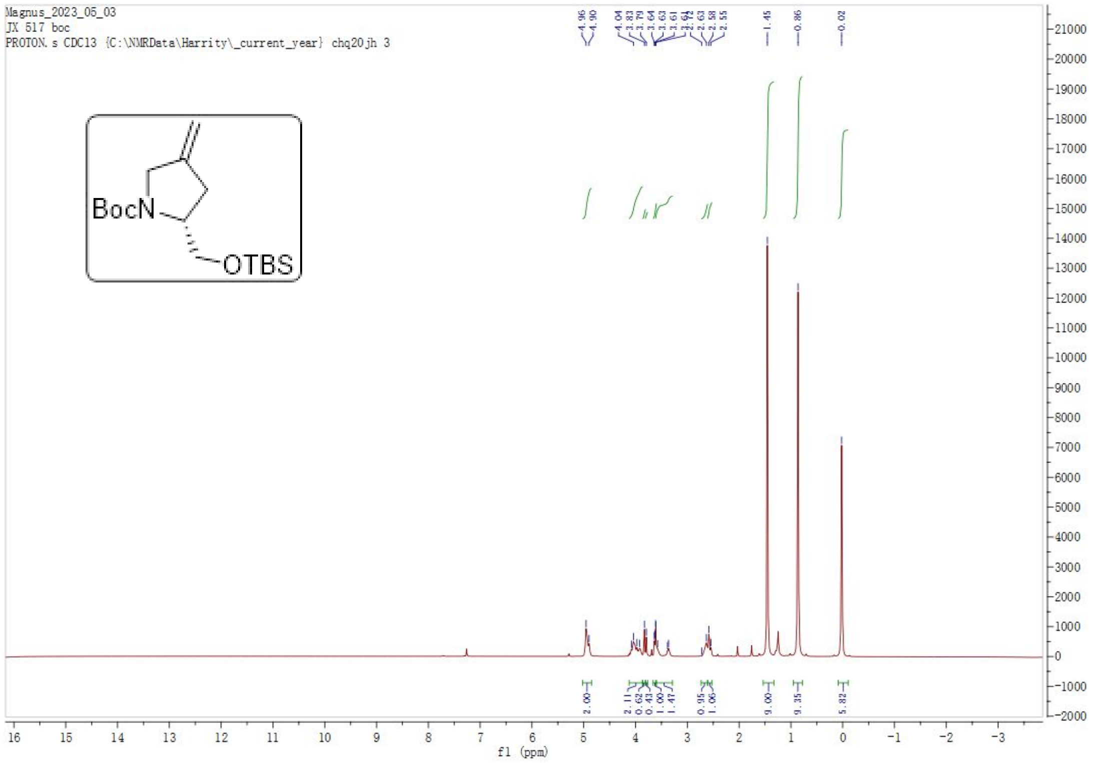

^{13}C{^{1}H} NMR, CDCl$_3$, 101 MHz

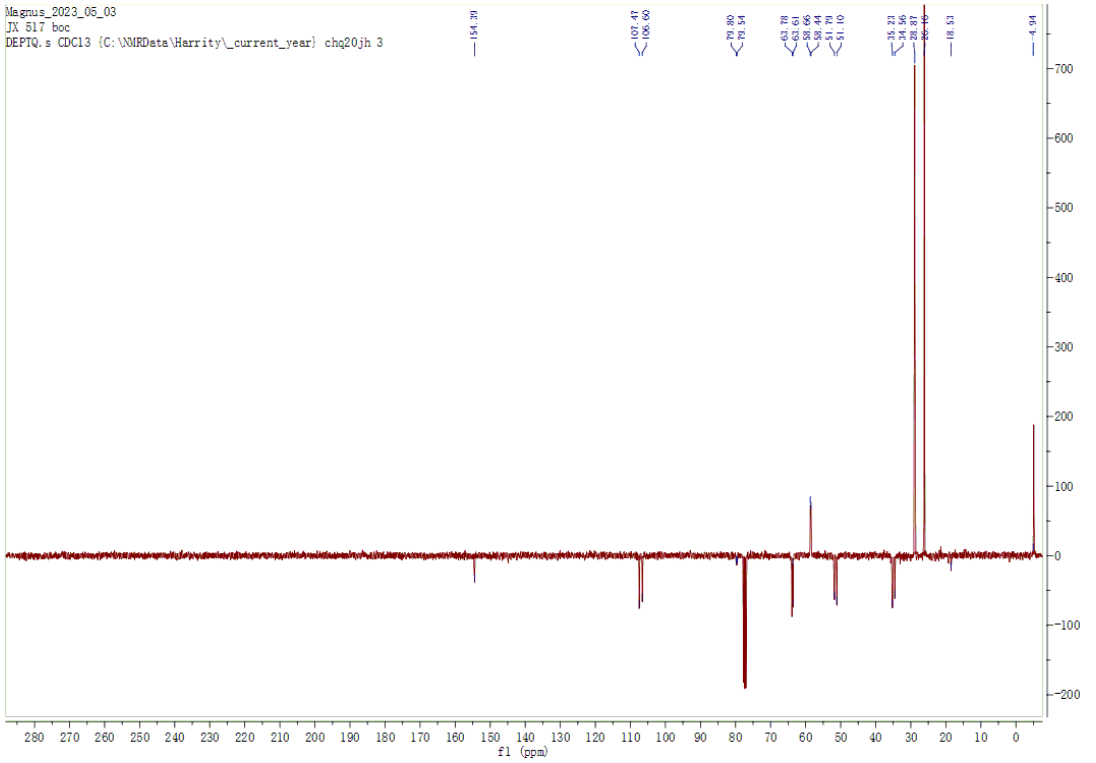

HPLC Traces

***tert*-butyl (*R*)-2-(((*tert*-butyldimethylsilyl)oxy)methyl)-4-methylenepyrrolidine-1-carboxylate (25**）

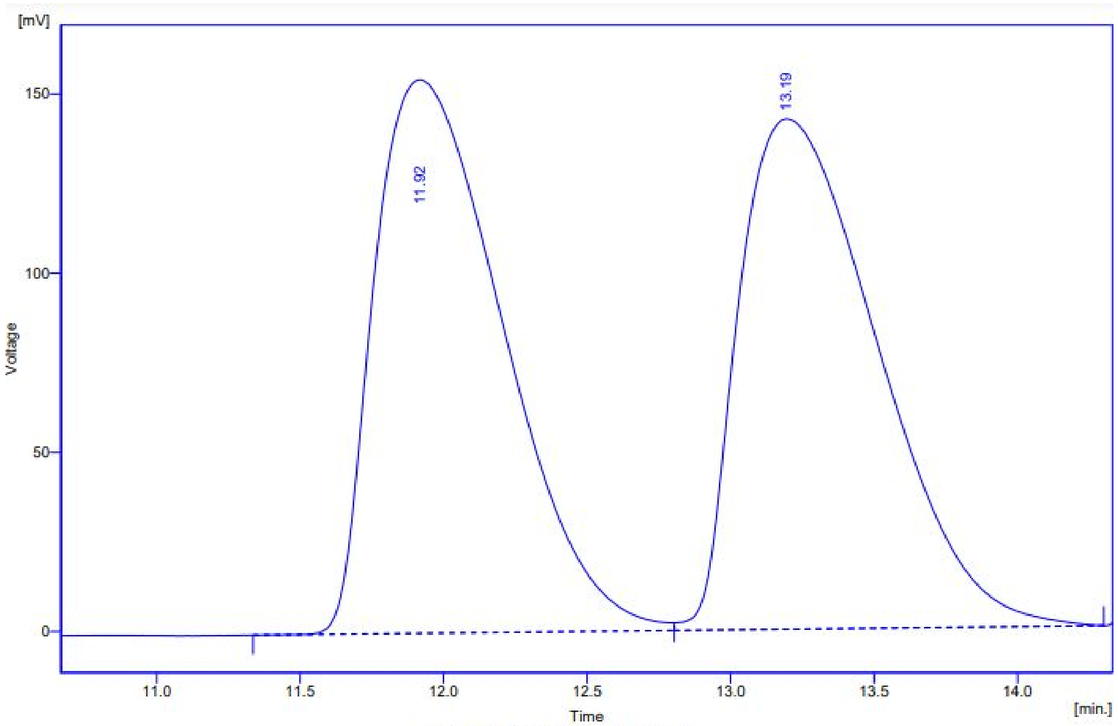

Result Table - Calculation Method Uncal

	Reten. Time [min]	Area [mV.s]	Height [mV]	Area [%]	Height [%]	W05 [min]
1	11.917	4818.220	154.491	50.1	52.0	0.51
2	13.193	4794.399	142.393	49.9	48.0	0.54
	Total	9612.619	296.885	100.0	100.0	

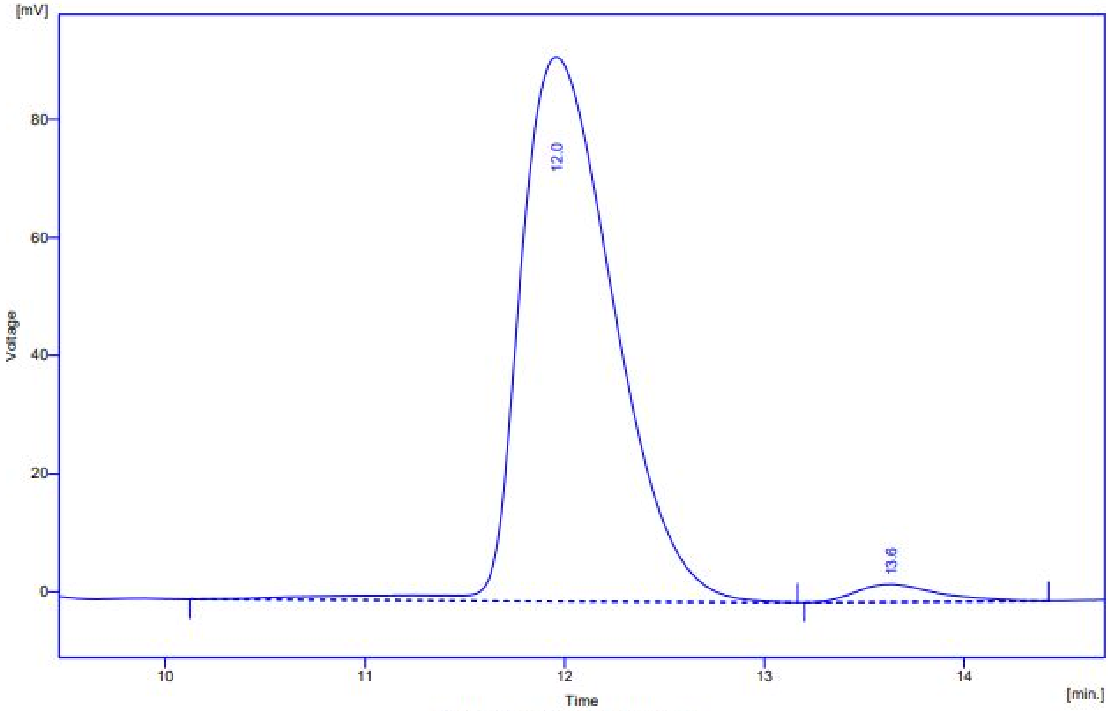

Result Table - Calculation Method Uncal

	Reten. Time [min]	Area [mV.s]	Height [mV]	Area [%]	Height [%]	W05 [min]
1	11.960	3000.966	92.031	97.3	96.9	0.51
2	13.637	84.261	2.952	2.7	3.1	0.43
	Total	3085.228	94.984	100.0	100.0	

Commercial (S) isomer

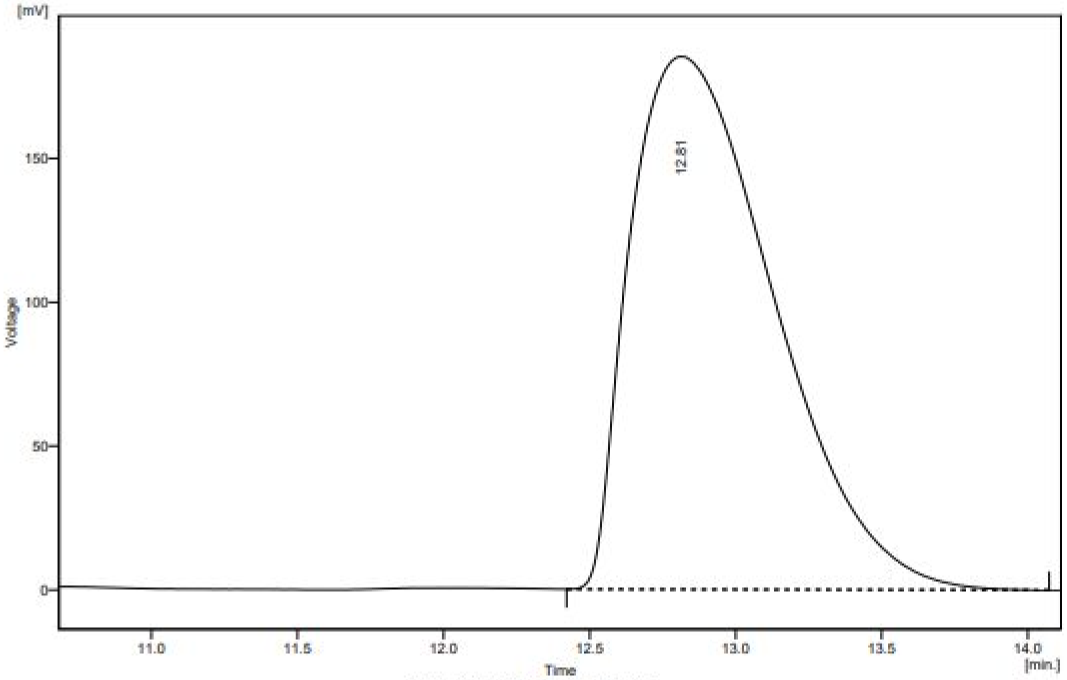

Result Table - Calculation Method Uncal

	Reten. Time [min]	Area [mV.s]	Height [mV]	Area [%]	Height [%]	W05 [min]
1	12.813	6341.739	185.231	100.0	100.0	0.55
	Total	6341.739	185.231	100.0	100.0	

Mixture of commercial (S) isomer + 25

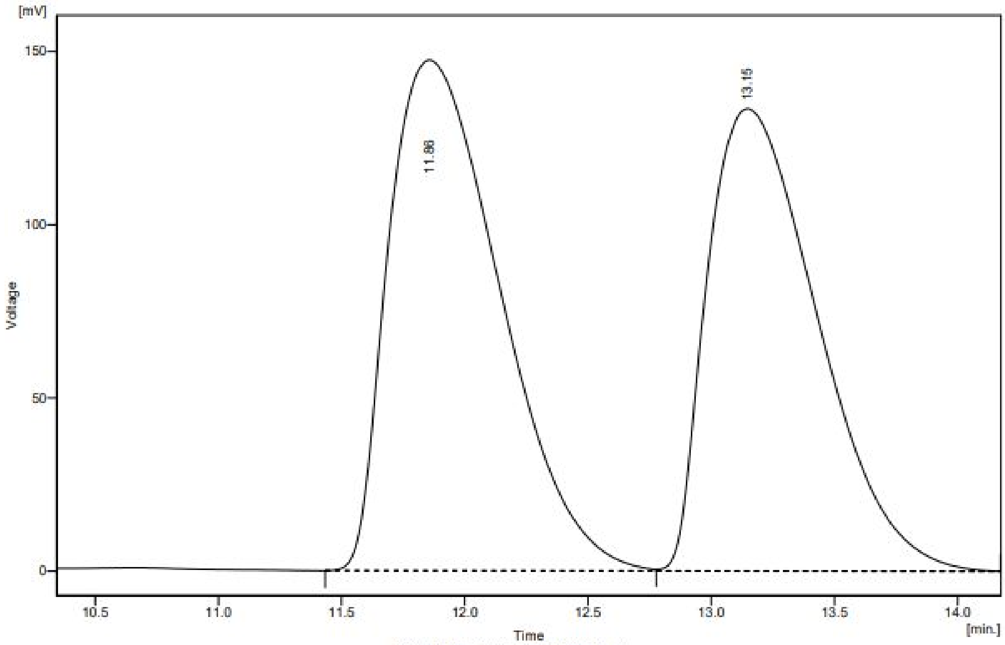

Result Table - Calculation Method Uncal

	Reten. Time [min]	Area [mV.s]	Height [mV]	Area [%]	Height [%]	W05 [min]
1	11.857	4629.961	147.357	52.8	52.5	0.51
2	13.147	4144.645	133.413	47.2	47.5	0.50
	Total	8774.607	280.769	100.0	100.0	

References

1. K. Pagire, S.; Kumagai, N.; Shibasaki, M., *ACS Cat.* **2021**, *11*, 11597–11606.
2. Dong, J.; Du, H.; Xu, J., *RSC Adv.* **2019**, *9*, 25034–25038.
3. Ushakov, P. Y.; Khatuntseva, E. A.; Nelyubina, Y. V.; Tabolin, A. A.; Ioffe, S. L.; Sukhorukov, A. Y., *Adv. Synth. Cat.* **2019**, *361*, 5322–5327.
4. Pramanik, M. M.; Yuan, F.; Yan, D. M.; Xiao, W. J.; Chen, J. R., *Org. Lett.* **2020**, *22*, 2639–2644.
5. Müller, P.; Fernandez, D.; Nury, P.; Rossier, J. C., *Helv. Chim. Acta.* **1999**, *81*, 935-945
6. Podrugina, T. A.; Alferova, V. A.; Mironov, A. V.; Matveeva, E. D.; Gleiter, R.; Zefirov, N. S., *Tetrahedron* **2016**, *72*, 6955–6962.
7. Sardini, S. R.; Lambright, A. L.; Trammel, G. L.; Omer, H. M.; Liu, P.; Brown, M. K., *J. Am. Chem. Soc.* **2019**, *141*, 9391–9400.
8. Del Valle, J. R.; Goodman, M., *J. Org. Chem.* **2003**, *68*, 3923-3931.
